# Few-atom-thick silver films for enhanced nanoscale nonlinear optics

**DOI:** 10.1038/s41467-026-74804-4

**Published:** 2026-07-01

**Authors:** Philipp K. Jenke, Saad Abdullah, Andrew P. Weber, Álvaro Rodríguez Echarri, Fadil Iyikanat, Vahagn Mkhitaryan, Frederik Schiller, J. Enrique Ortega, Philip Walther, F. Javier García de Abajo, Lee A. Rozema

**Affiliations:** 1 University of Vienna, Faculty of Physics, Vienna Center for Quantum Science and Technology (VCQ), Vienna, Austria; 2 University of Vienna, Vienna Doctoral School in Physics, Vienna, Austria; 3 ICFO-Institut de Ciencies Fotoniques, The Barcelona Institute of Science and Technology, Castelldefels (Barcelona), Spain; 4 Max-Born-Institut im Forschungsverbund Berlin e.V., (Berlin), Germany; 5 Center for Nanophotonics, NWO Institute AMOLF, Amsterdam, The Netherlands; 6 Centro de Física de Materials CSIC/UPV-EHU-Materials Physics Center, San Sebastián, Spain; 7 Donostia International Physics Center (DIPC), Donostia-San Sebastán, Spain; 8 Departmento de Física Aplicada, Universidad del País Vasco, San Sebastián, Spain; 9 University of Vienna, Research Platform for Testing the Quantum and Gravity Interface (TURIS), Vienna, Austria; 10 Christian Doppler Laboratory for Photonic Quantum Computer, Faculty of Physics, University of Vienna, Vienna, Austria; 11 ICREA-Institucio Catalana de Recerca i Estudis Avancats, Barcelona, Spain

**Keywords:** Two-dimensional materials, Nanophotonics and plasmonics, Nonlinear optics

## Abstract

The inherently weak nonlinear optical response of bulk materials remains a fundamental limitation in advancing photonic technologies. Nanophotonics addresses this challenge by tailoring the size and morphology of nanostructures to manipulate the optical near field, thus modulating the nonlinear response. Here, we explore a complementary strategy based on engineering the electronic band structure in the mesoscopic regime to enhance optical nonlinearities. Specifically, we demonstrate an increase in second-harmonic generation (SHG) from crystalline silver films as their thickness is reduced down to just a few atomic monolayers. Operating at the boundary between bulk and two-dimensional systems, these ultra-thin films exhibit a pronounced enhancement of SHG with decreasing thickness. This enhancement stems from quantum confinement effects that modify the interaction between electronic states and incident light, which we explain based on quantum-mechanical calculation. Our atomically-thin crystalline silver films provide a new means to overcome the small interaction volumes inherent to nanophotonic platforms, enabling efficient nanoscale nonlinear optics with potential applications in photonics, sensing, and quantum technologies.

## Abstract (web summary)

Achieving strong optical nonlinearities at the nanoscale remains a major challenge in photonics. Here, authors utilize quantum confinement in atomically thin crystalline silver films to significantly boost second-harmonic generation, overcoming classical scaling limits.

## Introduction

Optical nonlinear processes are central to a wide range of applications in photonics^1^, medicine and biology^2^, material science^3^, ultrafast optics^4^, and quantum technologies^5^. These applications typically rely on bulk nonlinearities in crystals^6,7^ or gases^8^, and require macroscopic interaction lengths to accumulate a substantial effect^9^. In addition, care must be taken in tailoring the properties of the medium to accurately satisfy phase-matching conditions^9^. In contrast, nonlinear optics in ultra-thin films offers the potential for integration with compact photonic devices and enables enhanced field confinement and stronger light-matter interactions within nanoscale volumes, reducing reliance on phase matching and enabling new regimes of nonlinear light generation.

In recent years, following a nanophotonics approach, great progress has been made in reducing the volume of the nonlinear medium while reaching an efficient nonlinear response by controlling light at the subwavelength scale^10–12^. This has been accomplished through extrinsic manipulations, such as plasmonic resonances^12–14^, strong electric field confinement^15^, and engineered metasurfaces^11,16–18^. Aside from these extrinsic manipulations, electronic band structure engineering can be used to precisely tailor a material's intrinsic electronic and optical properties. While such intrinsic manipulation is widely employed in platforms, such as semiconductors^16^, semiconductor quantum dots^19^, low-dimensional materials^20^, and ultracold atoms^21^, it is novel in the field of nonlinear nanophotonics and plasmonics. Moreover, the small interaction volume regime provides a natural setting with largely untapped potential for applying established techniques, such as electronic or magnetic field manipulation, atomic doping, or stress tuning, to control and enhance the nonlinear optical response.

Ultra-thin crystalline silver films are an exciting material platform where nanophotonic light control can be combined with mesoscopic band structure engineering^22,23^. These films can support extremely confined plasmonic modes in the visible and near-infrared regimes^14^, and intrinsic inelastic optical losses can be minimized to obtain spectrally narrow plasmons by maintaining a high-quality crystalline structure^14,22,24^. Moreover, quantum-well states (QWS) are observed in the electronic band structure when the thickness approaches a few atomic layers^22,23,25–27^, as experimentally confirmed by photoemission^28^ and electron tunneling^29^. These QWS are sensitive to atomic-scale thickness variations^26,27^ and can have a direct impact on the optical properties of atomically thin films. In particular, second-harmonic generation (SHG) has been predicted to oscillate as a function of the number of atomic layers^25–27^.

**Fig. 1:**
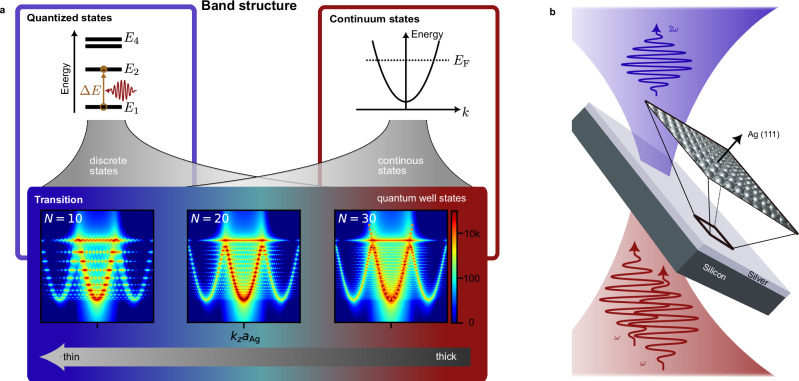
Interplay between electronic structure and nonlinear response in ultra-thin metal films. **a** The simulated electronic density of states^23^, represented here as a function of out-of-plane wave vector *k*_*z*_ (bottom, horizontal scale) and energy (vertical scale), shows an evolution from discrete quantum-well states (QWS) in thin films (*N* = 10 atomic monolayers (ML)) to a coalescing band at larger thicknesses (*t* = 30 ML). The discreteness of the electronic structure in the thin-film regime (upper-left scheme) enhances the nonlinear response (anharmonic excitation in response to harmonic light fields), in contrast to the more harmonic response associated with the parabolic band structure in the bulk limit (upper-right scheme). Here, *a*_Ag_ = 2.36 *Å* represents the atomic layer spacing. **b** Schematic of the optical transmission geometry used to characterize the SHG signal. The ultra-thin silver film is excited from the silicon substrate, and SHG is collected in transmission. The silver film is a single-crystal with (111) orientation grown on the far silicon surface. A protective silica capping layer covers the silver surface (not shown).

In this work, we explore the strong thickness-dependent nonlinear response of atomically thin crystalline silver films to enhance the thickness-normalized SHG conversion efficiency by almost two orders of magnitude. In particular, for film thicknesses *t* ≲ 30 monolayers (MLs), quantum effects dominate and segregate electronic states into discrete quantized levels, akin to semiconductor quantum dots, as shown in Fig. 1a^23,27^. Electrons are then confined to the QWS, resulting in discrete bands^28^ with less out-of-plane harmonic motion than in thicker crystalline structures, where electrons exhibit a parabolic out-of-plane dispersion relation. The subpanels of Fig. 1a show the simulated electronic density of states, where in ultra-thin silver films the *s*, *p* band is discretized along the *Γ*–L direction, yielding *N* quantum well levels for an *N*-layer film. Discretization is expected for thicknesses up to approximately 100 ML before reaching the continuous dispersion relation^30^ established when the intrinsic broadening of QWS exceeds their energy separation. The anharmonic motion associated with the confinement of QWS translates into a stronger nonlinear response while their bulk metal counterparts require stronger field strengths to reach a similar level of nonlinear response^23^.

Second-order nonlinearities at metallic interfaces were reported shortly after their observation in bulk crystals^6,31,32^, and have since been extensively studied^26,27,33–38^. However, they generally exhibit much lower nonlinear conversion efficiency than nonlinear crystals such as lithium niobate or borate compounds. This is primarily due to the vanishing bulk second-order susceptibility in centrosymmetric metals like silver, which has a face-centered cubic structure^9,36^. In such materials, second-order responses predominantly arise from symmetry breaking at surfaces or interfaces. However, when the interaction volume is constrained to be much smaller than the involved wavelengths (i.e., as it approaches the atomic scale), band structure engineering through the manipulation of surface properties, crystalline structure, and the use of nanostructures can dominate the linear and nonlinear optical responses^23,25,39^. Modifying such parameters in the mesoscopic regime has been proposed to produce substantial nonlinear efficiency enhancements^23^, potentially strong enough to compensate for the reduction of interaction volume.

Here, we demonstrate that QWS in long-term stable (compare Supplementary Fig. 6) wafer-scale ultra-thin crystalline silver can enhance thickness-normalized SHG conversion efficiencies by nearly two orders of magnitude while simultaneously reducing the interaction nonlinear volume. We have verified this observation across several excitation and SHG frequencies spanning the infrared to mid-infrared ranges, thereby quantifying the second-order nonlinear susceptibility. We have successfully modeled these effects based on a quantum mechanical description of the nonlinear response of silver film, going beyond previous classical nonlinear theories^36,40^. We provide both extensive material characterizations and quantitative nonlinear-optical characterizations^26,27,41,42^. In contrast to previous studies that rely on phenomenological QWS models^26,27,41,42^ and do not provide comprehensive structural characterization of the films ^27,38,41,42^, we combine high-quality epitaxial films, verified by detailed material characterization, with a quantitative analytical description of quantum-well-induced nonlinearities in noble-metal films. Moreover, several earlier works do not quantify SHG conversion efficiencies^27,41,42^, while others report SHG conversion efficiencies without extracting second-order nonlinear susceptibilities^26,27,41,42^. Our approach enables a consistent extraction of second-order nonlinear susceptibilities, establishing a direct and quantitative link between experiment and theory for SHG engineering in the mesoscopic regime of noble metals. By producing large-area crystalline films, we minimize inelastic losses, which are detrimental for SHG when decreasing the film thickness^38^. In our thin-crystalline metal platform, light-matter interaction could be further increased by introducing nanostructures for local field enhancement^15,43,44^, offering a new route for highly efficient nonlinear nanophotonics and plasmonics^23^, including dynamically tunable and reconfigurable nonlinear plasmonic systems that enable active control over harmonic generation^45^.

## Results

### Ultra-thin-film platform

**Fig. 2:**
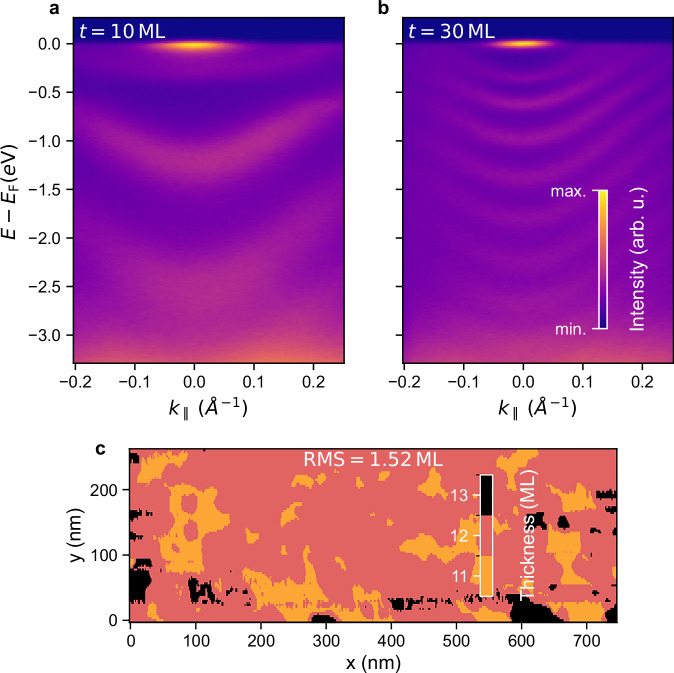
Film characterization. **a**,**b** Angle-resolved photoemission spectroscopy images of crystalline silver films with (111) crystal orientation showing QWS. The photoemission intensity is plotted relative to the Fermi energy *E*_F_ as a function of in-plane electron wave vector *k*_∥_. The film thickness *t* is expressed in terms of the number of (111) atomic monolayers (MLs): **a** 10 ML and **b** 30 ML. **c** Scanning tunneling microscope (STM) image over an extended area of a crystalline silver film with a root mean square roughness (RMS) of 1.52 ML for a 12 ML thick film.

Our material platform consists of crystalline silver films with (111) surface orientation and 10 to 30 atomic layers in thickness. The individual crystal domains may extend over a few square centimeters^22^. This large-area crystallinity is crucial for the formation of high-quality QWS, providing very low sheet resistance^22^, low inelastic losses, and high-quality plasmonic excitations, while remaining compatible with nanolithography and other nanophotonic manipulation techniques^14,46^. We employ epitaxial crystalline growth of silver on a crystalline silicon wafer (see Methods). This process is scalable and without lateral restrictions while preserving atomic-scale precision and low surface roughness (Fig. 2c). Our approach is more flexible than previously demonstrated wet-chemistry synthesis methods, in which the lateral film size is correlated to the thickness^47^.

As discussed in the Methods and SI, our silver films are grown in ultra-high vacuum (UHV), and characterized in-situ^22^. We first use angle-resolved photoemission spectroscopy (ARPES) to verify the film thickness by detecting and characterizing QWS. The observed changes in the band structure as a function of the film thickness are illustrated in Fig. 2a, b. Here, ARPES characterizes the in-plane wave vector *k*_∥_, as limited by the experimental apparatus, whereas Fig. 1a displays the simulated electronic density of states along the out-of-plane direction *k*_*z*_. QWS, depicted as horizontal lines in Fig. 1a, correspond to the energies at the center (*k*_∥_ = 0) of the two-dimensional bands shown in Fig. 2a, b. We then use scanning tunneling microscopy (Fig. 2c) to confirm the crystalline surface topography, indicating that we can produce flat films with atomic-scale terrace steps. Finally, before removal from UHV, our silver films are coated with a thin (≈1 nm) silicon passivation layer^14,22^, which oxidizes under ambient conditions to form silicon dioxide to ensure their stability under ambient conditions. We then remove the films from UHV and measure the SHG response for different film thicknesses (Fig. 1b) to probe the optical nonlinear response.

### Measured SHG signal

**Fig. 3:**
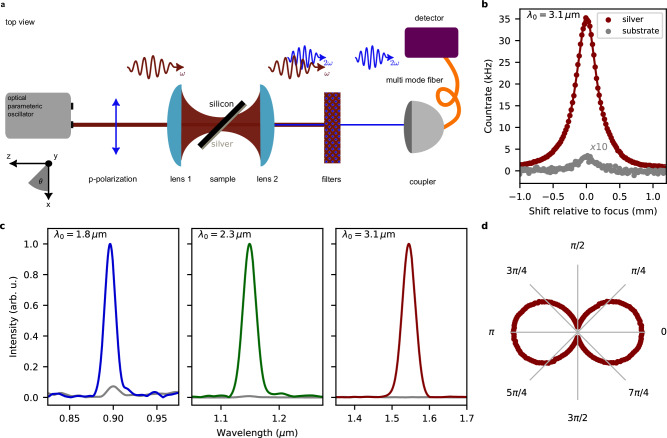
Characterization of the SHG signal. **a** Sketch of the optical transmission setup: A Ti:Sapphire oscillator (not shown) pumps an optical parametric oscillator providing p-polarized long excitation pulses (200 fs), which are focused via the first lens 1 into the 45° tilted sample. The generated SHG signal is collimated by lens 2, and optical filters spectrally separate it from the excitation light. The isolated SHG signal is then coupled into a multi-mode fiber and directed to the detector. **b** Detected SHG count rate from two different lateral sample positions corresponding to the bare silicon substrate (multiplied by 10) and the deposited silver film in gray and red, respectively. **c** Spectra of the SHG signal for different excitation wavelengths *λ*_0_. **d** SHG for fixed p-polarization excitation and different angles of the polarizer to the right of the sample, with 0° corresponding to p-polarization. The silver film is 17 ML thick in (**b–d**).

To isolate the nonlinear SHG signal from the silver films, we use a modified focus scan setup (Fig. 3a). In our configuration, the 45°-tilted sample is scanned through the focal point of a femtosecond-pulsed mid-infrared pump beam (with a center wavelength *λ*_0_ of 1.8, 2.3 or 3.1 μm). The resulting SHG signal (at 0.9, 1.15 or 1.55 μm, respectively) is measured in transmission as detailed in Methods. The sample was tilted to break the symmetry of the crystal structure and enable SHG, while the excitation angle was technically limited. To avoid absorption of the SHG signal by the silicon substrate, the sample is oriented so that the pump traverses the substrate before interacting with the silver film. All optical measurements are conducted under ambient pressure and temperature conditions within several days after fabrication, with no sample degradation observed for weeks (Supplementary Fig. 6). This underscores the efficacy of the passivation layer, and the durability and resilience of the fabricated films.

A representative focus-scan measurement is presented in Fig. 3b, demonstrating notable SHG signal strengths only at the focal point with the highest fluence (red data), and essentially no signal from the substrate (gray points). This allows us to conclude that neither SHG from the silicon surface nor quadrupole contributions play a substantial role^48^, while the contribution from the capping layer is expected to be substantially lower^49^. In Fig. 3c, we plot the spectra of the measured nonlinear signal at the focal point for pump wavelengths of 1.8, 2.3, and 3.1 μm. These spectra reveal clear peaks at the expected SHG wavelengths, verifying the nonlinear process spectrally.

We next study the polarization behavior of the nonlinear signal. The generated SHG is co-polarized with the incoming p-polarized light, as shown in Fig. 3d. This strongly polarized signal indicates a coherent nonlinear process. We did not perform a study of the SHG dependence on the linear polarization of the excitation, since excitation through the silicon substrate preserves only p-polarization. A purely s-polarized excitation cannot be achieved because p-polarized components remain after transmitting through the substrate (characterized in Supplementary Fig. 15), and the resulting SHG signal is reduced compared to the p-polarized excitation. The limitations of polarization tomography restrict access to extracting individual components of the second-order nonlinear susceptibility tensor.

**Fig. 4:**
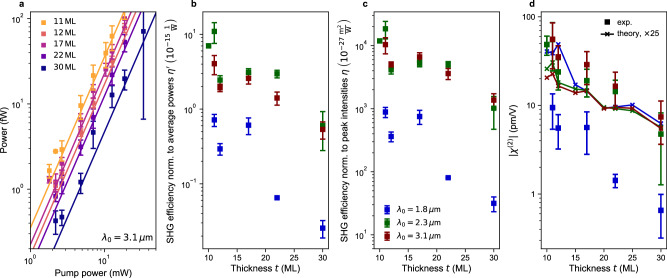
SHG efficiencies for different silver film thicknesses. **a** Power scaling of the measured SHG signal (symbols) in crystalline silver films of different film thicknesses *t* for a fixed incident wavelength of *λ*_0_ = 3.1 μm. A second-order power law is used to fit the data. **b** SHG conversion efficiencies $\eta {\prime}$ for various excitation wavelengths *λ*_0_ and film thicknesses. Experimental efficiencies (squares) refer to average powers measured before and after the sample layer stack and are extracted from the power scaling in (**a**) without normalization. **c**, SHG conversion efficiencies *η* for various excitation wavelengths *λ*_0_ and film thicknesses. Efficiencies refer to peak intensities measured before and after the sample layer stack and are extracted from (**b**). **d** SHG susceptibility of the silver film. The theoretical values are calculated assuming an intrinsic damping of *γ* = 0.1 eV and multiplied by 25 for comparison, while the experimental values are extracted from the data shown in (**c**). Error bars represent the standard deviation.

To quantify the conversion efficiency and confirm the second-order nature of the SHG signal, we measure the average SHG power *P*_SHG_ as a function of average excitation power *P*_0_ at pulsed excitation just before and after the sample. Results for an excitation wavelength of 3.1 μm are presented in Fig. 4a for various silver film thicknesses. Analogous measurements for excitation wavelengths of 1.8 and 2.3 μm are provided in Supplementary Fig. 5.

A quadratic dependence with a slope of 2 on a log-log scale is observed in Fig. 4a, confirming the second-order power scaling characteristic of the SHG process, while we corrected for saturation and background counts. As detailed in the Methods section, the regions affected by the detector nonlinearity can be well-modeled (see Methods and Supplementary Fig. 5), enabling the extraction of the actual average SHG power *P*_SHG_ from the measured average detector power *P*_det_. The SHG conversion efficiency normalized to the average power, $\eta ^{\prime}={P}_{{{\rm{SHG}}}}/{P}_{{{\rm{0}}}}^{2}$, is the single free parameter used to fit each power-dependence dataset in Fig. 4a and is presented in Fig. 4b for all excitation wavelengths. The SHG conversion efficiency $\eta ^{\prime}$ for the full layer stack can be normalized to the peak intensity *I*_peak_ (see Methods for the conversion). It is defined as $\eta={I}_{{{\rm{SHG,peak}}}}/{I}_{{{\rm{0,peak}}}}^{2}$ and is shown in Fig. 4c. The experimental uncertainty in Fig. 4 arises from spatial inhomogeneities of the films (statistical variations across probed regions) and fluctuations of the excitation beam, except at low SHG signal levels for thicker films, where detector noise becomes significant. For all considered excitation wavelengths, we observe an increase in SHG conversion efficiency $\eta ^{\prime}$ with decreasing film thickness, even without any thickness or volume normalization. We observe conversion efficiencies *η* that are of the same order of magnitude as those reported in a previous work^38^, which does not report an increase for thinner films due to the use of evaporated, non-crystalline films. In contrast, smaller conversion efficiencies are observed in a separate work^26^, which focuses on crystalline films. We summarize this comparison in Supplementary Table 3. From a standard nonlinear optics perspective, this trend is counterintuitive, as a quadratic increase in SHG intensity with interaction length would be expected for a non-phase-matched process with short propagation lengths^9^. In contrast, we observe the opposite trend: an increase in nonlinear conversion with decreasing thickness, caused by a more anharmonic electron motion associated with QWS (i.e., far from harmonic motion in a parabolic potential), as they become increasingly more separated in energy. While previous work has reported oscillations in SHG conversion efficiency *η* with film thicknesses^26,27^, here we instead observe an increase without pronounced oscillations. This indicates that, under our film growth and measurement conditions, the SHG response is primarily governed by a non-oscillatory contribution that increases with decreasing thickness and dominates the oscillatory contributions associated with QWS resonances or thickness-dependent phase interferences. At an excitation wavelength of *λ*_0_ = 1.8 μm, we achieve an enhancement of the thickness-normalized SHG conversion efficiency *η*/*t* by almost two orders of magnitude when decreasing the film thickness from 30 ML to 11 ML. This decrease in thickness produces a drastic boost in conversion efficiency, which is observed for the first time in this system, well beyond skin-depth effects, showing the potential of band structure engineering in this novel nanophotonic platform. The skin-depth of silver at the optical wavelength of  ~ μm is of the order of 10 nm, therefore a few-atom-thick silver films (below 50 ML) act as a two-dimensional metallic screen. Based on the idea of having a two-dimensional structure generating radiation at the second harmonic, we obtain an expression for the resulting second harmonic susceptibility ${\chi }_{b}^{(2)}$ in Fig. 4d (see derivation in the Supplementary Note 1 and comparison to other works in Supplementary Table 4):

1 $$\left\vert {\chi }_{b}^{(2)}\right\vert=\frac{{\left\vert {E}_{0}/{E}_{z}\right\vert }^{2}}{\left\vert 1+{r}_{p}^{{{\rm{Air\to Si}}}}\right\vert }\frac{\sqrt{(c/2\pi )\cos {\theta }_{0}\,\eta }}{4\pi \,kt\tan {\theta }_{0}},$$

in Gaussian units, as a function of the measured *η*(*t*, *λ*) efficiencies, and the ratio of the impinging p-polarized electric field driving the nonlinear response ${E}_{z}/{E}_{0}={t}_{p}^{{{\rm{Air\to Si}}}}\,{t}_{p}^{{{\rm{Si\to Air}}}}\,\sin {\theta }_{0}$ traversing the substrate, with *r* and *t* the corresponding reflection and transmission coefficients, respectively.

### Theoretical model and origin of SHG

To validate our experimental results, we model the silver layers as a continuous distribution of out-of-plane second-harmonic induced dipoles. We based our calculations on a quantum-mechanical description of QWS^23^ using a film-confining model potential, fitted to reproduce salient features of the silver surface electronic structure; a nonlinear extension of the random-phase approximation is then followed; although the model potential is taken to be translationally invariant along surface directions, we incorporating band-dependent effective electron masses from density-functional theory simulations including in-plane atomic corrugation (see ref. ^23^. and Methods). We compare the experimental and theoretical second-order nonlinear susceptibility $\left\vert {\chi }_{b}^{(2)}\right\vert$ in Fig. 4d for the considered excitation wavelengths of 1.8, 2.3, and 3.1 μm. The calculated second-order susceptibilities exhibit a marked increase as the number of layers decreases (Fig. 4d), in agreement with the trend observed experimentally. Our calculations with additional thicknesses partially capture the well-known oscillatory behavior on the linear scale shown in Supplementary Fig. 12. It should be noted, however, that a more comprehensive theoretical treatment would be required to properly reproduce the detailed layer-by-layer oscillatory behavior. The present calculations are intended to capture the dominant increasing trend in the nonlinear response as the thickness decreases. Therefore, these results indicate that the increase in second-order susceptibility arises from thickness-dependent changes across the entire film. The observed changes arise from intrinsic factors, such as modifications to the QWS band structure, symmetry-breaking regions, and quantum confinement, as well as extrinsic mechanisms, such as field enhancement and reduced fundamental reabsorption.

Therefore, these results indicate that the increase in the second-order susceptibility arises from thickness-dependent modifications across the entire film. The observed behavior originates from intrinsic factors, such as changes in the QWS electronic structure, surface and interface symmetry breaking, and quantum confinement, as well as extrinsic effects including optical field enhancement and reduced reabsorption of the fundamental field.

In ultra-thin metallic films, the electromagnetic field probes the entire Ag film thickness because of the finite optical penetration depth at the excitation wavelengths used in this work. Therefore, the nonlinear response must be understood to arise from the entire film, whose electronic structure is strongly modified by thickness, dielectric environment, and interfacial symmetry breaking. Intrinsically, SHG originates from regions where inversion symmetry is broken, namely at the Ag–air and Ag–Si interfaces, both of which contribute simultaneously to the measured signal. In the ultra-thin crystalline limit, quantum confinement further enhances the intrinsic nonlinear response by modifying the parity, spacing, and anharmonicity of the quantized electronic subbands, thereby increasing the effective surface nonlinear susceptibilities.

At the same time, extrinsic mechanisms reinforce this enhancement: reducing the film thickness decreases reabsorption of both the fundamental and SHG fields and improves the linear optical response, including stronger local-field enhancement. While intrinsic mechanisms primarily modify the nonlinear susceptibility through symmetry breaking and quantum-well-induced electronic restructuring, extrinsic mechanisms act by enhancing local optical fields and the effective interaction length, thereby increasing the observable SHG yield without altering the microscopic origin of *χ*^(2)^. These intrinsic and extrinsic effects act cooperatively, providing a consistent physical picture for the observed increase of SHG efficiency with decreasing Ag film thickness.

Access to the different mechanisms could be achieved in the future through polarization-resolved and thickness-dependent SHG characterization of the films.

However, the variation in the calculated second-order nonlinear susceptibility with incident-light wavelength differs from the experimental results.

This discrepancy originates from the assumptions underlying the theoretical model. In particular, the model employs parabolic quantum-well subbands, neglects the substrate and assumes a single, energy-independent linewidth. While the effective masses used in the model are extracted from density-functional theory (DFT) calculations by fitting the low-energy dispersion of the quantum-well states to parabolas, the full DFT band structures reveal deviations from a harmonic behavior. The effective-mass approximation is therefore another important source of quantitative uncertainty: the SHG response is resonantly sensitive to the energy spacing and curvature of the quantum-well states, so small deviations from parabolicity can shift or reshape the calculated spectral features^50^. Moreover, the interaction with the Si substrate modifies the band dispersions and effective masses, thereby altering the carrier dynamics. A further limitation of the model is that the response of the deeper *d* bands is included only through an effective dielectric background rather than through a fully atomistic description. This approximation captures the dominant screening contribution of core electrons, but it cannot reproduce possible facet-, thickness-, or interface-dependent modifications of the interband response. ARPES data in Fig. 2 further demonstrate that the electronic linewidths are subband- and energy-dependent rather than uniform. Although surface-enabled Landau damping is implicitly captured in the model through an effective phenomenological linewidth, its detailed energy-, subband-, and thickness-dependent character is not explicitly resolved. The use of a homogeneous damping rate can therefore affect both the amplitude and the spectral width of the calculated SHG resonances. Since the SHG response is highly sensitive to carrier density, subband energies, effective masses, core-electron screening, and electronic lifetimes, quantitative deviations in both the magnitude and spectral position of the SHG features are therefore expected. In addition, thickness-dependent ultrafast thermal and electronic broadening effects in the silver film^51^ and the environment, which are not included in the present model, may further contribute to the observed differences between theory and experiment (see Supplementary Note 3). We emphasize that the purpose of the present model is to capture the dominant physical trends governing the thickness dependence of the SHG response rather than to provide a fully quantitative description. A more complete treatment would necessitate substantially more complex theoretical and computational methods, including realistic nonparabolic band structures, an atomistic treatment of *d*-band screening, and state-resolved damping, which lies beyond the scope of the present work.

## Discussion

We have demonstrated that crystalline ultra-thin silver films are a promising platform for nonlinear nanophotonics, holding the potential to enhance optical nonlinear interactions while simultaneously reducing the interacting volume. Due to the quantum-well band structure, ultra-thin films overcome the classical scaling of the nonlinear intensity with the interaction length, resulting in stronger nonlinear responses as the film thickness decreases. This approach to enhancing the nonlinear response is compatible with nanophotonic strategies, such as embedding in optical cavities, which could work well in the visible and mid-infrared spectral ranges.

While the nonlinear conversion efficiencies observed in silver films are still lower than those in other 2D materials, such as transition metal dichalcogenides, they offer advantages in terms of stronger optical response, more flexible nanopatterning, and higher thermal and electrical conductivities associated with their metallic nature. In particular, lateral patterning can enhance the linear response^14^, and in turn, also the nonlinear behavior in virtue of the associated optical field confinement and enhancement. In this context, graphene plasmons have created great expectations because of their appealing properties^52^, including strong nonlinearities^53^, but they are constrained to the mid-infrared range. In contrast, silver films cover a plasmonic behavior extending to the near-infrared and visible domains^14,23^. Plasmon resonances, nanostructures, and electric field enhancements have the potential to boost the conversion efficiency to reach nonlinear strengths comparable to those of 2D materials while maintaining electronic tunability^12,54,55^. In addition, theory predicts even greater enhancements for film thicknesses below 10 ML^23^, offering exciting prospects as advances in fabrication continue to improve the stabilization of such ultra-thin films over large areas.

## Methods

### Experimental details

We conducted SHG measurements using a modified focus scan setup, where the SHG signal was measured as the sample was traversed along the optical axis through the focus of the excitation beam (Fig. 3a), while different lateral spots were excited by moving the tilted sample within the focal plane. Our incident light beam comprised linearly p-polarized Gaussian pulses, typically *τ* = 200 fs in duration, with a tuble center wavelength *λ*_0_ ranging from 1.8 μm (0.69 eV) to 3.1 μm (0.40 eV) (Supplementary Fig. 7) at a repetition rate *f* = 76 MHz. This beam was generated by an optical parametric oscillator optically pumped by a Ti:Sapphire oscillator. The pulse duration and repetition rate varied slightly due to daily alignment.

The maximum acquisition time for SHG measurements before readjusting the excitation power was around 20 to $30\,\min$; during this time, we observed power fluctuations of  < 1 %. A half-wave plate was employed to rotate the polarization of the incoming beam, and linear polarization was ensured by a polarizer (p-polarization was used, unless otherwise stated). An optical system comprising two lenses, each with a 20 mm focal length, was used to focus the excitation beam onto the sample and collimate the signal of interest. The beam waist *σ*_0_ for the excitation wavelengths of 1.8, 2.3, and 3.1 μm were 11.8, 13.8, and 17.0 μm, respectively (Supplementary Fig. 9). The sample was excited from the back side to prevent the absorption of the nonlinear signal at the substrate. Most of the nonlinear emission was generated at the focal plane, where fluence was maximum, refracting the technical-limited incident angle from *θ*_0_ = 45° in free space to *θ*_1_ ≈ 12° inside the silicon substrate. After the collimation lens, the nonlinear signal was isolated by optical transmission filters and coupled into a multimode fiber before being detected with InGaAs or Si single-photon detectors. The latter was only used for 1.8 μm excitation wavelength. Excitation and detection efficiencies have been taken into account. We confirmed the spectral characteristics of the signal through spectral verification using an interferometer-based spectrometer from Nireos by Fourier transforming the autocorrelation function of the interferogram (Supplementary Fig. 10).

### Extracting the second-order nonlinear susceptibility

The average SHG power, *P*_SHG_, was related to the average excitation power *P*_0_ by assuming a second-order power law:

2 $${P}_{{{\rm{SHG}}}}=\eta ^{\prime} {P}_{0}{(\omega )}^{2},$$

where $\eta ^{\prime}$ is the fitted conversion efficiency extracted from Fig. 4a and shown in Fig. 4b. In our analysis, we also account for a constant background offset *P*_dark_ and detector saturation effects, using the expression:

3 $${P}_{{{\rm{det}}}}=\frac{{P}_{{{\rm{SHG}}}}}{1+{P}_{{{\rm{SHG}}}}/{P}_{{{\rm{sat}}}}}+{P}_{{{\rm{dark}}}},$$

which allows us to extract *P*_SHG_ from the measured detector signal *P*_det_ and the fitted value of *P*_sat_.

The SHG conversion efficiency is defined by $\eta={I}_{{{\rm{SHG,peak}}}}/{I}_{{{\rm{0,peak}}}}^{2}$ and the peak intensity is given by ${I}_{{{\rm{peak}}}}=\frac{{f}_{{{\rm{shape}}}}}{f\tau {A}_{2\omega }}{P}_{{{\rm{ave}}}},$ where ${A}_{2\omega }=\pi {\left({\sigma }_{0}/\sqrt{2}\right)}^{2}\sec {\theta }_{0}$ is the focal area, *f*_shape_ = 94% accounts for the Gaussian pulse shape, and we assumed a reduction of the beam waist by a factor of $\sqrt{2}$ for the SHG.

### Fabrication and characterization details

Si-passivated Ag films were prepared under ultra-high vacuum conditions on 525 μm thick Si(111) substrates following the procedures in ref. ^22^. The crystallinity, atomic flatness, and thickness of the Ag films were characterized by low-energy electron diffraction (Supplementary Fig. 1), ARPES (Fig. 2a, b and Supplementary Fig. 2), STM (Fig. 2c), and X-ray photoelectron spectroscopy (XPS) (Supplementary Fig. 3) before removal from UHV. SEM (see Supplementary Fig. 4) was used to confirm the flatness and absence of dewetting in the passivated film after air exposure. Photoemission measurements were performed using a SPECS Phoibos 150 electron analyzer. For ARPES measurements, a monochromatized He gas discharge lamp operating at the He I*α* excitation energy (21.22 eV) was used. A monochromatized Al K*α* source (1486.71 eV) was used for XPS measurements. The experimental energy resolution was better than 40 meV and 500 meV for ARPES and XPS, respectively. The angular resolution for ARPES was better than 0.4°. STM data were collected using an Omicron VT setup. The sample was kept at a temperature of 100 K for the photoemission measurements and at room temperature (300 K) for the LEED, STM, and SEM measurements. The influence of the capping layer on the film was investigated in our previous work^22^.

### Density functional theory calculations

We conduct first-principles density functional theory (DFT) calculations on Ag(111) films of varying thicknesses. The Kohn-Sham one-electron electronic structure is computed using the Perdew-Burke-Ernzerhof (PBE) exchange-correlation functional^56^, as implemented in the Vienna ab initio simulation package (VASP)^57,58^. The plane wave cutoff energy is set to 600 eV. The experimentally determined bulk lattice constant (4.09 *Å*) is used to construct thin silver films by fixing the interatomic bond distances. A 18 × 18 × 1 *Γ*-centered *k*-point mesh is used for Brillouin zone sampling. Each Ag film is positioned at the center of the simulation cell, with a vacuum gap of 10 *Å* in the vertical direction to avoid spurious interactions between adjacent cells. Based on the self-consistent charge density, we extract single-particle energies throughout the Brillouin zone to obtain the electronic band structures of the layers. Effective masses near the Fermi level are then determined using the methodology described in our previous work^23^.

### Quantum-mechanical description of silver films

Following the prescription described in ref. ^23^, we simulate the nonlinear optical response of few-atom-thick films of Ag(111) based on a single-particle wave function formalism, where electrons are subject to an atomic potential that reproduces the main features of the measured silver surface (work function, projectec gap, etc.). From the resulting thickness-dependent wave functions, we compute the induced dipole per unit area p under excitation by an out-of-plane optical electric potential *ϕ* = − *zE*_0_ determined by the normal electric field amplitude *E*_0_ under the illumination conditions. We solve the problem in one dimension and obtain a second-harmonic normal dipole per unit area, from which we determine the SHG susceptibility as ${\chi }^{(2)}={p}_{z}/(t{E}_{0}^{2})$, where *t* is the thickness.

## Supplementary information

Supplementary Information

Transparent Peer Review file

## Source data

Source Data

## Data Availability

The data generated in this study are provided in the Supplementary Information/Source Data file. Source data are provided with this paper.

## Code availability

The codes that support the plots within this paper and other findings of this study are available from the corresponding author upon request.
