# Supplementary material for: Few-atom-thick silver films for enhanced nanoscale nonlinear optics
Source: Nat Commun. 2026 Jul 1;17:8214. doi: 10.1038/s41467-026-74804-4 (PMC13463075; doi:10.1038/s41467-026-74804-4)
Supplement: Supplementary file 1 [file 41467_2026_74804_MOESM1_ESM.pdf]

Few-atom-thick silver films for enhanced nanoscale nonlinear optics

– SUPPLEMENTARY MATERIAL –

Philipp K. Jenke†,[1,2] Saad Abdullah†,[3] Andrew P. Weber,[3] Álvaro Rodríguez Echarri,[4,5] Fadil Iyikanat,[3] Vahagn Mkhitaryan,[3] Frederik Schiller,[6,7] J. Enrique Ortega,[6,7,8] Philip Walther,[1,9,10] F. Javier García de Abajo*,[3,11] and Lee A. Rozema*[1]

[1] *University of Vienna, Faculty of Physics, Vienna Center for Quantum Science and Technology (VCQ), Boltzmanngasse 5, 1090 Vienna, Austria*

[2] *University of Vienna, Vienna Doctoral School in Physics, Boltzmanngasse 5, 1090 Vienna, Austria*

[3] *ICFO-Institut de Ciencies Fotoniques, The Barcelona Institute of Science and Technology, 08860 Castelldefels (Barcelona), Spain*

[4] *Max-Born-Institut im Forschungsverbund Berlin e.V., Max-Born-Straße 2a, 12489 (Berlin), Germany*

[5] *Center for Nanophotonics, NWO Institute AMOLF, 1098 XG Amsterdam, The Netherlands*

[6] *Centro de Física de Materials CSIC/UPV-EHU-Materials Physics Center, 20018 San Sebastián, Spain*

[7] *Donostia International Physics Center (DIPC), 20018 Donostia-San Sebastián, Spain*

[8] *Departmento de Física Aplicada, Universidad del País Vasco, 20018 San Sebastián, Spain*

[9] *University of Vienna, Research Platform for Testing the Quantum and Gravity Interface (TURIS), Boltzmanngasse 5, 1090 Vienna, Austria*

[10] *Christian Doppler Laboratory for Photonic Quantum Computer, Faculty of Physics, University of Vienna, 1090 Vienna, Austria*

[11] *ICREA-Institucio Catalana de Recerca i Estudis Avancats, Passeig Lluis Companys 23, 08010 Barcelona, Spain*

(Dated: June 11, 2026)

† *These authors contributed equally.*

∗ *Corresponding authors: lee.rozema@univie.ac.at, javier.garciadeabajo@nanophotonics.es*

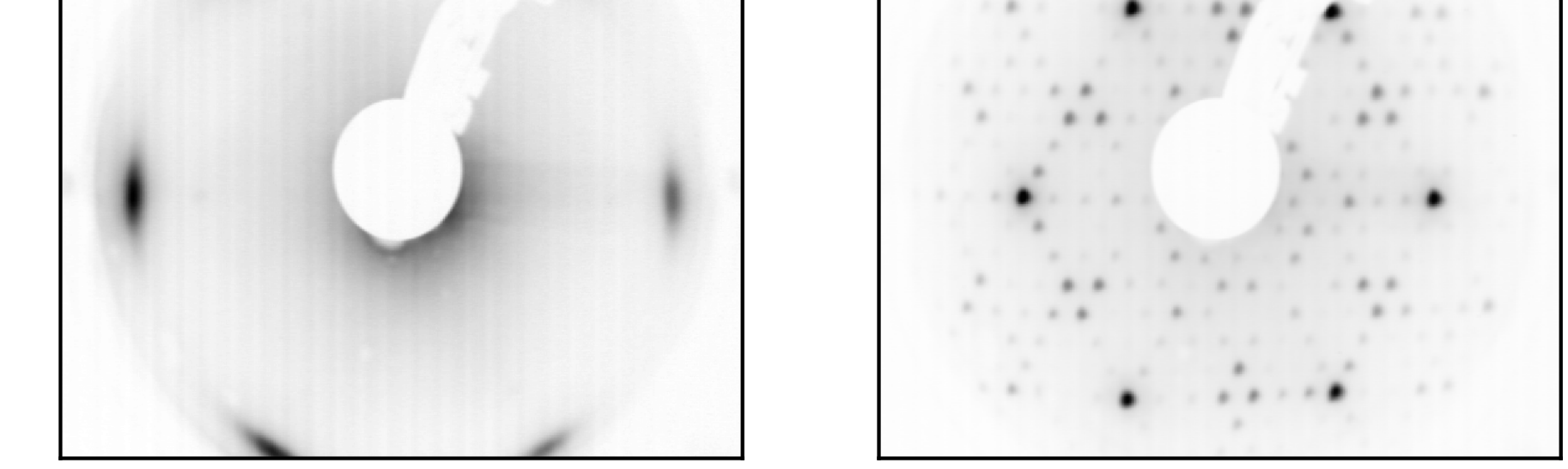

FIG. S1. **Low-energy electron diffraction images** of **a** the crystalline Ag film freshly deposited on the Si(111) substrate and **b** the bare substrate prior to deposition. The electron energy was 51 eV in both cases. The absence of substrate spots in **a** confirms uniformity of the film coverage.

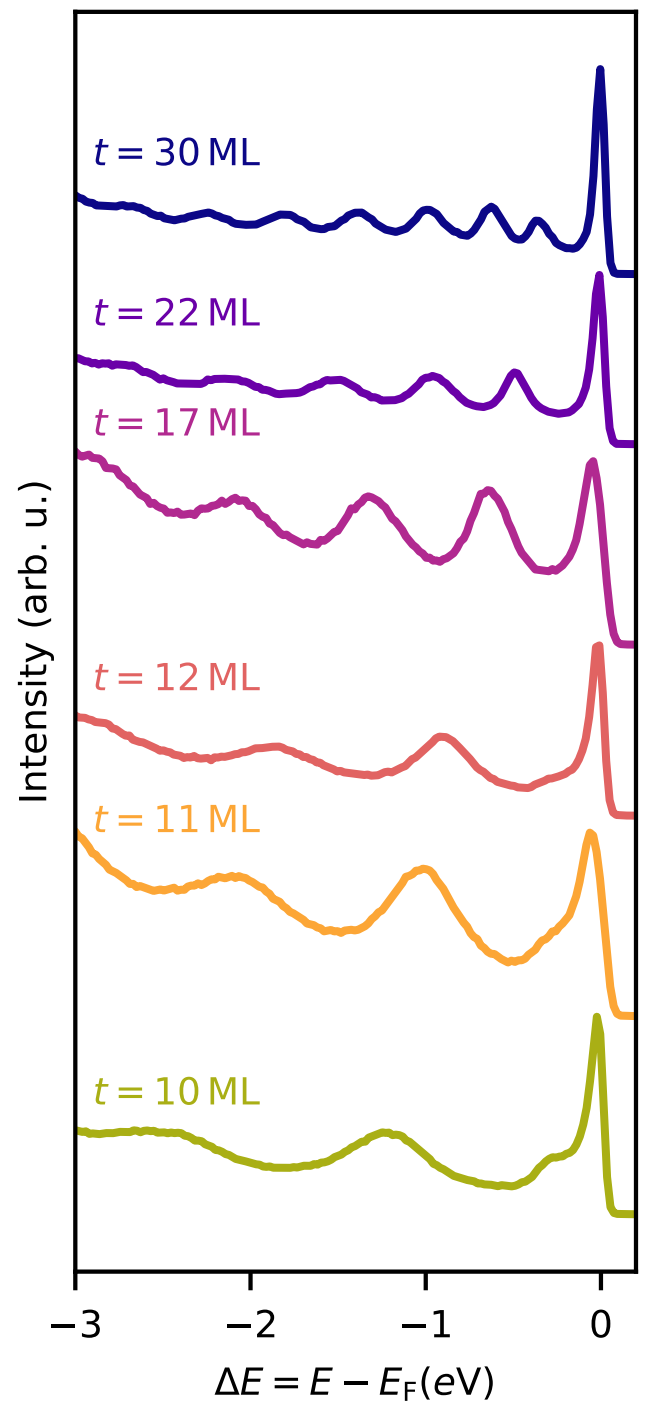

FIG. S2. **Energy distribution curves (EDC) of angle-resolved photoemission spectra** at normal emission of the freshly deposited Ag films of different monolayer (ML) thickness t for each sample studied in this work. The spectra feature a sharp peak near the Fermi energy E_F that corresponds to the Ag surface state, followed by the quantum well states at higher binding energy. Note that a lower resolution setting of the analyzer was used to measure the $t = 11$ ML and $t = 17$ ML samples, resulting in slightly broader peaks and lower measured surface state intensity due to convolution of the energy resolution with the Fermi distribution function.

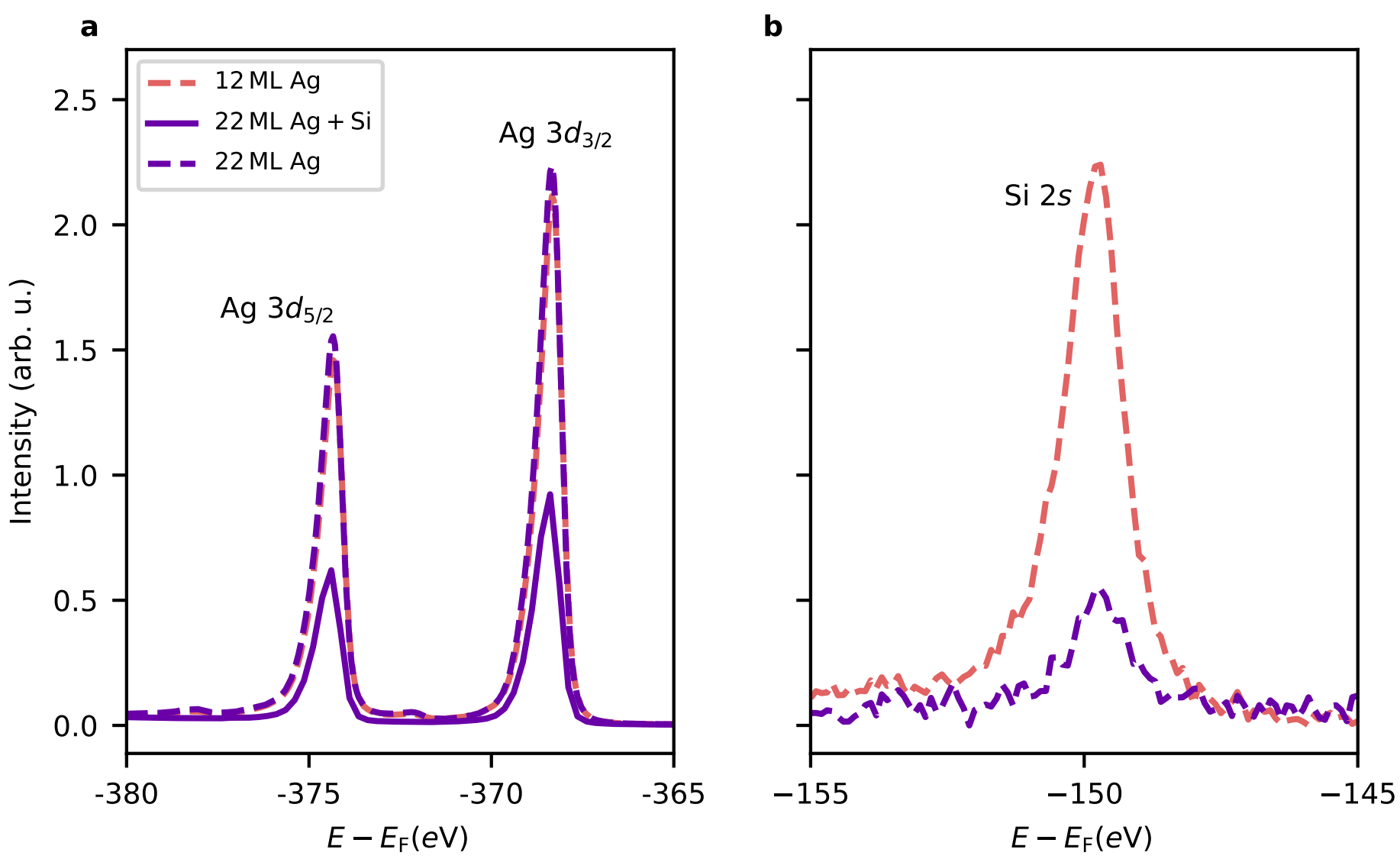

FIG. S3. **X-ray photoelectron spectra** of 12 ML (red dashed-lines) and 22 ML (blue dashed-lines) Ag film freshly grown in different sections of the same Si substrate. **a,** Ag 3d spectra showing the spin-orbit split $J = 3/2$ and $J = 5/2$ peaks. The spectrum for the 22 ML region after capping the Ag films with a ≈ 1 nm Si capping layer is also plotted as a solid blue line. **b,** Si 2s spectra collected on the two regions of Ag film thickness. Due to the surface sensitivity of the measurement, there is only a small increase in the Ag 3d peak intensities for the 22 ML Ag film in comparison with the 12 ML film in **a**, however, the greater Ag thickness corresponds to suppression of the substrate Si 2s peak in **b**. Similarly, the coverage of the Ag film with a capping Si layer is confirmed by suppression of the Ag 3d signal.

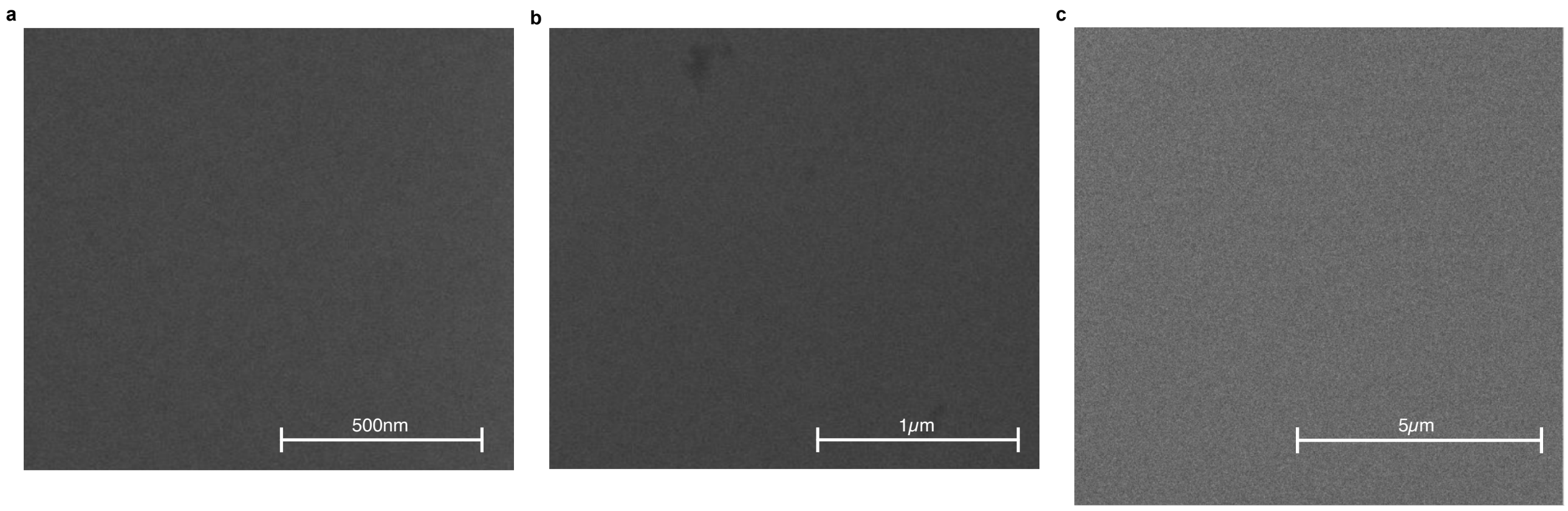

FIG. S4. **Scanning electron microscope (SEM)** images at different resolutions of the silver films covered with a capping layer. Imperfections on the sample used to focus the microscope to resolve the homogeneous surface.

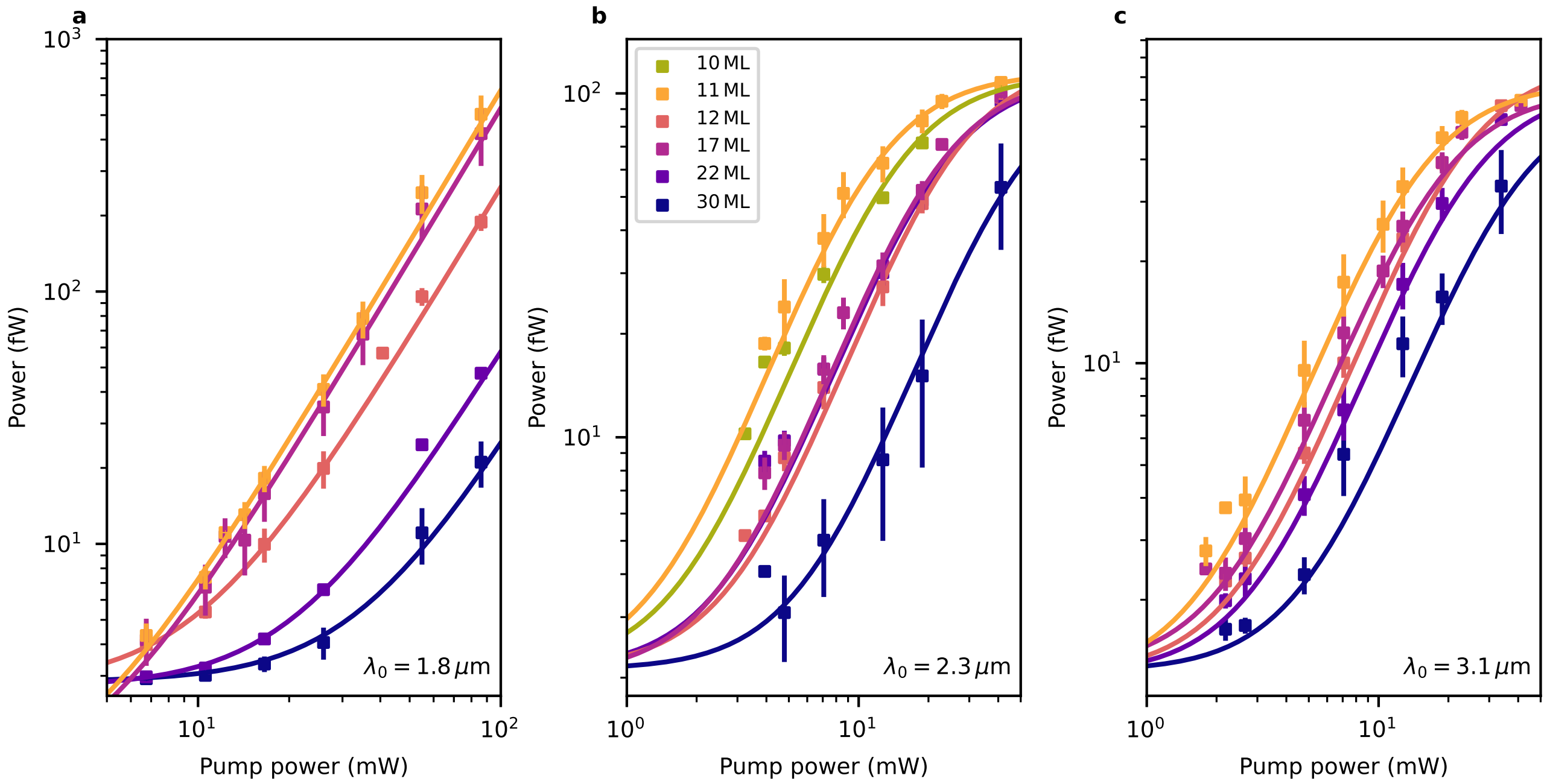

FIG. S5. **Power scaling of the measured second-harmonic signal a,** For an excitation wavelength of $\lambda_0 = 1.8\,\mu$m, **b,** $2.3\,\mu$m and **c,** $3.1\,\mu$m in crystalline silver films for different film thicknesses, supporting the Figure 4 of the main text. The data are fitted using a second-order power law. A constant background and detector saturation are taken into account for **b** and **c**. The excitation (SHG) power is measured before (after) the sample. Fresnel reflections are not taken into account. Error bars represent the standard deviation.

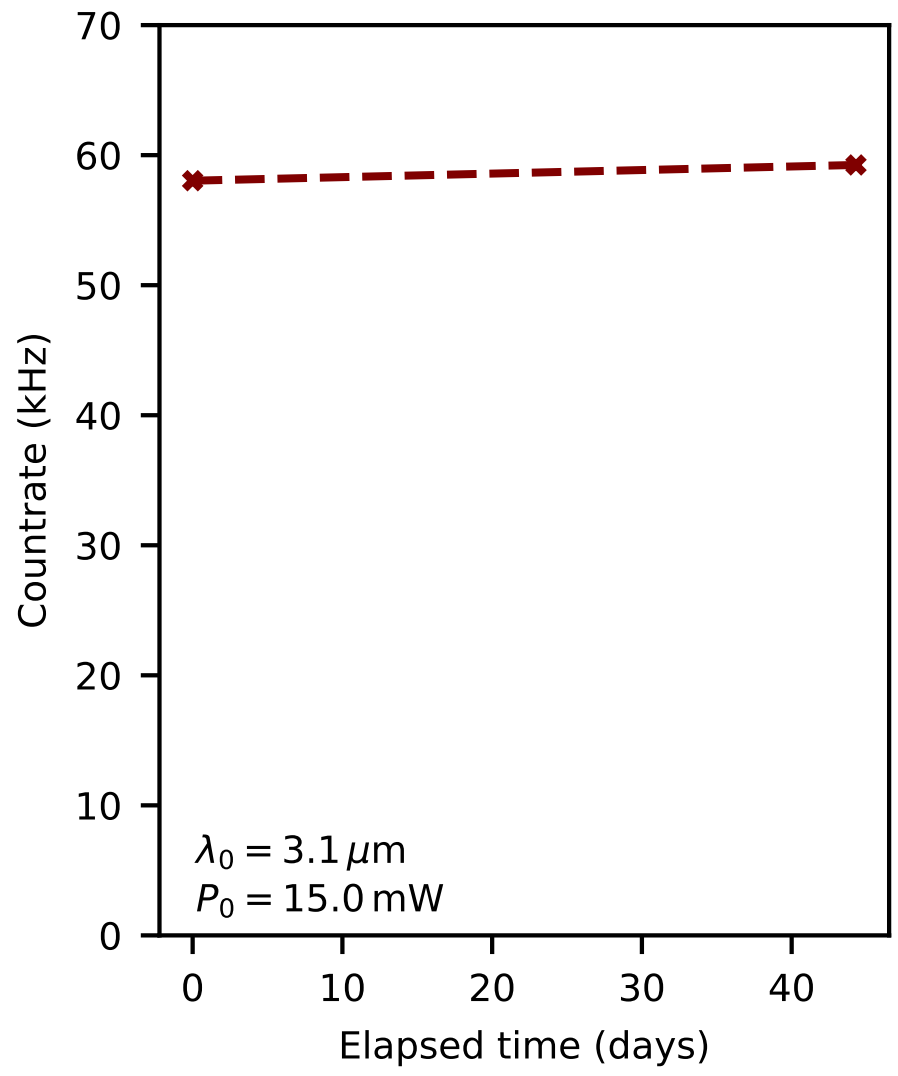

FIG. S6. **Durability of SHG count rate.** We show results acquired from a 17 ML silver film. The signal strength remained stable for over 44 days. The line serves as a guide to the eye. The excitation wavelength was $\lambda_0 = 3.1\,\mu$m. The average excitation power was $P_0 = 15\,$mW.

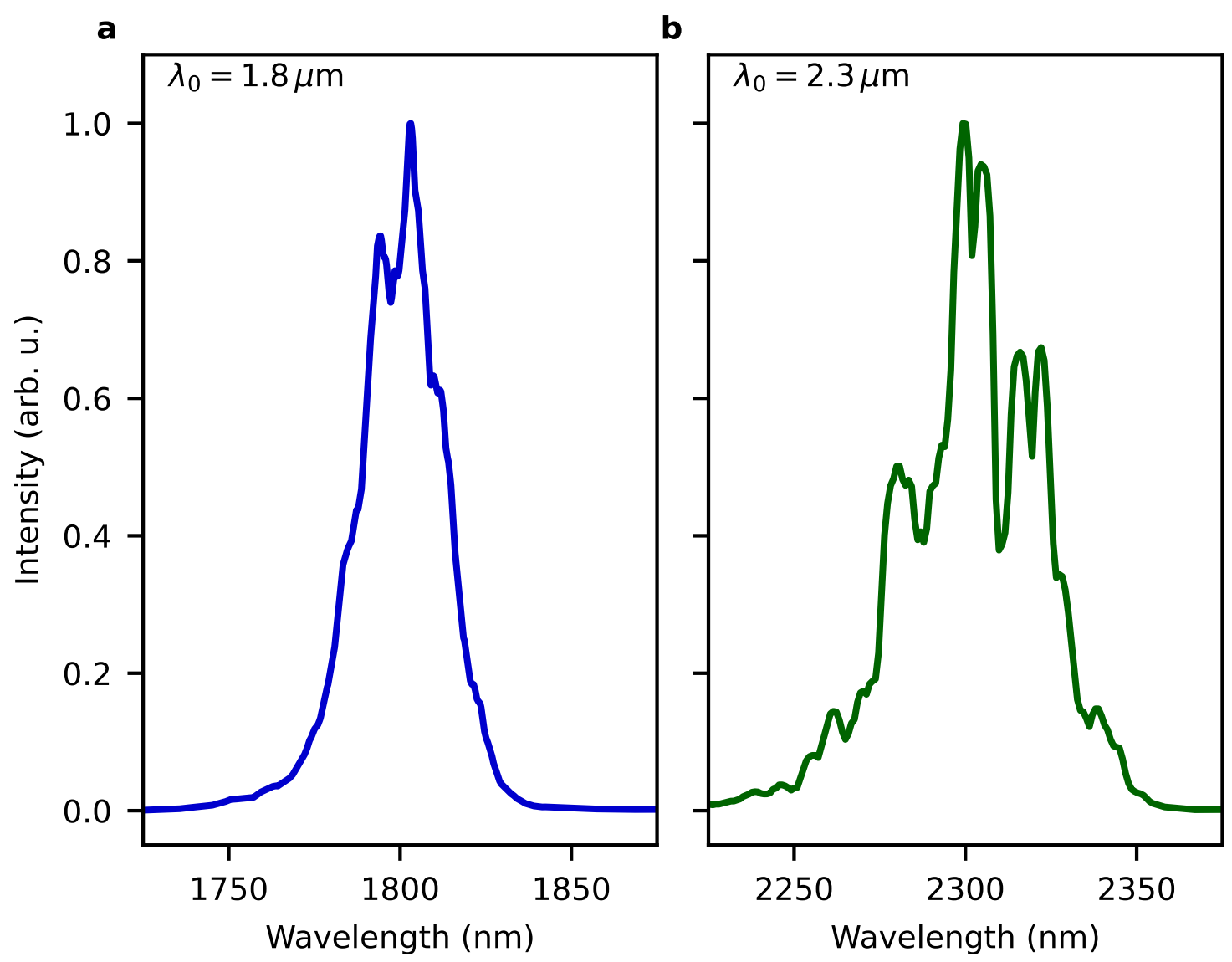

FIG. S7. **Spectra of the excitation beam a-b**, Spectra of the excitation beam at wavelengths $1.8\,\mu$m and $2.3\,\mu$m.

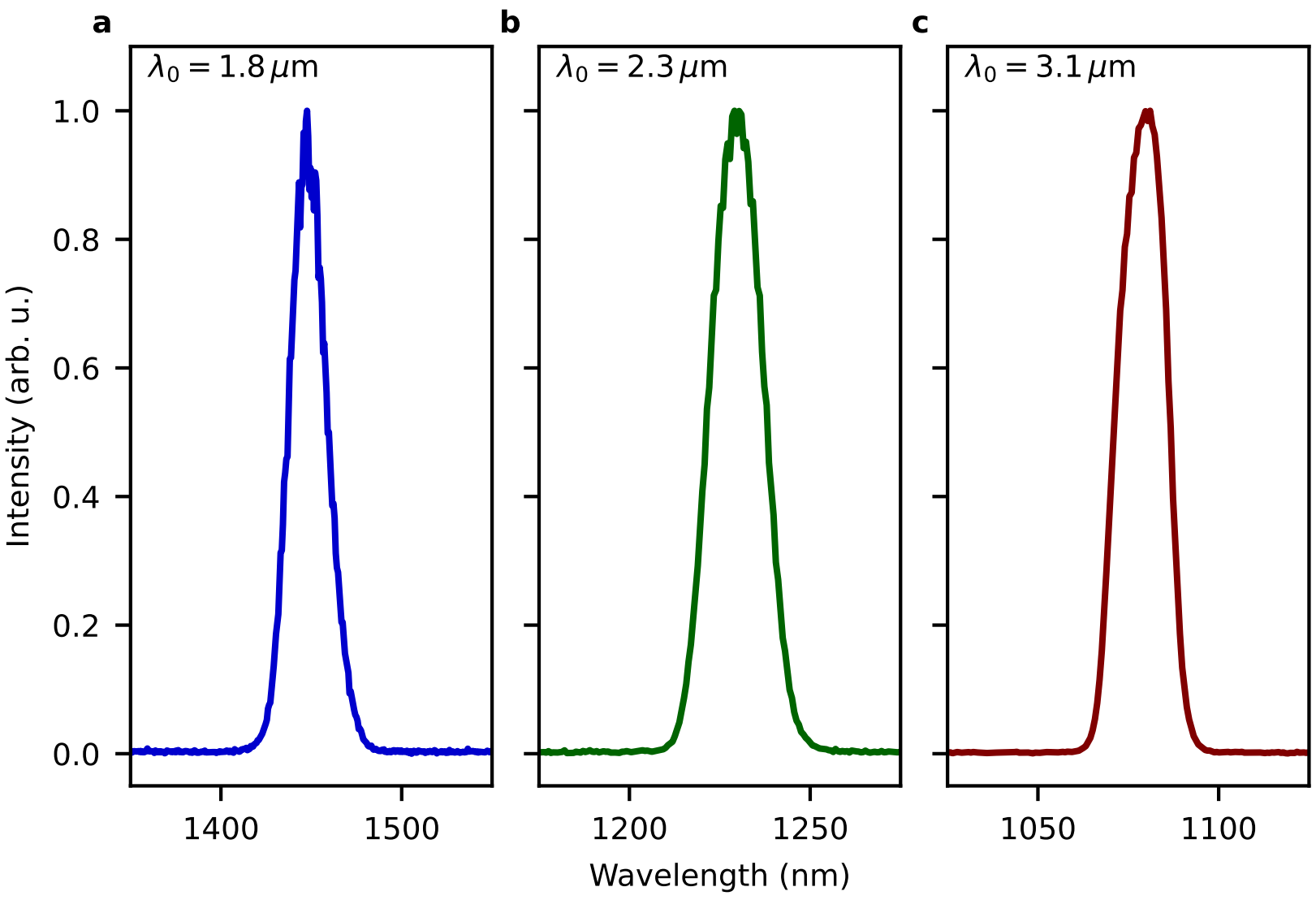

FIG. S8. **Spectra of the unused optical parametric oscillator (OPO) signal beam a-c** for the corresponding used OPO idler wavelengths 1.8 μm, 2.3 μm and 3.1 μm, respectively.

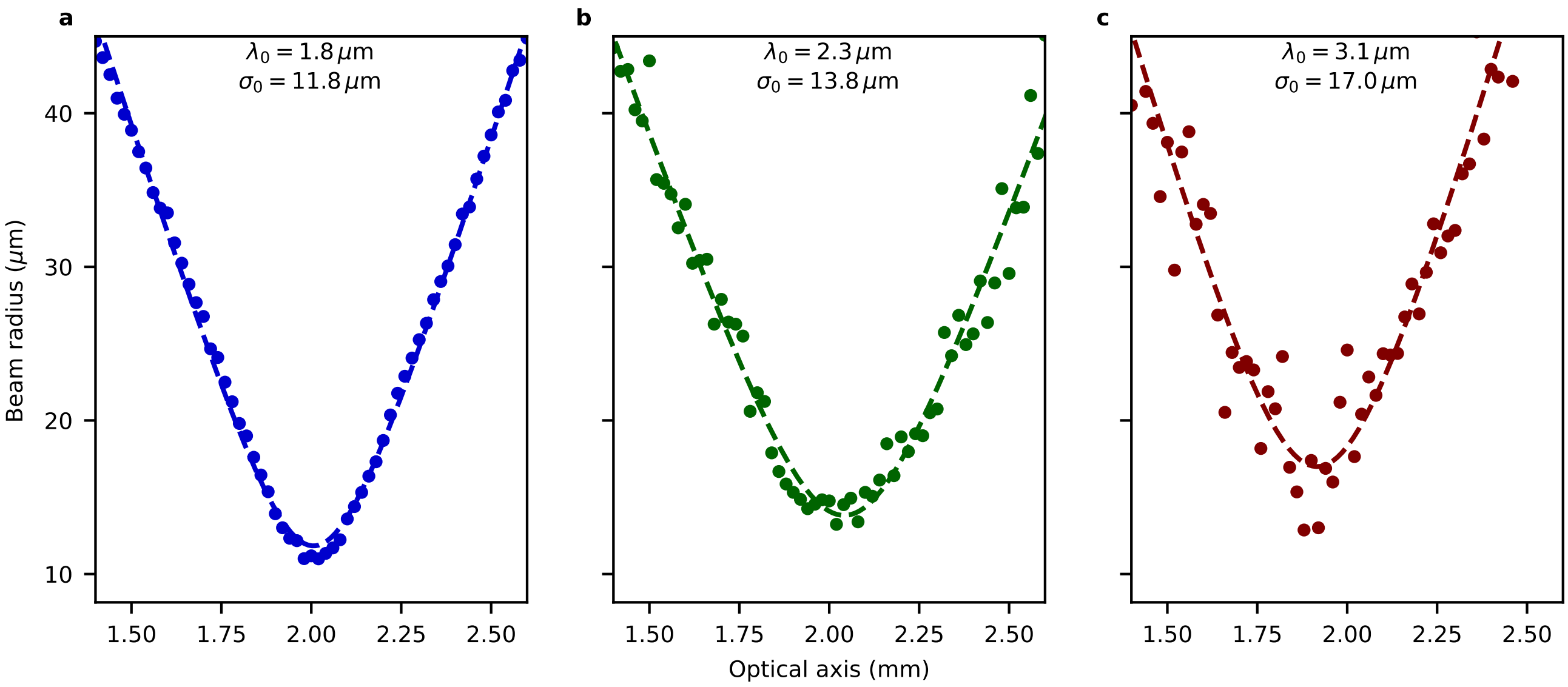

FIG. S9. **Beam waist σ_0 of the excitation beam in the focus a-c** Beam radius derived from Knife-Edge measurements for different position along the optical axis for incident wavelengths λ_0 = 1.8 μm, 2.3 μm and 3.1 μm, respectively.

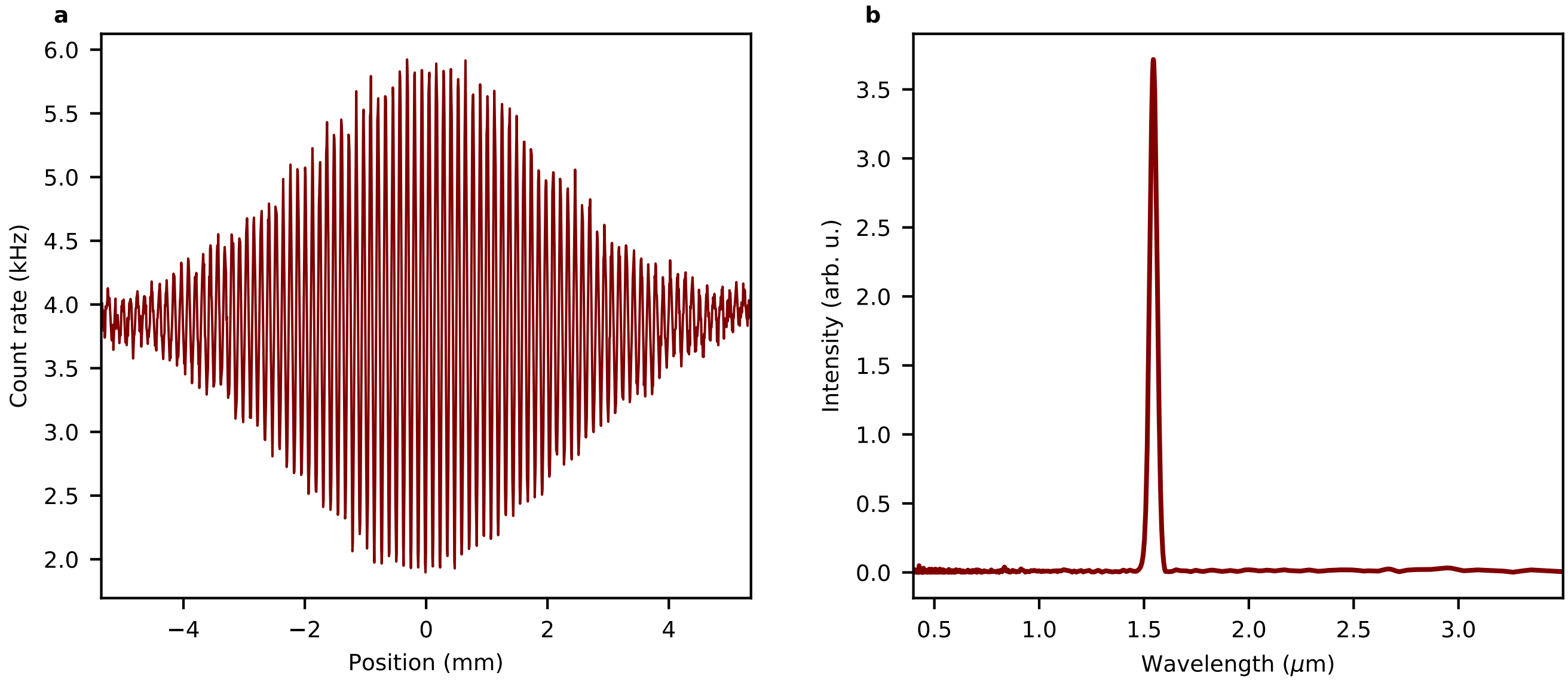

FIG. S10. **Interferometer based spectrum a,** Interferogram of an interferometric autocorrelation from the SHG signal at an excitation wavelength of 3.1 μm. **b,** Power spectral density of the Fourier transformed autocorrelation function.

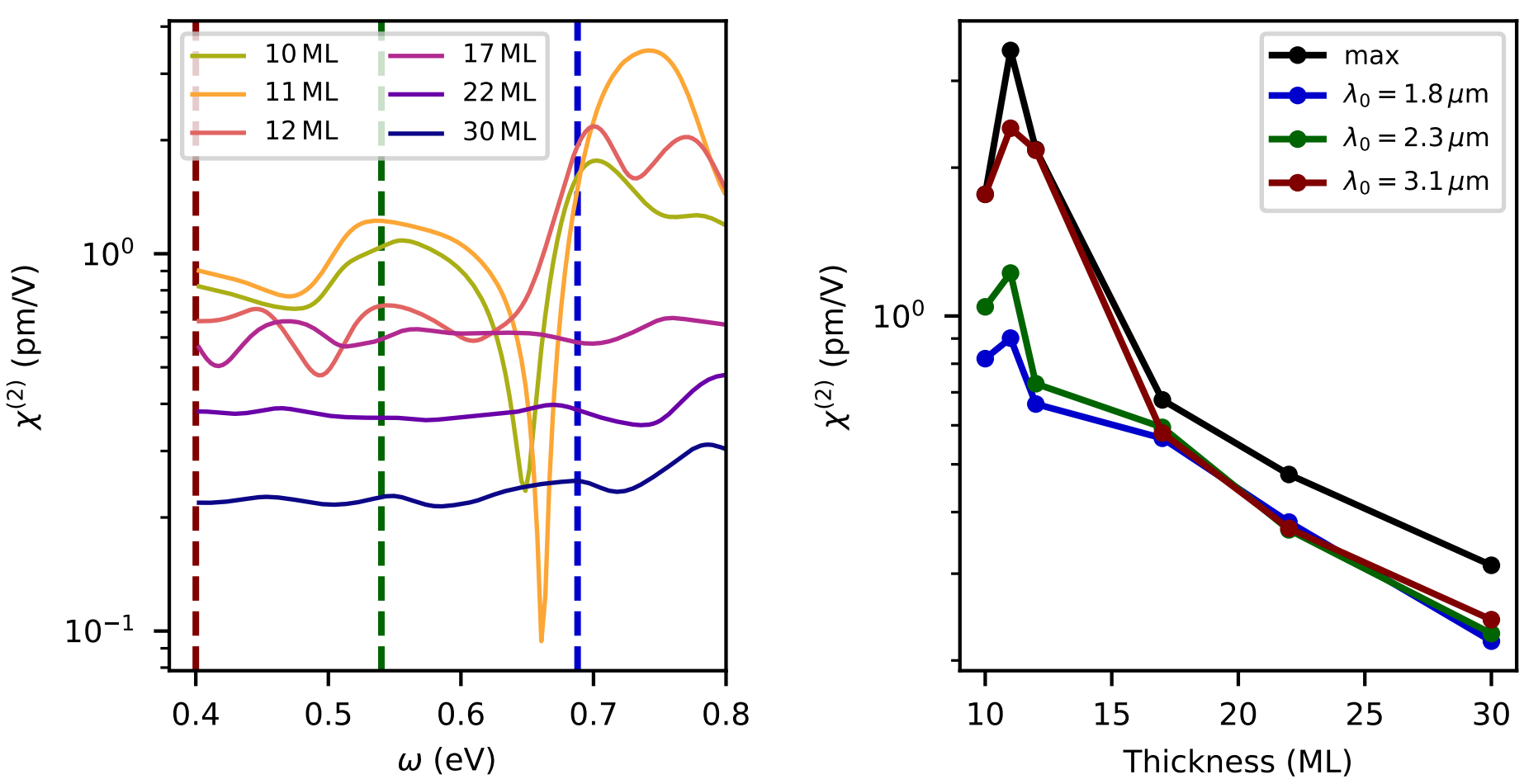

FIG. S11. **Simulated $\chi^{(2)}$ in dependence on the silver film thicknesses. a,** The vertical dashed lines at $\omega = 0.40, \mathrm{eV}, 0.54, \mathrm{eV}$, and $0.69, \mathrm{eV}$ mark the used excitation wavelengths at $\lambda_0 = 1.8, \mu\mathrm{m}, 2.3, \mu\mathrm{m}$, and $3.1, \mu\mathrm{m}$. **b,** Extracted $\chi^{(2)}$ values at the marked photon energies for different film thicknesses. The black curve represents the maximum achievable second-order nonlinearity in the energy range displayed on the left.

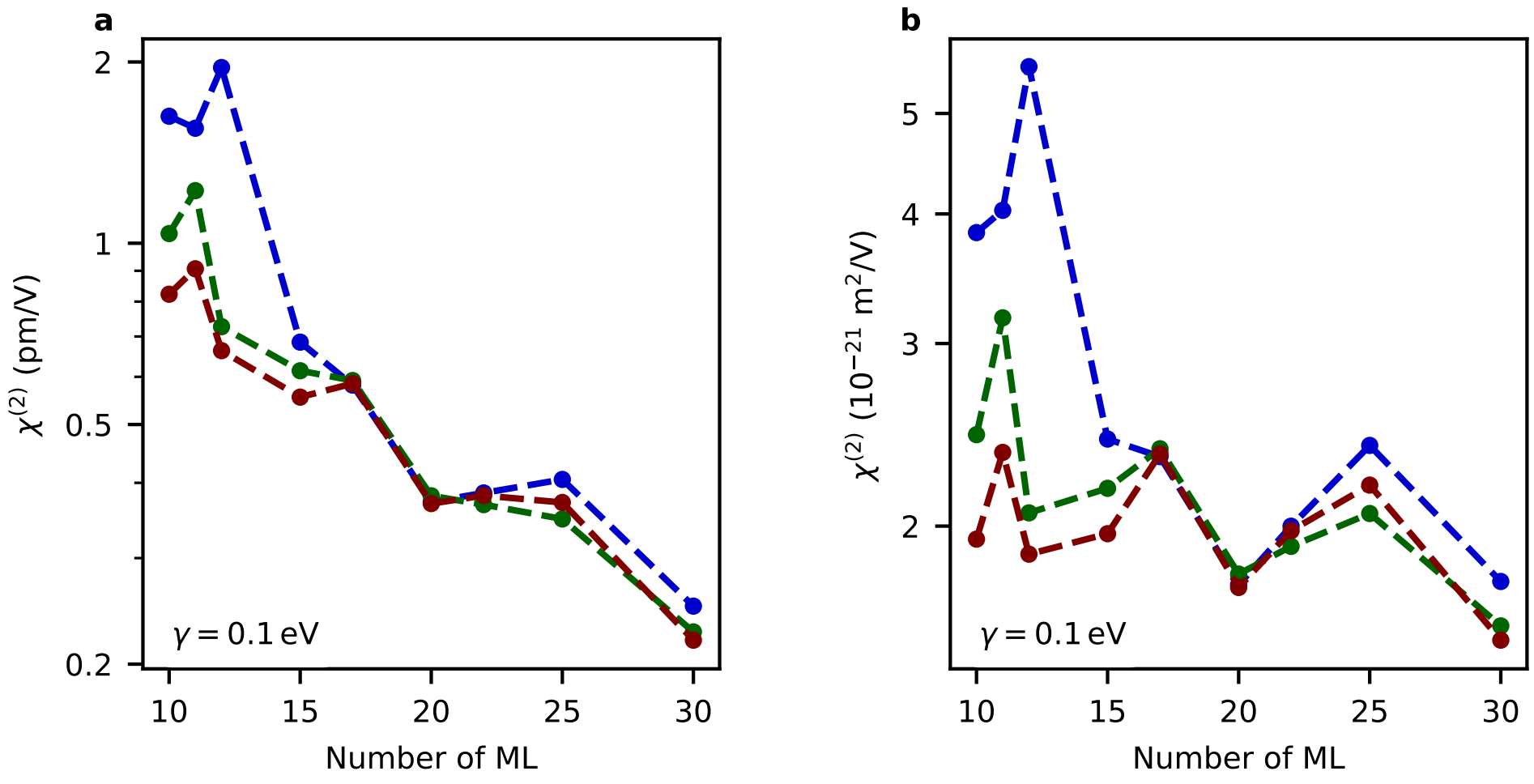

FIG. S12. **Thickness-dependent theoretical susceptibility $\chi^{(2)}$, of silver films.** Computed for incident light wavelengths of 1.8, 2.3, and 3.1 μm and a damping $\gamma = 0.1$ eV. **a**, Bulk second-order susceptibility $\chi^{(2)}$. **b**, second-order sheet susceptibility. We emphasize that the theoretical calculations in Supplementary Figure. S11 and Supplementary Figure S12 are intended to describe the experimentally relevant thickness regime of continuous crystalline Ag(111) films. They should not be interpreted as quantitative predictions for structurally discontinuous films or for thicknesses below the stability limit of the growth procedure. The simulations assume laterally homogeneous films with well-defined quantum-well states and a phenomenological damping rate. The narrow resonances predicted by the model therefore represent the intrinsic quantum-well-state contribution expected in idealized crystalline films.

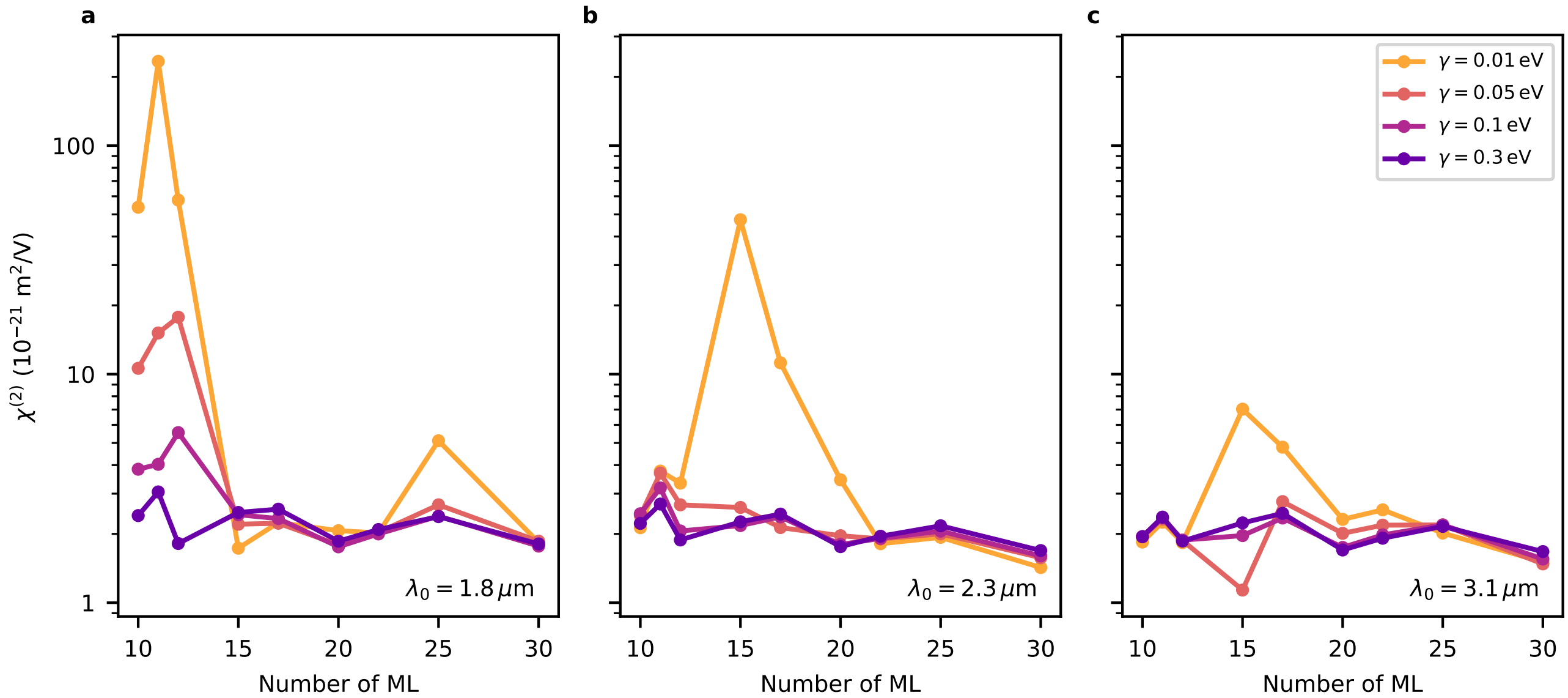

FIG. S13. **Theoretical second-order sheet susceptibility $\chi^{(2)}$.** Computed for various silver thicknesses, damping γ and incident excitation light frequencies, **a** $\lambda_0 = 1.8\,\mu$m, **b** $\lambda_0 = 2.3\,\mu$m and **c** $\lambda_0 = 3.1\,\mu$m.

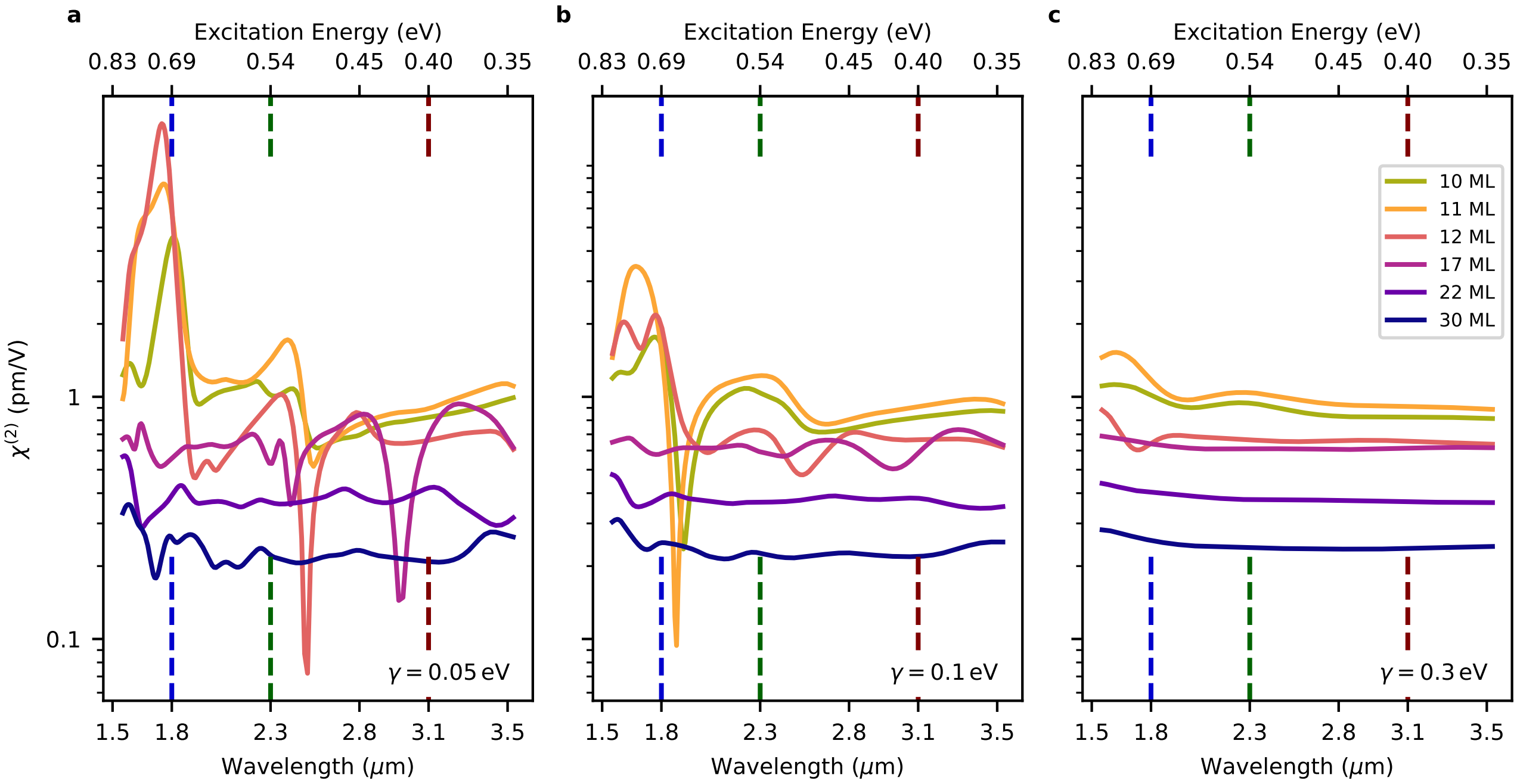

FIG. S14. **Theoretical second-order bulk susceptibility $\chi^{(2)}$.** Computed for various incident excitation light frequencies, silver thicknesses and damping **a** $\gamma = 0.05\,\mathrm{eV}$, **b** $\gamma = 0.1\,\mathrm{eV}$ and **c** $\gamma = 0.3\,\mathrm{eV}$.

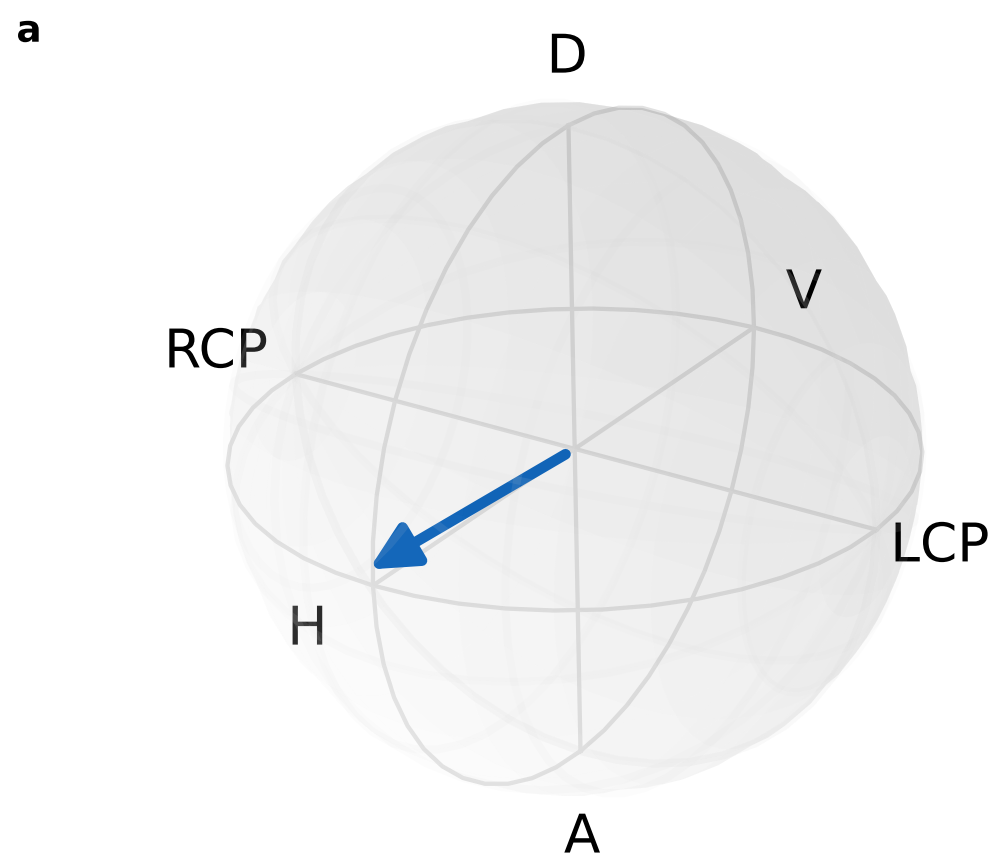

FIG. S15. **Transmission polarization-state characterization** for an excitation wavelength of $\lambda_0 = 2300\,\mathrm{nm}$ through the silicon substrate. Shown is the polarization state of the excitation beam transmitted through the silicon substrate. **a** The excitation polarization is prepared as horizontal H (p-polarized). Polarization-state tomography indicates that p-polarized light is largely preserved upon transmission, resulting in a Jones vector $J_\mathrm{p} = (0.99,\ 0.02e^{1.11j})$. **b** The excitation polarization is prepared as vertical V (s-polarized). In contrast, transmission of s-polarized light through the sample yields a Jones vector $J_\mathrm{s} = (0.59,\ 0.80e^{-2.47i})$.

I. SECOND HARMONIC RESPONSE FROM A TWO-DIMENSIONAL METALLIC SHEET: CALCULATION OF EFFICIENCIES

We consider an out-of-plane dipolar sheet excited at frequency ω by an optical beam with amplitude E_0. The two-dimensional sheet represents the few-atom-thick crystalline silver (Ag) film on top of the silicon (Si) substrate, as illustrated in Supplementary Figure S16.

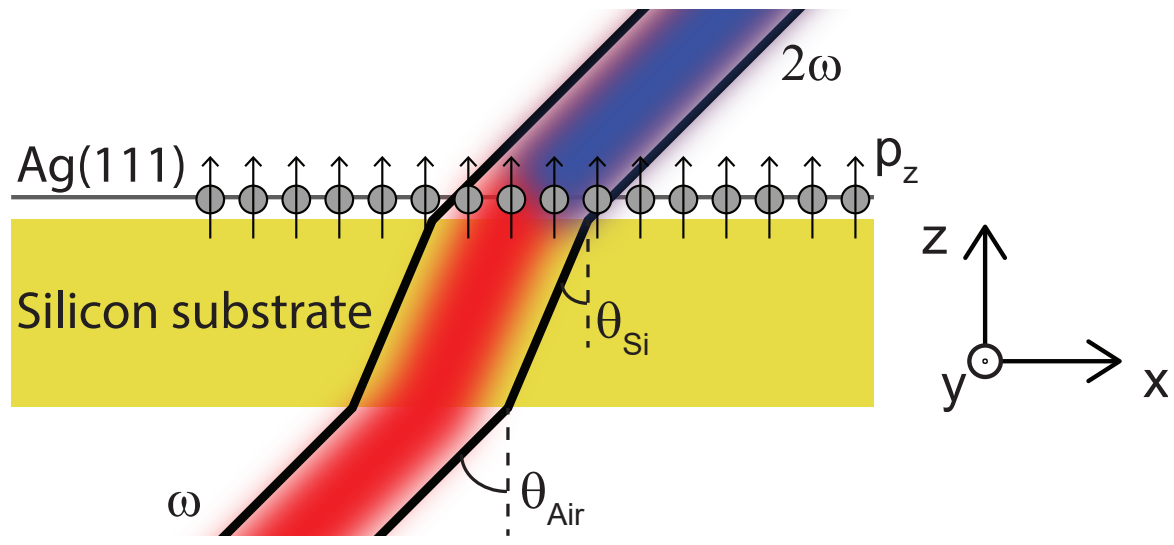

FIG. S16. A Gaussian beam impinges toward a thick silicon substrate, where a two-dimensional metallic sheet on top of it converts the light oscillating at frequency ω to 2ω (i.e., SHG). The few-atom-thick crystalline (111) silver film is featured as dipoles oscillating out-of-plane and radiating into the far field. The incident angle on the bottom silicon surface is $\theta_{\rm Air}$, which changes to $\theta_{\rm Si}$ according to Snell's law.

In particular, we consider p-polarized illumination incident from vacuum onto a Si substrate. The out-of-plane electric field component just after the Si interface (in the absence of the metal) is $E_z \approx E_1 \sin\theta_{\rm Air}$ with $E_1 = E_0\, t_p^{\rm Air\to Si}\, t_p^{\rm Si\to Air}$. Since the substrate is much thicker than the excitation wavelength, we neglect both the phase information and the Fabry-Pérot oscillations in the transmitted field amplitude. Then, the normalized field enhancement yields

$$\frac{E_z}{E_0} = t_p^{\rm Air\to Si}\, t_p^{\rm Si\to Air}\, \sin\theta_{\rm Air} \tag{S1}$$

where $\theta_{\rm Air} = \pi/4$ is the angle of incidence on the bottom air-silicon interface. The transmission coefficients read

$$t_p^{\rm Air\to Si} = \frac{2k_z\sqrt{\epsilon_{\rm Si}}}{k_z\,\epsilon_{\rm Si} + k_z'} \qquad \text{and} \qquad t_p^{\rm Si\to Air} = \frac{2k_z'\sqrt{\epsilon_{\rm Si}}}{k_z\,\epsilon_{\rm Si} + k_z'}, \tag{S2}$$

with $k_z = k\cos\theta_{\rm Air}$ and $k_z' = k\sqrt{\epsilon_{\rm Si} - \sin^2\theta_{\rm Air}}$, in which we use $\epsilon_{\rm Si} = 12$ (and $\epsilon_{\rm Air} = 1$). Supplementary Table I shows different magnitudes relevant to this problem, including the ratio $E_z/E_0 = 0.577$ found for the out-of-plane electric field driving the two-dimensional sheet of dipoles in the silver film. Note that the calculated values do not depend on the wavelength. As a result, the nonlinear dipole resulting from the second-harmonic generation process in the two-dimensional silver sheet is

$$p_z(2\omega) = \chi_s^{(2)} E_z^2(\omega) = \chi_s^{(2)} \left(\frac{E_z}{E_0}\right)^2 E_0^2, \tag{S3}$$

where we compute below how such dipoles are illuminated and how they emit into the far field, so we define the corresponding efficiencies. In particular, we compute the incident power P_0 exciting the whole system, and the resulting $P^{\rm SHG}$ after the interaction, assuming a p-polarized Gaussian beam. Moreover, we write the prescription used to obtain the values of $\chi_s^{(2)}$.

Excitation power. The incident electric field has a Gaussian profile $\mathbf{E}_0\, {\rm e}^{-\tilde{R}^2/2\sigma_0^2}$ that is carried along its propagation through the sample, including refraction inside the silicon film. In this expression, $\tilde{\mathbf{R}}$ denotes 2D coordinates in a plane perpendicular to the incident beam (i.e., tilted with respect to the sample plane, for which we use coordinates $\mathbf{R}$ without a tilde). The incident power (before arriving at the sample) is thus given by

$$P_0 = \frac{c}{2\pi}\int d^2\tilde{\mathbf{R}}\,\left|\mathbf{E}_0\, {\rm e}^{-\tilde{R}^2/2\sigma_0^2}\right|^2 = \pi\sigma_0^2\, I_0, \tag{S4}$$

where $I_0 = cE_0^2/2\pi$ is the peak light intensity at the center of the Gaussian profile.

Power of the generated second harmonic signal. The electric field produced by a two-dimensional sheet of SHG dipoles [i.e., a dipole density per unit area $\mathbf{p}(\mathbf{R})$ oscillating at frequency 2ω] at the metallic layer

TABLE S1. Values of the transmitted electric field through the silicon substrate ($\epsilon_{\rm Si} = 12$) in the out-of-plane direction as well as the ratio of incident intensity exciting the silver film.

Quantity	Expression	Numerical value
Angle in Air	$\theta_{\rm Air}$	$\pi/4$ rad $= 45°$
Angle in Silicon	$\theta_{\rm Si}$	0.2056 rad $= 11.7782°$
p-polarized reflection (Air $\to$ Si)	$r_p^{\rm Air\to Si}$	0.4289
p-polarized transmission (Air $\to$ Si)	$t_p^{\rm Air\to Si}$	0.4125
p-polarized transmission (Si $\to$ Air)	$t_p^{\rm Si\to Air}$	1.9783
Angle factor	$\sin\theta_{\rm Air} = \sin(\pi/4)$	0.7071
Product of transmission coefficients	$t_p^{\rm Air\to Si} t_p^{\rm Si\to Air}$	0.8160
Field enhancement	$\dfrac{E_z}{E_0} = t_p^{\rm Air\to Si}\, t_p^{\rm Si\to Air}\, \sin\theta_{\rm Air}$	0.5770
Intensity enhancement	$\left\lvert\dfrac{E_z}{E_0}\right\rvert^2$	0.3329

plane ($z = 0$) reads

$$\mathbf{E}^{\rm SHG}(\mathbf{r}) = \int d^2\mathbf{R}' \left[(2k)^2 + \nabla\otimes\nabla\right] \frac{{\rm e}^{{\rm i}2k|\mathbf{r}-\mathbf{R}'|}}{|\mathbf{r}-\mathbf{R}'|}\mathbf{p}(\mathbf{R}') \tag{S5}$$

$$= \int d^2\mathbf{R}'(2k)^2 \int \frac{d^2\mathbf{Q}}{(2\pi)^2}\frac{2\pi{\rm i}}{k'_z}\left[(\hat{\epsilon}_{\rm s}\cdot\mathbf{p})\hat{\epsilon}_{\rm s} + (\hat{\epsilon}_{\rm p}^{\pm}\cdot\mathbf{p})\hat{\epsilon}_{\rm p}^{\pm}\right]{\rm e}^{{\rm i}\mathbf{Q}\cdot(\mathbf{R}-\mathbf{R}')\pm{\rm i}k'_z z}, \tag{S6}$$

with $\hat{\epsilon}_{\rm s} = (-Q_y\hat{\mathbf{x}} + Q_x\hat{\mathbf{y}})/Q$ and $\hat{\epsilon}_{\rm p}^{\pm} = (\pm k'_z\mathbf{Q} - Q^2\hat{\mathbf{z}})/(2kQ)$, where $k'_z = \sqrt{(2k)^2 - Q^2}$, $\mathbf{Q} = (Q_x, Q_y)$, and $k = \omega/c$ the wavenumber at the fundamental frequency. Here, we have used Weyl's identity,

$$\frac{{\rm e}^{{\rm i}kr}}{r} = \frac{\rm i}{2\pi}\int\frac{d^2\mathbf{Q}}{k_z}{\rm e}^{{\rm i}\mathbf{Q}\cdot\mathbf{R}}{\rm e}^{\pm{\rm i}k_z z}. \tag{S7}$$

For excitation by the Gaussian profile defined by the beam waist σ_0 in air (within a plane perpendicular to the incident propagation direction), the illuminated region on the interface plane corresponds to its elliptical projection. Denoting $\theta_{\rm Air}$ the angle of incidence in air, such an elliptical projection has $1/e$ Gaussian intensity radii

$$\sigma_\perp = \sigma_0 \qquad \text{and} \qquad \sigma_\parallel = \frac{\sigma_0}{\cos\theta_{\rm Air}}, \tag{S8}$$

where $\parallel$ and $\perp$ denote directions parallel and perpendicular to the plane of incidence, respectively (see Supplementary Figure S16). The field envelope on the metal layer is proportional to $E(\mathbf{R}) \propto \exp[-x^2/(2\sigma_\parallel^2) - y^2/(2\sigma_\perp^2)]$, where $k_\parallel = k\sin\theta_{\rm Air}$ is the in-plane incident light wave vector, so that the induced dipole sheet profile $|\mathbf{p}(\mathbf{R})| \propto E^2$ can be written as $\mathbf{p}(\mathbf{R}) = p_z\,{\rm e}^{{\rm i}2k_\parallel x}\exp[-x^2/\sigma_\parallel^2 - y^2/\sigma_\perp^2]\hat{\mathbf{z}}$ (retaining only the z component dominating the SHG response). Inside Eq. (S6), we identify the Fourier transform

$$\mathbf{p}(\mathbf{Q}) = p_z\hat{\mathbf{z}}\int d^2\mathbf{R}'\,{\rm e}^{-{\rm i}\mathbf{Q}\cdot\mathbf{R}'}{\rm e}^{{\rm i}2k_\parallel x'}{\rm e}^{-x'^2/\sigma_\parallel^2 - y'^2/\sigma_\perp^2} = \pi\,\sigma_\parallel\sigma_\perp\,p_z\hat{\mathbf{z}}\,\exp\Big\{-\left[(Q_x - 2k_\parallel)^2\sigma_\parallel^2 + Q_y^2\sigma_\perp^2\right]/4\Big\}, \tag{S9}$$

and therefore, the electric field produced by the sheet of dipoles (at $z > 0$) is

$$\mathbf{E}^{\rm SHG}(\mathbf{r}) = \int\frac{d^2\mathbf{Q}}{(2\pi)^2}\frac{2\pi{\rm i}}{k'_z}\left[-2kQ\,p_z\left(1 + r_p^{\rm Air\to Si}\right)\pi\sigma_\parallel\sigma_\perp\,\exp\Big\{-\left[(Q_x - 2k_\parallel)^2\sigma_\parallel^2 + Q_y^2\sigma_\perp^2\right]/4\Big\}\,\hat{\epsilon}_{\rm p}^{+}\right]{\rm e}^{{\rm i}\mathbf{Q}\cdot\mathbf{R}+{\rm i}k'_z z}. \tag{S10}$$

Using the stationary-phase approximation in the above integral [1], which has the form

$$\lim_{kr\to\infty}\int\frac{d^2\mathbf{Q}}{(2\pi)^2}\frac{2\pi{\rm i}}{k'_z}g(\mathbf{Q}){\rm e}^{{\rm i}(\mathbf{Q}\cdot\mathbf{R}+k'_z z)} = \frac{{\rm e}^{2{\rm i}kr}}{r}g(2k\mathbf{R}/r), \tag{S11}$$

the far electric field becomes

$$\mathbf{E}^{\rm SHG}(\mathbf{r}) = \frac{{\rm e}^{{\rm i}2kr}}{r}\left\{-2kQ\,p_z(1 + r_p^{\rm Air\to Si})\,\pi\sigma_\parallel\sigma_\perp\,\exp\Big\{-\left[(Q_x - 2k_\parallel)^2\sigma_\parallel^2 + Q_y^2\sigma_\perp^2\right]/4\Big\}\,\hat{\epsilon}_{\rm p}^{+}\right\}, \tag{S12}$$

where $\mathbf{Q} = 2k\mathbf{R}/r$. The emitted SHG power is calculated by integrating the Poynting vector crossing an upper hemisphere of large radius as

$$P^{\rm SHG} = \frac{c}{2\pi}\int_{\cos\theta>0} d^2\Omega\,(2k)^2|p_z|^2\left|1+r_p^{\rm Air\to Si}\right|^2 \exp\Big\{-\Big[(Q_x-2k_\parallel)^2\sigma_\parallel^2+Q_y^2\sigma_\perp^2\Big]/2\Big\}\,\left(\pi\sigma_\parallel\sigma_\perp\right)^2 Q^2. \tag{S13}$$

To perform this integral, we change variables to $\mathbf{Q}$ space, using $d^2\mathbf{Q} = (2k)^2\cos\theta\, d^2\Omega$, which directly yields

$$P^{\rm SHG} = \frac{c}{2\pi}\int \frac{d^2\mathbf{Q}}{\cos\theta}\,|p_z|^2\left|1+r_p^{\rm Air\to Si}\right|^2 \exp\Big\{-\Big[(Q_x-2k_\parallel)^2\sigma_\parallel^2+Q_y^2\sigma_\perp^2\Big]/2\Big\}\,\left(\pi\sigma_\parallel\sigma_\perp\right)^2 Q^2. \tag{S14}$$

Assuming that the SHG emission is mainly concentrated around the observation angle $\theta_{\rm Air}$ in the far field, and that the Gaussian factor suppresses contributions outside this region, we approximate

$$P^{\rm SHG} \approx \frac{2\pi\,\omega^2\,\sigma_\parallel^2\sigma_\perp^2}{c}\,\frac{\sin^2\theta_{\rm Air}\,|p_z|^2\left|1+r_p^{\rm Air\to Si}\right|^2}{\cos\theta_{\rm Air}}\int d^2\mathbf{Q}\,\exp\Big\{-\Big[(Q_x-2k_\parallel)^2\sigma_\parallel^2+Q_y^2\sigma_\perp^2\Big]/2\Big\}, \tag{S15}$$

where we have approximated $\theta \approx \theta_{\rm Air}$ because $k\sigma_0 \gg 1$, so we take non-exponential factors outside the integral, including $Q^2 \approx (2k_\parallel)^2 = (2k)^2\sin^2\theta_{\rm Air}$. The $\mathbf{Q}$ integral can be directly carried out, yielding

$$P^{\rm SHG} = \frac{4\pi^2\,\omega^2\,\sigma_\parallel\sigma_\perp}{c}\,\frac{\sin^2\theta_{\rm Air}}{\cos\theta_{\rm Air}}\,|p_z|^2\left|1+r_p^{\rm Air\to Si}\right|^2. \tag{S16}$$

Using the definition of $p_z(2\omega)$ in Eq. (S3) and $I_0 = cE_0^2/2\pi$, we obtain the emitted SHG power

$$P^{\rm SHG} = I_0^2\,\frac{16\pi^4\omega^2\,\sigma_0^2}{c^3}\,\tan^2\theta_{\rm Air}\left|\chi_s^{(2)}\left(\frac{E_z}{E_0}\right)^2\right|^2\left|1+r_p^{\rm Air\to Si}\right|^2, \tag{S17}$$

where we express the result in terms of the incident Gaussian width in air σ_0 [Eqs. (S8)].

Efficiencies in the second harmonic generation. From the impinging power [Eq. (S3)] and the generated second harmonic power Eq. (S17), we define the Gaussian-profile peak power efficiency as

$$\eta' \equiv \frac{P^{\rm SHG}}{P_0^2} = \frac{16\pi^2\,\omega^2}{\sigma_0^2\,c^3}\,\tan^2\theta_{\rm Air}\left|\chi_s^{(2)}\left(\frac{E_z}{E_0}\right)^2\right|^2\left|1+r_p^{\rm Air\to Si}\right|^2. \tag{S18}$$

Therefore, from the measured efficiencies, we obtain the second-harmonic surface susceptibility as

$$\left|\chi_s^{(2)}\right| = \frac{|E_0/E_z|^2}{\left|1+r_p^{\rm Air\to Si}\right|}\sqrt{\frac{\sigma_0^2\,c^3}{16\pi^2\,\omega^2}\,\frac{\eta'}{\tan^2\theta_{\rm Air}}}\qquad \text{(Gaussian units)}, \tag{S19}$$

as well as the effective bulk susceptibility from

$$\left|\chi_b^{(2)}\right| = \frac{1}{t}\left|\chi_s^{(2)}\right|, \tag{S20}$$

in which we divide by the corresponding thickness $t = Na_s$ of an N-monoatomic layers in the silver film with $a_s = 0.2361$ nm interlayer spacing. We show the unit conversion rules in Supplementary Table I across SI, esu, and atomic units used to compare our results with other values in the literature. For the sake of completeness, to obtain the ratio between SHG and fundamental intensities $\eta = I^{\rm SHG}/I_0^2$ in terms of the ratio of powers $\eta' = P^{\rm SHG}/P_0^2$ in Eq. (S18), we consider the relations $P^{\rm SHG} = A_{2\omega}I^{\rm SHG}$ and $P_0 = A_\omega I_0$, which involve the corresponding surface-projected areas. The latter satisfy the relation $A_\omega/A_{2\omega} = 2$. Using this together with $A_\omega = \pi\sigma_0^2/\cos\theta_{\rm Air}$, we find $\eta' = (\eta/2\pi\sigma_0^2)\cos\theta_{\rm Air}$, so Eq. (S19) becomes

$$\left|\chi_b^{(2)}\right| = \frac{1}{4\pi\,k\tan\theta_{\rm Air}}\,\frac{|E_0/E_z|^2}{\left|1+r_p^{\rm Air\to Si}\right|}\sqrt{\frac{c\,\eta\,\cos\theta_{\rm Air}}{2\pi}}\qquad \text{(Gaussian units)}, \tag{S21}$$

written in the main text as a function of the peak intensity efficiency η. Finally, average powers are converted to peak powers through $P_{\rm peak} = (f_{\rm shape}/f_{\rm rep}\tau)P_{\rm ave}$, and analogously for the corresponding intensities. The laser operates at a repetition rate $f_{\rm rep} = 76\times10^6$ Hz with a pulse duration $\tau = 200\times10^{-15}$ s and a pulse-shape factor $f_{\rm shape} = 0.94$. For the experimental parameters used in this work, the conversion factor from average to peak power is $f_{\rm shape}/(f_{\rm rep}\tau) = 6.18\times10^4$.

Several approximations are made in the above derivation. As described in the main text, the silver film is encapsulated between silicon and silica in order to preserve the quality of the silver surface. However, most of

the light incident on the capping layer is transmitted, with $t_p^{\mathrm{Air}\to\mathrm{SiO_2}} t_p^{\mathrm{SiO_2}\to\mathrm{Air}} \approx 0.95$ using $\epsilon_{\mathrm{SiO_2}} = 4$, and we therefore neglect the effect of the capping layer. In addition, the finite thickness of the silver film attenuates both the fundamental field and the generated SHG signal. For the thicknesses considered here, we estimate this correction as $|\int_0^t dz\, \mathrm{e}^{\mathrm{i}k_z z}/t|^2/|\int_0^t dz\, \mathrm{e}^{\mathrm{i}k'_z z}/t| \approx 1$, and therefore neglect it. Finally, the linear response of the film at the SHG frequency introduces a correction proportional to $(t/\lambda)\chi^{(1)}E_z(2\omega)$, which we also estimate to be negligible. The values obtained above are valid for the thicknesses and wavelengths considered in this work. We note, however, that the estimates of these corrections are based on measured optical constants for silver [2], and their precise magnitudes may therefore depend on the optical data set used.

TABLE S2. Conversion table for surface $\chi^{(2)}$ between atomic units (a.u.), esu (cm^2/statV), SI (m^2/V). We use the electron charge e, the Bohr radius a_0 and the Hartree energy E_h in their corresponding units.

**From / To**	atomic units (a.u.)	esu (cm^2/statV)	SI (m^2/V)
atomic units (a.u.)	1	$e^{(\mathrm{esu})}a_0^2/E_h^{(\mathrm{esu})} \approx 3.08 \times 10^{-16}$	$4\pi e^{(\mathrm{SI})}a_0^2/E_h^{(\mathrm{SI})} \approx 1.29 \times 10^{-21}$
esu (cm^2/statV)	–	1	$4\pi/(3 \times 10^6) \approx 4.19 \times 10^{-6}$
SI (m^2/V)	–	–	1

II. SECOND HARMONIC GENERATION IN SILVER: LITERATURE REVIEW

SHG efficiencies. Table S3 compares reported peak SHG intensity conversion efficiencies η from ultra-thin silver films across different thicknesses, wavelengths, and experimental geometries. Early measurements on bulk-like Ag surfaces showed extremely weak SHG responses, while later studies on thin films consistently reported substantially enhanced efficiencies, highlighting the dominant role of surface and interface contributions once inversion symmetry is broken. Most previous works employed reflection geometries with p-polarized excitation at oblique incidence and often reported relative or arbitrary units, complicating direct quantitative comparison. Within this context, the efficiencies measured in the present work fall within the range of previously reported values, despite the use of a transmission geometry and longer excitation wavelengths. Additionally, a few more studies show conversion efficiencies for silver in system that are not directly comparable to our work [3–5], where they look into particles, cavities, or polycrystalline silver.

Absolute values. Table S4 presents a unified compilation of reported second-order surface nonlinear susceptibilities $\chi^{(2)}_{ijk}$ for silver interfaces and thin films, expressed consistently in SI surface units (m^2/V). Values originally reported in Gaussian or esu units (cm^2/statvolt) were converted using the factor $4\pi/(3 \times 10^6)$, as summarized in Table I. The compilation includes: (i) dipolar surface tensor components for Ag(111) measured by Wong and Richmond [6]; (ii) isotropic Ag thin-film surface nonlinearities reported by Krause et al. [11], including an effective surface susceptibility arising from integrated bulk quadrupolar currents; (iii) Ag–air interface nonlinearities measured by O'Donnell and Torre [12]; and (iv) Ag–air surface nonlinearities inferred by Chang and Lue [7] from angular-resolved SHG measurements fitted using the hydrodynamic Rudnick–Stern model with fitted surface parameter $a = 7$ (and $b = -1$). The listed tensor elements comprise the normal dipolar component $\chi^{(2)}_{zzz}$, mixed dipolar components $\chi^{(2)}_{xzx}$ and $\chi^{(2)}_{zxx}$, the in-plane anisotropic dipolar term $\chi^{(2)}_{xxx}$, and, when available, the effective bulk-derived surface contribution $\chi^{(2)}_q$. High order values can also be achieved in silver nanoparticles [13, 14], however, the efficiency of the transformation is enhanced provided plasmonic resonances.

TABLE S3. **Extracted conversion efficiencies η for second harmonic generation in ultra-thin crystalline silver films for various works.** We list the values of the SHG conversion efficiency η in the literature compare to our work.

Thickness (ML)	λ (μm)	Polarization	Incident angle	Method	η (m^2/W)	Ref.
no clear	0.53 to 1.53	p to p	various	reflection	$\sim 10^{-30}$	[6] 1993 - Wong et al.
1 nm to 180 nm	1.06	p to p	various	reflection	$\sim 10^{-24}$	[7] 1997 - Chang et al.
0 to 25	0.53 to 1.06	p to p	60 °	reflection	$\sim 10^{-25}$	[3] 1999 - Pedersen et al.
0 to 11	0.89 to 1.03	p to p	45 °	reflection	arb. u.	[8] 2001 - Hirayama et al.
0 to > 30	0.70 to 1.00	p to p	40 °	reflection	arb. u.	[9] 2004 - Pedersen et al.
0 to 45	0.36 to 0.47	p to p, p to s	60 °	reflection	arb. u.	[10] 2006 - Pedersen et al.
10 to 30	1.8 to 3.1	p to p	12 °	transmission	$\sim 10^{-24}$	this work

TABLE S4. **Surface second-order nonlinear susceptibilities $\chi^{(2)}$ for Ag interfaces.** We gather different values in the literature for the surface second-order susceptibility, where the x and y are the parallel components and z the perpendicular. We also added the quadrupole term $\chi^{(2)}_{\mathrm{q}}$. Dashes indicate tensor components not reported in the corresponding reference.

λ (μm)	$\chi^{(2)}_{xxx}$ (m^2/V)	$\chi^{(2)}_{zxx}$ (m^2/V)	$\chi^{(2)}_{xzx}$ (m^2/V)	$\chi^{(2)}_{zzz}$ (m^2/V)	$\chi^{(2)}_{\mathrm{q}}$ (m^2/V)	Ref.	Comments
1.530	1.89×10^{-19}	–	–	1.79×10^{-17}	–	[6] 1993 – Wong et al.	Ag-bulk
1.064	1.55×10^{-19}	5.03×10^{-20}	6.03×10^{-19}	9.63×10^{-18}	–	[6] 1993 – Wong et al.	Ag-bulk
0.532	8.38×10^{-20}	3.77×10^{-20}	8.80×10^{-20}	3.73×10^{-18}	–	[6] 1993 – Wong et al.	Ag-bulk
1.064	–	–	1.91×10^{-18}	1.34×10^{-17}	–	[7] 1997 – Chang et al.	50-nm film
0.810	–	–	5.45×10^{-20}	1.32×10^{-18}	1.23×10^{-20}	[11] 2004 – Krause et al.	Ag-bulk
1.064	–	5.11×10^{-20}	-2.51×10^{-20}	1.28×10^{-22}	–	[12] 2005 – O'Donnell et al.	Ag-bulk

III. ESTIMATE OF THERMAL BROADENING EFFECTS

In this section, we provide a supplementary estimate of the extent to which pump-induced heating may modify the optical response of the silver film, and therefore contribute to the wavelength dependence of the measured nonlinear signal. This discussion is intended as an order-of-magnitude assessment rather than a complete microscopic treatment.

A fully quantitative description of heat generation and redistribution under ultrafast illumination in nanometric metallic films requires a framework beyond standard bulk models. As discussed in Ref. [15], when the film thickness is on the order of a few nanometers, bulk approaches such as the diffusion equation in the two-temperature model cease to be strictly applicable, and quantum finite-size effects may become relevant. Nevertheless, following the prescription of Ref. [15], one can estimate the influence of heating on the optical response by introducing a temperature-dependent damping rate into a Drude description of silver,

$$\epsilon_{\mathrm{Ag}}(\omega,T,T_l)=\epsilon_b-\frac{\omega_p^2}{\omega\left(\omega+\mathrm{i}\gamma^T\right)}, \tag{S22}$$

which depend on the lattice temperature T_l an the electron temperature T. Here, $\epsilon_b = 4.0$ is an effective background permittivity accounting for screening from interband transitions in the excitation range $\lambda_{\mathrm{in}} =$ 1.4–3.1 μm, and the bulk plasma frequency is taken as $\hbar\omega_p = 9.17$ eV.

The phenomenological damping rate is written as

$$\gamma^T=\gamma^{e\text{-}e}(T)+\gamma^{e\text{-}ph}(T_l), \tag{S23}$$

where the electron–electron and electron–phonon contributions are approximated as

$$\gamma^{e\text{-}e}(T)=\frac{\pi^3\alpha\beta}{12\hbar E_F}k_B^2T^2,\qquad \gamma^{e\text{-}ph}(T_l)\approx\gamma_0\frac{T_l}{\theta_D}. \tag{S24}$$

For silver, we use $\alpha = 0.55$ and $\beta = 0.73$ [16], a Fermi energy $E_F = 5.53$ eV [17], and a Debye temperature $\theta_D = 215$ K[18]. The inelastic broadening parameter is taken as $\hbar\gamma_0 = 15$ meV, which gives a room-temperature damping $\hbar\gamma(T_0,T_0)\approx 21$ meV (with $T_0 = 300$ K). This value is consistent with reported optical linewidths of silver in the near-infrared regime. We also note that, at the wavelengths of interest $\mathrm{Im}\{\epsilon_{\mathrm{Ag}}\}\sim 10$ according to measured optical data [2].

To estimate the magnitude of pump-induced heating, we use the peak intensity corresponding to the average incident power under Gaussian temporal and spatial profiles,

$$I_{\mathrm{peak}}=\frac{f_{\mathrm{shape}}}{f\,\tau\,A}P_{\mathrm{ave}}, \tag{S25}$$

where P_{ave} is the average pump power, f the repetition rate, τ the pulse duration, and $f_{\mathrm{shape}} = 0.94$ accounts for the Gaussian pulse shape. The effective focal area is $A = \pi\sigma_0^2\sec\theta_0$, with σ_0 the beam waist (see Supplementary Figure S9) and θ_0 the incidence angle inside the substrate. The corresponding peak fluence is $F_{\mathrm{peak}} = I_{\mathrm{peak}}\tau$.

For representative experimental parameters, $P_{\mathrm{ave}} = 5$ mW, $\tau = 200$ fs, $f = 76$ MHz, $\sigma_0 = 17$ μm, and $\theta_0 = 45°$, we obtain $I_{\mathrm{peak}}\approx 24$ MW/cm^2, and $F_{\mathrm{peak}}\approx 4.8$ μJ/cm^2. A back-of-the-envelope estimate for the corresponding increase in electron temperature is

$$\Delta T\sim\frac{F_{\mathrm{peak}}(\omega/c)\,\mathrm{Im}\{\epsilon_{\mathrm{Ag}}\}}{C_e}, \tag{S26}$$

where $C_e\sim 10^5\ \mathrm{J\,m^{-3}\,K^{-1}}$ is the electronic heat capacity of silver [19]. This yields $\Delta T\sim 10^1$–10^2 K, depending on the value of $\mathrm{Im}\{\epsilon_{\mathrm{Ag}}\}$.

This estimate suggests that thermal broadening can modify the damping rate only moderately under our excitation conditions. In particular, the resulting correction to $\gamma^T = \gamma^{e\text{-}e} + \gamma^{e\text{-}ph}$ in Eq. (S24) is not expected to constitute the dominant contribution to the observed spectral variation of $\chi^{(2)}$. However, elevated electronic temperatures may still affect state occupancies near the Fermi level and thereby induce more subtle changes in the nonlinear response. Moreover, the thermalization and relaxation dynamics occur on picosecond time scales, which remain much shorter than the ~ 13 ns interval between consecutive pulses at 76 MHz.

We therefore view the present analysis as supporting a limited role for thermal broadening: it provides a plausible correction to the optical response, but by itself it is unlikely to fully account for the mismatch in wavelength dependence between measured and simulated $\chi^{(2)}$. A more complete treatment would require a dielectric-function-informed description incorporating nanoscale confinement, anisotropic optical response, and possible interface-specific symmetry breaking.

IV. DETERMINATION OF POLARIZATION STATE

Due to the substrate tilt and its non–polarization-preserving behavior for non-p-polarized light, only p-polarized excitation was used. The polarization transformation induced by the bare silicon substrate was characterized by polarization tomography. Consequently, the excitation polarization was fixed to p-polarization, and a quarter-wave plate placed in front of a linear polarizer was rotated after the sample and before the polarization independent detector. Using the characterized quarter-wave plate and polarizer, the intensity modulation of the detected fundamental excitation beam could be modeled while correcting for Fresnel coefficients.
Taking into account the modulation of the Fresnel reflections at the interfaces, the polarization state can be described using the following expression [20, 21]:

$$I = \frac{I_0}{2}\left[S_0 - S_1\cos^2(2\theta) + S_2\cos(2\theta)\sin(2\theta) + S_3\sin(2\theta)\right]. \tag{S27}$$

Equation S27 directly relates the detected intensity I to the maximum intensity I_0 and the rotation angle of the quarter-wave plate θ to the Stokes parameters $S = (S_0, S_1, S_2, S_3)$ of the excitation beam after transmission through the bare silicon sample. These parameters can subsequently be converted into the corresponding Jones vector representation, as presented in Supplementary Figure S15. The Fresnel reflections were characterized by measuring the transmitted excitation power through the sample as a function of the linear excitation polarization. The refractive index of silicon at a wavelength of 2.3 μm was taken as $\tilde{n}_{\mathrm{Si}} = 3.47 + i6.57 \cdot 10^{-8}$ [22], while for air $\tilde{n}_{\mathrm{Air}} = 1$ [23] was assumed.

---

[1] Hohenester, U. *Nano and quantum optics: an introduction to basic principles and theory* (Springer Nature, 2019).

[2] Johnson, P. B. & Christy, R. W. Optical constants of the noble metals. *Phys. Rev. B* **6**, 4370–4379 (1972).

[3] Pedersen, T. G., Pedersen, K. & Kristensen, T. B. Optical second-harmonic generation from ag quantum wells on Si (111) 7 × 7: Experiment and theory. *Physical Review B* **60**, R13997–R14000 (1999). URL http://dx.doi.org/10.1103/PhysRevB.60.R13997.

[4] Matranga, C. & Guyot-Sionnest, P. Absolute intensity measurements of the optical second-harmonic response of metals from 0.9 to 2.5 ev. *The Journal of Chemical Physics* **115**, 9503–9512 (2001). URL https://doi.org/10.1063/1.1413741.

[5] Galanty, M. *et al.* Second harmonic generation hotspot on a centrosymmetric smooth silver surface. *Light: Science & Applications* **7**, 49 (2018). URL https://doi.org/10.1038/s41377-018-0053-6.

[6] Wong, E. K. L. & Richmond, G. L. Examination of the surface second harmonic response from noble metal surfaces at infrared wavelengths. *The Journal of Chemical Physics* **99**, 5500–5507 (1993). URL http://dx.doi.org/10.1063/1.465967.

[7] Chang, C. & Lue, J. Optical second harmonic generation from thin silver films. *Surface Science* **393**, 231–239 (1997). URL http://dx.doi.org/10.1016/S0039-6028(97)00557-8.

[8] Hirayama, H., Kawata, T. & Takayanagi, K. Oscillation of the optical second-harmonic generation intensity during ag thin film growth on a Si (111) 7 × 7 surface. *Physical Review B* **64** (2001). URL http://dx.doi.org/10.1103/PhysRevB.64.195415.

[9] Pedersen, K. Second-Harmonic Generation Spectroscopy on Thin Metal Films on Si(111). *Physica Scripta* **T109**, 96 (2004). URL http://dx.doi.org/10.1238/Physica.Topical.109a00096.

[10] Pedersen, K., Pedersen, T. G. & Morgen, P. Surface and interface resonances in second harmonic generation from metallic quantum wells on Si(111). *Physical Review B* **73** (2006). URL http://dx.doi.org/10.1103/PhysRevB.73.125440.

[11] Krause, D., Teplin, C. W. & Rogers, C. T. Optical surface second harmonic measurements of isotropic thin-film metals: Gold, silver, copper, aluminum, and tantalum. *Journal of applied physics* **96**, 3626–3634 (2004). URL https://doi.org/10.1063/1.1786341.

[12] O'Donnell, K. & Torre, R. Characterization of the second-harmonic response of a silver–air interface. *New Journal of Physics* **7**, 154 (2005). URL https://doi.org/10.1088/1367-2630/7/1/154.

[13] Russier-Antoine, I., Benichou, E., Bachelier, G., Jonin, C. & Brevet, P. Multipolar contributions of the second harmonic generation from silver and gold nanoparticles. *The Journal of Physical Chemistry C* **111**, 9044–9048 (2007). URL https://doi.org/10.1021/jp0675025.

[14] Singh, A. K. *et al.* Nonlinear optical properties of triangular silver nanomaterials. *Chemical Physics Letters* **481**, 94–98 (2009). URL https://doi.org/10.1016/j.cplett.2009.09.045.

[15] Rodríguez Echarri, A. *et al.* Nonlinear photoluminescence in gold thin films. *ACS Photonics* **10**, 2918–2929 (2023). URL http://dx.doi.org/10.1021/acsphotonics.3c00644.

[16] Lawrence, W. E. Electron-electron scattering in the low-temperature resistivity of the noble metals. *Phys. Rev. B* **13**, 5316–5319 (1976).

[17] Ashcroft, N. W. & Mermin, N. D. *Solid State Physics* (Harcourt College Publishers, Philadelphia, 1976).

[18] O'Reilly, E. P. *Quantum theory of solids* (Taylor & Francis, New York, 2017).

[19] Lin, Z., Zhigilei, L. V. & Celli, V. Electron-phonon coupling and electron heat capacity of metals under conditions of strong electron-phonon nonequilibrium. *Physical Review B—Condensed Matter and Materials Physics* **77**, 075133 (2008).

[20] Schaefer, B., Collett, E., Smyth, R., Barrett, D. & Fraher, B. Measuring the stokes polarization parameters. *American Journal of Physics* **75**, 163–168 (2007). URL http://dx.doi.org/10.1119/1.2386162.

[21] Chaturvedi, M., Bhandare, R., Kumar, S., Verma, Y. & Raja, S. A compact full stokes polarimeter. *Optik* **267**, 169645 (2022). URL http://dx.doi.org/10.1016/j.ijleo.2022.169645.

[22] Shkondin, E. *et al.* Large-scale high aspect ratio al-doped zno nanopillars arrays as anisotropic metamaterials. *Opt. Mater. Express* **7**, 1606–1627 (2017). URL https://opg.optica.org/ome/abstract.cfm?URI=ome-7-5-1606.

[23] Mathar, R. J. Refractive index of humid air in the infrared: model fits. *Journal of Optics A: Pure and Applied Optics* **9**, 470–476 (2007). URL http://dx.doi.org/10.1088/1464-4258/9/5/008.
