# Supplementary material for: Few-atom-thick silver films for enhanced nanoscale nonlinear optics
Source: Nat Commun. 2026 Jul 1;17:8214. doi: 10.1038/s41467-026-74804-4 (PMC13463075; doi:10.1038/s41467-026-74804-4)
Supplement: Supplementary file 2 [file 41467_2026_74804_MOESM2_ESM.pdf]

Peer Review File

Atomically thin silver films for enhanced nanoscale nonlinear optics

Corresponding Author: Dr Lee Rozema

This file contains all reviewer reports in order by version, followed by all author rebuttals in order by version.

Version 0:

Reviewer comments:

Reviewer #1

(Remarks to the Author)
Jenke and co-authors investigated second harmonic generation (SHG) from atomically thin epitaxial silver films on silicon. The SHG conversion efficiency, taken as the ratio between the detected power at the SHG and the incident power at the fundamental, is stated to increase by almost two orders of magnitude by decreasing the Ag film thickness from 30 to 10 ML. This increase is attributed to an increasingly anharmonic electron motion associated with thickness dependent quantum-well states. DFT-based model calculations that assume SHG to result from out-of plane dipoles confirm the observed trend. The reported findings could be interesting for the field of nanoscale nonlinear optics with potential applications in photonics, sensing, and quantum technologies.

The increase of SHG from thin silver films on silicon for decreasing thickness in the ML range was reported previously by K. Pedersen et al. ("Surface and interface resonances in second harmonic generation from metallic quantum wells on Si(111)", Phys. Rev. B 73, 125440 (2006)). This increase was also attributed to the formation of quantum-well states and supported by the observed oscillations of the SHG power for varying film thickness. In this paper, a phenomenological model for the frequency and thickness dependence was developed that assumed contributions from both interfaces which reproduced the experimental observations. The main novelty of the present manuscript appears to be the utilization of epitaxial films, in contrast to evaporated and annealed Ag films studied in the paper by Pedersen, and the observation of a stronger influence of film thickness on SHG power. The latter could also be related to the different photon energies used in the experiments. The authors need to discuss their results in the context of this earlier work and clearly illustrate the novelty of their work and findings. In its present form, we cannot recommend publication of the manuscript, unfortunately.

Other points:
1. While the calculated SHG conversion efficiencies reproduce the general observation of increased efficiency for thinner films, they differ considerably from the experimental data both in terms of magnitude (eta(theory) = 0.1 * 10^-27 m^2/W vs. eta(exp) = 6000 * 10^-27 m^2/W at 3.1 µm) and predicted trend for different photon energies (highest eta(theory) at 1.8 µm. vs. highest eta(exp) at 2.3 µm). This appears to reflect a systematic deviation rather than a smaller variation based on the mentioned possible thermal and spectral broadening effects.
2. In Fig.4b-d, apparently the oscillations predicted by the model calculations are coming from the QWS, but this is not explicitly stated in the text. Additionally, the trend of the theoretical analysis does not agree with some experimental data points. The experimental data points are not sufficient to confirm the occurrence of the predicted oscillations. We suggest increasing the number of measured data points for different thickness and adding error bars to the experimental results.
3. Please include error bars for the experimental results in Figure 4b-d.
4. From the data shown in Figure 4b-d and in the SI, an increase in SHG power by ~35 can be deduced (for some y scales the ticks are missing). It is not clear how an increase of almost two orders of magnitude was obtained.
5. In Fig. 4a, it is not clear why the measurements were taken in the saturation regime of the detector, since it is possible to use density filters to reduce the detected power and compensate for this in post-processing analysis, which would benefit the interpretation of the results and the clarity of the presentation.
6. The manuscript would benefit from a broader discussion of the origin of the SHG signal, which in this case will arise primarily from surface effects (T. F. Heinz et al., Phys. Rev. Lett. 63, 644 (1989)). In principle, SHG can be expected to arise from both the silicon-Ag and the Ag-air interface. Therefore, it would not be entirely counterintuitive to expect that reducing the film thickness could lead to an increase of the transmitted incident light and of the generated SHG light for the respective interfaces. Notably, the skin depth of light at 1-3 µm is about 25 nm, around only 3 times the studied film thickness and could

thus be expected to impact the nonlinear signal generation and detection.

Reviewer #2

(Remarks to the Author)
Dear Editor,
The manuscript by Jenke et al., “Atomically thin silver films for enhanced nanoscale nonlinear optics,” reports SHG studies on epitaxial Ag(111) films a few monolayers thick, combining structural (ARPES, XPS, STM) and optical characterization with a quantum-mechanical model. The authors observe up to two orders of magnitude enhancement in nonlinear efficiency as the film approaches the quantum-confinement regime, attributed to anharmonic electron dynamics within quantized quantum-well states.

While the topic is timely, similar QWS-mediated effects have been reported previously, and the present work appears to be a quantitative refinement rather than a conceptual breakthrough. Key issues remain regarding normalization, interface contributions, and theoretical interpretation. I am not yet convinced the study meets the novelty and general-interest threshold for Nature Communications, but a thoroughly revised version addressing these questions could warrant reconsideration.

>>Regarding Novelty and Positioning: The idea that QWS in Ag(111) affect SHG is not entirely new.
The idea that quantum-well states (QWS) in ultrathin Ag(111) films can influence second-harmonic generation (SHG) is well established, as demonstrated by Pedersen et al. (PRB 60, R13997 (1999)) and Hirayama et al. (PRB 64, 195415 (2001)), who reported oscillatory SHG variations with Ag thickness due to quantum confinement and parity alternation of electronic states. The present work differs mainly in its reported magnitude and monotonic trend, an enhancement of up to two orders of magnitude as thickness decreases from ~30 ML to 10 ML. However, to convincingly establish novelty beyond prior QWS-based studies, the authors must clearly define what this enhancement is measured against and quantify the improvement relative to earlier results.
Is the two-order-of-magnitude increase calculated relative to bulk single-crystal Ag, to the thickest (30 ML) sample in the current series, or to previously reported Ag/Si(111) data from the literature?
Are the conversion efficiencies normalized to interaction volume, illuminated area, or total film thickness?
How does the extracted $\chi^{(2)}$ (in pm V^{-1} or $m^2 V^{-1}$ units) compare to previously reported surface and interface nonlinear coefficients for Ag, Au, or Cu films of comparable thickness?
Furthermore, earlier studies reported oscillatory SHG behavior with thickness due to discrete subband transitions, whereas the present work claims a monotonic enhancement. Yet, Fig. 4 shows only five experimental points and theoretical curves that still exhibit oscillatory features, making the monotonic trend inconclusive. If this difference arises from improved crystallinity, reduced scattering, or better-defined QWS spacing, the authors should emphasize these aspects as the genuine source of novelty.
It would also strengthen the case if the authors demonstrate that their approach allows:
Quantitative extraction of absolute $\chi^{(2)}$ values under well-calibrated excitation conditions;
Enhanced film stability and reproducibility due to their Si-capping strategy and epitaxial control; and
Direct correlation between ARPES-resolved QWS and nonlinear response, something rarely achieved in prior work.
SHG conversion efficiencies is plotted without error rate which is better to include so that the potential reader can also observe accuracy (and/or precision) of the reported data.
How the structure planar orientation upon the measured quantities would get affect by this: polarization resolved/anisotropic SHG? It would be better show this as a systematic study. (I will lay this out further below.)
[1] Hirayama H, Komizo T, Kawata T, Takayanagi K. Optical second harmonic generation spectrum of Ag/Si (111) reconstructed surfaces. Physical Review B. 2001 Mar 29;63(15):155413.
[2]Chang, C. S., and J. T. Lue. "Optical second harmonic generation from thin silver films." Surface science 393.1-3 (1997): 231-239.
>>Quantitative Comparison with Literature
One of the central weakness of the current manuscript is the lack of quantitative benchmarking against prior experimental and theoretical studies of nonlinear optical response in ultrathin metallic films. While the authors report a “two-order-of-magnitude enhancement” in SHG efficiency, no clear numerical comparison is provided with previous Ag(111) QWS sytems or other atomically thin plasmonic films. Without such contextualization, the reader cannot assess whether the observed enhancement is genuinely unprecedented or primarily a relative improvement within this specific dataset.
Previous works—such as Chang & Lue, Surface Science 393, 231 (1997); Wong & Richmond, J. Chem. Phys. 99, 5500 (1993); and Pedersen et al., Phys. Rev. B 60, R13997 (1999), reported $\chi^{(2)}$ coefficients for Ag thin films in the range of 10^{-21} to 10^{-20} $m^2 V^{-1}$, depending on surface orientation and growth conditions. Even for nanostructured or resonantly enhanced Ag configurations, values rarely exceeded ~10^{-19} $m^2 V^{-1}$. The present manuscript, by contrast, implies $\chi^{(2)}$ values approaching or exceeding this upper bound, yet these numbers are not explicitly stated nor normalized for experimental parameters such as beam area, incidence angle, or local field corrections.
To enable meaningful evaluation and reproducibility, let me kindly suggest authorsl
Tabulating experimental $\chi^{(2)}$ values (in $m^2 V^{-1}$ or pm V^{-1}) extracted for each thickness and excitation wavelength, accompanied by estimated uncertainties and calibration methodology.
Including a comparative summary table listing literature-reported $\chi^{(2)}$ values for Ag(111), (relevant nanostructured films and even howabout Au(111), Cu(111)?), clearly identifying whether the values correspond to bulk, surface, or interface contributions.

Differentiating between absolute and relative enhancements: For instance, whether the “two orders of magnitude” refers to integrated SHG power, peak intensity, or normalized susceptibility.
Clarifying the normalization procedure used in Figs. 4b–d: are the data corrected for optical throughput, detector response, and Fresnel transmission across the Si substrate and capping layer?
Additionally, the theoretical predictions in Fig. 4e yield $\chi^{(2)}$ values of comprable magnitude but display different wavelength dependence. Quantitative comparison of these results with experimental data, possibly through a combined plot of experiment vs. theory vs. literature, would significantly strengthen themanuscript’s credibility and reveal how the proposed model extends or refines earlier phenomenological treatments.
Finally, because the work positions itself as a quantum-engineered platform rather than a purely plasmonic one, it is critical to emphasize the degree of enhancement relative to previously reported QWS oscillatory behavior. The authors might show that while earlier studies achieved modest modulation amplitudes (≤10×), the present atomically controlled films exhibit a monotonic, 100×–200× increase with superior long-term stability, thus establishing a genuine quantitative leap.
Figure 2 effectively demonstrates structural and electronic order, validating sample quality, but its analysis could be expanded with quantitative ARPES interpretation (e.g., QWS energy spacing vs. thickness) and correlation with nonlinear measurements for a stronger mechanistic connection.
In summary, the manuscript will benefit from a clearer articulation of how its findings extend beyond the foundational studies of QWS-mediated SHG on whether through improved experimental precision, expanded wavelength coverage, quantitative susceptibility extraction, or long-term stability of atomically thin metallic films. A concise comparison table summarizing literature $\chi^{(2)}$ values and enhancement factors would make this distinction unambiguous. The claim of “two orders of magnitude enhancement” needs explicit normalization (e.g., relative to bulk Ag or to previous QWS films). Please check the following works including but not limited to (as there are several others..)
[1] Pedersen, T.G., Pedersen, K. and Kristensen, T.B., 1999. Optical second-harmonic generation from Ag quantum wells on Si (111) 7× 7: experiment and theory. Physical Review B, 60(20), p.R13997.
[2] Chang, C.S. and Lue, J.T., 1997. Optical second harmonic generation from thin silver films. Surface science, 393(1-3), pp.231-239.
[3] Wong, E.K.L. and Richmond, G.L., 1993. Examination of the surface second harmonic response from noble metal surfaces at infrared wavelengths. The Journal of chemical physics, 99(7), pp.5500-5507.

>>Broader Impact and Missing Perspective
While the manuscript establishes a clear observation of enhanced nonlinear response in atomically thin silver films, it does not sufficiently articulate the broader scientific and technological context where such an increase in $\chi^{(2)}$ can drive new physics or device concepts. From aspect of potential readers, the discussion should go beyond immediate SHG efficiency gains and convey why these materials are relevant to next-generation photonic systems.
Specifically, the authors could enrich the Discussion by addressing how the demonstrated large nonlinearities may interface with ongoing advances in:
Tunable and reconfigurable nonlinear systems – e.g., electrical, optical, or strain-based modulation of QWS to dynamically control harmonic generation.
Chiral and nonreciprocal photonics: since the broken inversion symmetry at the Ag/Si interface and the anisotropic QWS dispersion could, in principle, enable circular dichroism or nonlinear optical isolation effects when combined with magnetic or spin–orbit perturbations.
Hyperbolic and plasmonic metamaterials: integration of these atomically thin metals into multilayer stacks or metasurfaces could yield broadband enhancement and phase-engineered nonlinear responses.
Quantum and single-photon nonlinearities: the strong field confinement and quantum-level anharmonicity might offer a route toward nonlinear photon–electron interactions in mesoscopic cavities or quantum emitters…
[1] Bhowmik, G., An, Y.Q., Schujman, S., Diebold, A.C. and Huang, M., 2020. Optical second harmonic generation from silicon (100) crystals with process tailored surface and embedded silver nanostructures for silicon nonlinear nanophotonics. Journal of Applied Physics, 128(16).
[2] Ohnoutek, L., Olohan, B.J., Jones, R.R., Zheng, X., Jeong, H.H. and Valev, V.K., 2022. Second harmonic Rayleigh scattering optical activity of single Ag nanohelices in a liquid. Nanoscale, 14(10), pp.3888-3898.
[3] Olohan, B.J., Petronijevic, E., Kilic, U., Wimer, S., Hilfiker, M., Schubert, M., Argyropoulos, C., Schubert, E., Clowes, S.R., Pantoş, G.D. and Andrews, D.L., 2024. Chiroptical second-harmonic tyndall scattering from silicon nanohelices. ACS nano, 18(26), pp.16766-16775.
[4] Kilic, U., Hilfiker, M., Ruder, A., Feder, R., Schubert, E., Schubert, M. and Argyropoulos, C., 2021. Broadband enhanced chirality with tunable response in hybrid plasmonic helical metamaterials. Advanced Functional Materials, 31(20), p.2010329.
[5] Kolmychek, I.A., Malysheva, I.V., Novikov, V.B., Leontiev, A.P., Napolskii, K.S. and Murzina, T.V., 2020. Phase-matched optical second harmonic generation in a hyperbolic metamaterial based on silver nanorods. Physical Review B, 102(24), p.241405.
[6] Dickinson, T., Afxenti, I., Astrauskaite, G., Hirsch, L., Nerenberg, S., Jedrkiewicz, O., Faccio, D., Müllenbroich, C., Gatti, A., Clerici, M. and Caspani, L., 2025. Quantum-enhanced second harmonic generation beyond the photon pairs regime. Science Advances, 11(27), p.eadw4820.

>> Incomplete Polarization and Symmetry Analysis
While the manuscript provides a clear observation of second-harmonic generation (SHG) as a function of Ag film thickness, the polarization-resolved analysis is notably limited. All reported measurements are performed in the p→p configuration, with the authors noting that excitation through the Si substrate preserves only p-polarization. However, for a material system whose nonlinear response arises primarily from surface and interface symmetry breaking, such a restriction precludes a full understanding of the tensorial structure of $\chi^{(2)}$ and the associated selection rules governing the observed enhancement. In centrosymmetric metals like silver, the bulk electric-dipole contribution to SHG is forbidden under the electric-dipole

approximation, and the observed signal therefore originates from:
Surface dipole contributions (due to inversion symmetry breaking at the vacuum–metal interface),
Interface contributions (arising at the metal–substrate boundary, particularly at Ag/Si(111)), and
Field-induced multipole or magnetic-dipole terms that become significant in ultrathin or confined geometries.
Without polarization-resolved SHG or azimuthal angle-dependent measurements, it is difficult to distinguish among these possible contributions. The resulting interpretation that the enhancement stems from solely from quantum-well-state (QWS) confinement and anharmonic electron motion, remains plausible but not conclusively demonstrated. To strengthen this section, the authors should consider or at least discuss the following:
Perform or simulate polarization-dependent SHG (e.g., p→p, p→s, s→p) to extract effective tensor components (Xzzz, Xxxz, Xxzx, etc.) and confirm whether the response is dominated by surface, interface, or field-induced contributions.
Include an analysis of symmetry breaking at the Ag/Si(111) interface, particularly since the Si(111) substrate exhibits surface reconstructions that inherently lack inversion symmetry. This could contribute significantly to $\chi^{(2)}$ and might even dominate the signal for the thinnest films.
Given that the Ag/Si(111) interface naturally breaks inversion symmetry and could induce spin–orbit coupling effects, have the authors considered whether the observed enhancement might partially stem from interfacial or topological-like electronic states that contribute to $\chi^{(2)}$? In particular, could hybridized QWS–interface modes enable nontrivial charge or mode coupling across the Ag/Si boundary? If so, a short discussion on this would actually strengthen the manuscript's broader impact, showing awareness of emerging quantum–topological photonics connections.
>> Comparison Between Experiment and Theory plus Dielectric Function Analysis
The inclusion of a quantum-mechanical model is conceptually strong, but the comparison between experiment and theory remains qualitative and idealized. The model assumes symmetric confinement and a free-electron–like response, neglecting the actual dielectric function ε(ω) of the Ag/Si(111) heterostructure. Since SHG efficiency depends critically on local field distributions governed by ε(ω), this simplification limits the quantitative validity of the predicted $\chi^{(2)}$.

In ultrathin epitaxial films, the dielectric function can deviate significantly from bulk Ag due to quantum confinement, substrate coupling, and surface or capping-induced asymmetry.
Incorporating realistic dielectric dispersion, either from spectroscopic ellipsometry measurements or from ab initio optical-response calculations would make the modeling far more physically grounded. Ellipsometry-based retrieval of ε(ω) as a function of thickness could also reveal anisotropic optical behavior, which would independently support or challenge the polarization dependence inferred from SHG measurements. Such dielectric-function measurements would also establish a link between optical anisotropy and the tensorial components of $\chi^{(2)}$, complementing polarization-resolved SHG data.

Additionally, the mismatch in wavelength dependence between measured and simulated $\chi^{(2)}$, currently attributed to "thermal broadening," remains speculative. A brief parameter-sensitivity analysis exploring how $\chi^{(2)}$ varies with damping, dielectric anisotropy, or surface asymmetry would clarify whether the model can reproduce experimental trends. Acknowledging these limitations and discussing potential extensions toward dielectric-function-informed modeling would substantially strengthen the theoretical interpretation.

Reviewer #3

(Remarks to the Author)
The manuscript "Atomically thin silver films for enhanced nanoscale nonlinear optics" by Philipp K. Jenke, et al. (2025) investigates how crystalline ultra-thin silver films, passivated with silicon, address the challenge of the inherently weak nonlinear optical response in bulk materials. Specifically, the study demonstrates an enhancement of the second-harmonic generation (SHG) as the film thickness decreases, attributed to modifications in the electronic band structure compared to bulk silver. The authors prepared the films in an ultra-high vacuum chamber (1.0 x10-10 mbar) using electron-beam evaporation and characterized them with techniques such as low-energy electron diffraction (LEED), angle-resolved photoemission spectroscopy (ARPES), scanning tunneling microscopy (STM), X-ray photoelectron spectroscopy (XPS), and scanning electron microscopy (SEM). SHG measurements were performed with a modified focus-scan optical transmission setup using femtosecond laser pulses (~200 fs), while density functional theory (DFT) calculations were conducted with VASP simulation package.

The study reveals a strong thickness-dependent nonlinear optical response in atomically thin Ag films, showing that SHG efficiency increases by nearly two orders of magnitude as the thickness decreases. The authors attribute this enhancement to the formation of quantum-well states in the confined electronic structure. The paper provides a clear description of the optical setup, particularly regarding the sample orientation to minimize SHG contributions from the Si (111) substrate. However, the rationale for selecting a 45° incidence angle could have been discussed in greater detail. The comparison between experimental and theoretical SHG efficiency is well structured, highlighting both the agreements and discrepancies between the results. Nevertheless, it would have been useful to reference and contrast these findings with those from "Second-Harmonic Generation Spectroscopy on Thin Films on Si (111) by Kjeld Pedersen" [1], especially the section concerning Ag/Si(111) – 7X7. Finally, a brief discussion of how the silica capping layer influences (or does not influence) the SHG response would strengthen the analysis, considering that even though bulk SiO2 is non-centrosymmetric, surface symmetry breaking can induce SHG[2][3].

Overall, this paper provides valuable insight into how quantum confinement in ultra-thin metal films alters their electronic structure and enhances nonlinear optical responses, contributing to the development of advanced nanophotonic and nonlinear optical materials.

References:

[1] “Second-Harmonic Generation Spectroscopy on Thin Metal Films on Si(111),” Phys. Scr., vol. T109, p. 96, 2004, doi: 10.1238/Physica.Topical.109a00096.
[2] M. Cazzanelli and J. Schilling, “Second order optical nonlinearity in silicon by symmetry breaking,” Appl. Phys. Rev., vol. 3, no. 1, p. 011104, Mar. 2016, doi: 10.1063/1.4941558.
[3] F. Abtahi et al., “Enhanced surface second harmonic generation in nanolaminates,” Opt. Express, vol. 31, no. 7, p. 11354, Mar. 2023, doi: 10.1364/OE.484893.

Reviewer #4

(Remarks to the Author)
I co-reviewed this manuscript with one of the reviewers who provided the listed reports. This is part of the Nature Communications initiative to facilitate training in peer review and to provide appropriate recognition for Early Career Researchers who co-review manuscripts.

Reviewer #5

(Remarks to the Author)
I co-reviewed this manuscript with one of the reviewers who provided the listed reports. This is part of the Nature Communications initiative to facilitate training in peer review and to provide appropriate recognition for Early Career Researchers who co-review manuscripts.

Version 1:

Reviewer comments:

Reviewer #1

(Remarks to the Author)
In the revised manuscript, the authors have partially addressed the previously raised concerns. The main text now includes references to earlier work by Pedersen and others regarding the observed correlation between the SHG intensity of crystalline silver films and their thickness. Based on the authors’ response, the primary novelty of the present study lies in the fabrication and investigation of epitaxial films, as well as in the development of an extended theoretical model presented in the supplementary information of the revised version. At this stage, the manuscript may be considered for publication, provided that the following points are addressed.

1 The main manuscript does not clearly convey that the correlation between SHG intensity in crystalline silver films and their thickness has already been experimentally studied over a much broader range of layer numbers than what is presented here. Reference 41 is cited only in the context of a phenomenological model and appears just in the supplementary information. A more explicit discussion of prior literature and its findings in the main text would be necessary to enable readers to properly evaluate the novelty and significance of the present work.

2. Although the title refers to the investigation of atomically thin silver films, the experimental and theoretical analyses presented in the main text are limited to thicknesses above 10 monolayers (10–30 ML). Referring to these samples as few-atom-thick silver films, as done in the manuscript, is therefore more appropriate. In light of prior work summarized in Table S3 of the supplementary information, which explores significantly thinner regimes, it would be advisable to revise the manuscript title to accurately reflect the studied thickness range, for example, “Few-atom-thick silver films …”.

3. We understand that the fabrication of samples with other layer numbers to differentiate an oscillatory behavior of chi^2 as observed earlier would be technically demanding. Perhaps this question could be addressed by including additional theoretical data points in Figure 4d.

4. Line 308 should probably read Fig. 4d instead of Fig. 4e.

Reviewer #2

(Remarks to the Author)
Dear Editor,
I would like to thank the authors for their detailed and thoughtful responses, as well as for the substantial revisions made to the manuscript. The clarity and overall quality of the work have improved significantly. In particular, the revisions addressing normalization of SHG efficiencies, inclusion of error bars, and expanded discussion of the experimental results and theoretical framework have strengthened the manuscript.
The combined experimental and theoretical effort presents an interesting platform for investigating nonlinear optical responses in ultra-thin metallic systems. The results are now more clearly presented, and the main conclusions are better supported than in the initial submission.

As a follow up, I have a few minor/major comments that, if addressed, would further improve the clarity and rigor of the manuscript:

1a) QWS–SHG connection: The ARPES data, including the energy distribution curves provided in the Supplementary Information, convincingly establish the presence and thickness dependence of quantum-well states (QWS) in the Ag films. However, the connection between these states and the observed SHG enhancement remains largely qualitative. Clarifying that the ARPES results establish the electronic-structure basis for the proposed mechanism, while the link to SHG enhancement is inferred rather than quantitatively demonstrated, would improve the interpretation.
1b) QWS-to-continuum crossover: Moreover, Fig.2 clearly demonstrates the presence of quantum-well states and their thickness dependence. However, the transition from discrete levels in thin films to a quasi-continuous electronic structure in thicker films, as illustrated conceptually in Fig. 1, is not directly evident in the experimental data, where the subbands remain well resolved even at higher thicknesses. Am I missing something that has already existed in the text if not I kindly recommend, a brief clarification distinguishing the conceptual illustration from the experimentally accessible regime would improve clarity.
2) Polarization and symmetry considerations: The authors explain that excitation through the silicon substrate preserves predominantly p polarization, which justifies the focus on p polarized excitation and the absence of a full polarization dependent SHG study.
a)However, it would be helpful to more explicitly state the implications of this limitation for the interpretation of the results. In particular, the lack of polarization resolved SHG measurements prevents the separation of different contributions to the nonlinear response, for example interface versus field induced effects, and precludes access to individual tensor components of the second order susceptibility. A brief clarification alongthese lines would help guide the reader on the scope of the conclusions.
b)In addition, while Fig. S15 provides useful information on the polarization state after transmission through the silicon substrate, presented on a Poincare sphere based on reported Jones vectors, it is not immediately clear whether these results are obtained experimentally or derived from a model, and the methodology used to determine the Jones vectors is not described. A short clarification of how the polarization state was determined would improve transparency.
3) Uncertainty at larger thicknesses: In Fig. 4 (particularly panel d), the experimental uncertainty appears to increase noticeably for larger film thicknesses (e.g., $t \approx 30$ ML). While this is likely related to reduced SHG signal strength and associated signal-to-noise limitations, this behavior is not explicitly discussed. A brief clarification regarding the origin of the increased uncertainty and its implications for interpreting the thickness dependence would improve the presentation.
In addition, in Fig. 4d and Figs. S12 and S13, the theoretical results show instances where the responses for different excitation wavelengths appear to overlap at certain thicknesses, while at neighboring thickness values they diverge more noticeably. It would be helpful to briefly clarify the physical origin of this behavior, in particular whether it arises from thickness dependent shifts in quantum well state energies, variations in resonance conditions, or model assumptions such as fixed damping.
4) Dielectric function and Model scope limitations: and quantitative deviations: The manuscript appropriately emphasizes that the theoretical model is intended to capture the dominant physical trends rather than provide a fully quantitative description. Given the observed discrepancies between experiment and theory, it would be helpful to briefly clarify which physical parameters (e.g., damping, effective masses, or interface contributions) are expected to have the largest impact on the quantitative agreement. Such a discussion would help guide the reader on how the present phenomenlogical framework could be extended in future work without requiring additional fitting in the current manuscript.
5a) Interpretation of theoretical spectral features and Extension beyond experimentally accessible thickness range: The theoretical results in Figs. S11 and S12 explore thickness regimes that extend to or approach values below those experimentally realized in the present study. It would be helpful if the authors could briefly comment on how the predicted nonlinear response is expected to evolve in such ultra thin regimes, and how these idealized predictions should be interpreted in light of experimental constraints such as film continuity, increased disorder, and structural inhomogeneity. In particular, a short discussion clarifying whether the strong spectral features predicted by the model at very low thicknesses would remain observable or become broadened or suppressed under realistic conditions would help guide the reader in assessing the physical relevance of the theoretical extrapolation.
5b) Clarification of Fig. 1: It would be helpful to explicitly clarify whether Fig. 1 represents a conceptual or theoretical illustration rather than experimental data. While it effectively conveys the expected trnsition from discrete quantum well states to a quasi-continuous electronic structure with increasing thickness, this crossover is not directly evident in the experimental ARPES results shown in Fig. 2. A brief clarification in the caption or main text would improve readability and avoid potential confusion.
6) Consistency of mechanistic interpretation: In the paragraph discussing the thickness dependence of the SHG response (Results section, around Fig. 4), the manuscript states: “This indicates that the rise in second-order susceptibility is due to thickness-dependent changes in the electronic band structure of the material.”
As the authors acknowledge that both intrinsic (quantum-well–induced band-structure modifications) and extrinsic factors (e.g., reduced reabsorption, local field enhancement, and interface contributions) contribute to the observed SHG enhancement, noting that these effects act cooperatively. For consistency, it would be beneficial to clarify this and to reflect the combined role of these mechanisms already described in the manuscript.

Reviewer #3

(Remarks to the Author)
The revised manuscript has satisfied my original concerns. I support publication.

Reviewer #4

(Remarks to the Author)
I co-reviewed this manuscript with one of the reviewers who provided the listed reports. This is part of the Nature Communications initiative to facilitate training in peer review and to provide appropriate recognition for Early Career Researchers who co-review manuscripts.

Version 2:

Reviewer comments:

Reviewer #1

(Remarks to the Author)
In this revised version of the manuscript and their extensive response letter, the authors addressed all major points. At this point, I suggest publication of the manuscript in its present form.

Reviewer #2

(Remarks to the Author)
I thank the authors for their careful responses and for the substantial revisions and clarifications made throughout the manuscript, main text, and Supporting Information. I believe that the authors have taken the comments and concerns seriously and have significantly improved the overall clarity, rigor, and transparency of the work accordingly.

While my perspective on some aspects of the interpretation and theoretical/experimental scope may still partially differ from that of the authors, I appreciate their efforts in clarifying the intended scope, assumptions, and limitations of the study.

At this stage, I do not have further comments or suggestions. Overall, I support publication of the manuscript in its present revised form.

Reviewer #4

(Remarks to the Author)
I co-reviewed this manuscript with one of the reviewers who provided the listed reports. This is part of the Nature Communications initiative to facilitate training in peer review and to provide appropriate recognition for Early Career Researchers who co-review manuscripts.

Open Access This Peer Review File is licensed under a Creative Commons Attribution 4.0 International License, which permits use, sharing, adaptation, distribution and reproduction in any medium or format, as long as you give appropriate credit to the original author(s) and the source, provide a link to the Creative Commons license, and indicate if changes were made.
In cases where reviewers are anonymous, credit should be given to 'Anonymous Referee' and the source.
The images or other third party material in this Peer Review File are included in the article’s Creative Commons license, unless indicated otherwise in a credit line to the material. If material is not included in the article’s Creative Commons

license and your intended use is not permitted by statutory regulation or exceeds the permitted use, you will need to obtain permission directly from the copyright holder.
To view a copy of this license, visit https://creativecommons.org/licenses/by/4.0/

Response to the Referees' reports

In the following, the Referee's comments are shown in grey boxes. For better transparency, we have added to the bibliography the References cited by the Referees. Our responses to the Referee's comments are presented in plain text. Changes made to the manuscript are highlighted in light blue boxes. Line numbers in this boxes refer to those of the initially submitted manuscript. Within the light blue boxes, newly added text to the manuscript is marked in green, and the removed text is shown in red with strikethrough.

1 Reviewer 1

> Jenke and co-authors investigated second harmonic generation (SHG) from atomically thin epitaxial silver films on silicon. The SHG conversion efficiency, taken as the ratio between the detected power at the SHG and the incident power at the fundamental, is stated to increase by almost two orders of magnitude by decreasing the Ag film thickness from 30 to 10 ML. This increase is attributed to an increasingly anharmonic electron motion associated with thickness dependent quantum-well states. DFT-based model calculations that assume SHG to result from out-of plane dipoles confirm the observed trend. The reported findings could be interesting for the field of nanoscale nonlinear optics with potential applications in photonics, sensing, and quantum technologies.

We thank the Reviewer for the effort invested in reviewing our manuscript and for the constructive feedback provided. We share the view that our findings are interesting and relevant for the field of nanoscale nonlinear optics, with potential application in photonics, sensing, and quantum technologies.

1.1

> The increase of SHG from thin silver films on silicon for decreasing thickness in the ML range was reported previously by K. Pedersen et al. ("Surface and interface resonances in second harmonic generation from metallic quantum wells on Si(111)", Phys. Rev. B 73, 125440 (2006) [1]). This increase was also attributed to the formation of quantum-well states and supported by the observed oscillations of the SHG power for varying film thickness. In this paper, a phenomenological model for the frequency and thickness dependence was developed that assumed contributions from both interfaces which reproduced the experimental observations. The main novelty of the present manuscript appears to be the utilization of epitaxial films, in contrast to evaporated and annealed Ag films studied in the paper by Pedersen, and the observation of a stronger influence of film thickness on SHG power. The latter could also be related to the different photon energies used in the experiments. The authors need to discuss their results in the context of this earlier work and clearly illustrate the novelty of their work and findings. In its present form, we cannot recommend publication of the manuscript, unfortunately.

We thank Reviewer 1 for the valuable insight and the suggestion to further emphasize the unique aspects of our work compared to Ref. [1]. We believe that our primary contributions are two-fold. First, in contrast to the previous study, our fabrication method enables unrestricted-area epitaxial growth rather than relying on evaporated and subsequently annealed films. The high crystallinity of our samples is confirmed through ARPES measurements and additional LEED data. Furthermore, we provide comprehensive optical characterization of the films in the mid-infrared (MIR) range, which opens a pathway toward future plasmonic applications in this spectral region. Second, while Kirilyuk et al. [2] (in noble-metal films) and Pedersen et al. [1, 3, 4] (in silver films) laid important groundwork by introducing a phenomenological model, our analytical approach fully captures the quantum-well states and their nonlinear optical interactions. Although Pedersen et al. [1, 3, 4] characterized the SHG conversion efficiencies and analyzed the

thickness dependence, to the best our knowledge, our work introduces the first complete theoretical model converting these efficiencies into second-order susceptibilities.

In summary, we are convinced that the combination of high-quality, well-characterized crystalline noble-metal films, detailed SHG measurements, and an analytical theoretical description makes our work a solid foundation for future engineering of nonlinear effects in nanophotonic platforms. We acknowledge the pioneering contributions of Refs. [1–7], and at the same time emphasize that our fabrication method is scalable, our structural and optical characterization is more extensive, our SHG analysis more detailed, and our theoretical model goes beyond the previously proposed phenomenological frameworks.

We have added several sentences to emphasize the novelty of our work in comparison to previous studies by making the following changes:

> **Line 120:**
>
> We provide both extensive material characterizations and quantitative nonlinear-optical characterizations. This combination, together with theory that goes beyond the phenomenological QWS model discussed in previous work, [1,3,4,7] is rarely achieved and enables experimental access to second-order nonlinear susceptibilities in the mesoscopic regime of noble metals.

We added the framework required to apply our theoretical model to the experimental results by including the following in the main text:

> **Line 231:**
>
> The skin-depth of silver at the optical wavelength of $\sim \mu$m is of the order of 10 nm, therefore a few-atom-thick silver films (below 50 ML) act as a two-dimensional metallic screen. Based on the idea of having a two-dimensional structure generating radiation at the second harmonic, we obtain an expression for the resulting second harmonic susceptibility $\chi_b^{(2)}$ in Fig. 4d (see derivation in the SI Sec. S1 and comparison to other works in SI Tab. S4):
>
> $$\left|\chi_b^{(2)}\right| = \frac{|E_0/E_z|^2}{\left|1 + r_p^{\mathrm{Air}\to\mathrm{Si}}\right|} \frac{\sqrt{(2c/\pi)\cos\theta_0\,\eta}}{4\pi\, kt\tan\theta_0}, \qquad (1)$$
>
> in Gaussian units, as a function of the measured $\eta(t,\lambda)$ efficiencies, and the ratio of the impinging p-polarized electric field driving the nonlinear response $E_z/E_0 = t_p^{\mathrm{Air}\to\mathrm{Si}}\, t_p^{\mathrm{Si}\to\mathrm{Air}} \sin\theta_0$ traversing the substrate, with r and t the corresponding reflection and transmission coefficients, respectively.

We added the following text on the film quality:

> **Line 111:**
>
> Here, we demonstrate that QWS in long-term stable (compare SI Fig. S6) wafer-scale ultra-thin crystalline silver can enhance...

1.2

> While the calculated SHG conversion efficiencies reproduce the general observation of increased efficiency for thinner films, they differ considerably from the experimental data both in terms of magnitude

> $\eta_{\text{theory}} = 0.1 \cdot 10^{-27}$ m^2/W vs. $\eta_{\text{exp}} = 6000 \cdot 10^{-27}$ m^2/W at 3.1 μm) and predicted trend for different photon energies (highest η_{theory} at 1.8 μm. vs. highest η_{exp} at 2.3 μm). This appears to reflect a systematic deviation rather than a smaller variation based on the mentioned possible thermal and spectral broadening effects.

We carefully revisited our analysis. Indeed, when converting from atomic units to SI units, we had missed a prefactor in the theoretical data shown in Fig. 4. We have now included this factor, which brings the theoretical and experimental values into closer agreement. We have revised Fig. 4 accordingly, taken the updated prefactor into account, and made the corresponding changes to all affected Supporting Information figures that display theoretical data.

Despite these corrections, a reasonable discrepancy between the theoretical and experimental data remains in Fig. 4d, where the second-order nonlinear susceptibility $\chi^{(2)}$ is compared. This can be explained as follows.

Our theoretical description is based on a model in which the in-plane electronic structure is approximated by parabolic quantum-well dispersions. This approach correctly captures the overall thickness-dependent trend but cannot reproduce the full spectral shape of realistic quantum-well states. Within this model, the position of the calculated SHG maxima is determined by the resonance conditions of the quantized states. These resonances are sensitive to parameters such as the effective masses used; even small variations shift the subband spacings and therefore the wavelength at which the SHG peak appears. In addition, the model does not include the presence of the substrate in the calculation of the QWs, which is known to modify the quantum-well energies, shift the resonance positions, and introduce symmetry-breaking effects that further enhance SHG. Regarding the absolute magnitude, the effective masses also influence the transition strengths and, therefore, the overall SHG yield. A second important factor is the linewidth: ARPES studies show that individual QWS exhibit different inelastic scattering rates (see Fig. S2), whereas our model employs a constant damping parameter. Reducing this damping leads to a substantial increase in the calculated SHG; however, here we adopted a conservative broadening of 0.1 eV. Given these sensitivities, and because several experimentally relevant mechanisms are not included in the simplified model, quantitative discrepancies in both amplitude and peak position are expected. Importantly, the calculated results do reproduce the key experimental trend: thinner films consistently exhibit stronger SHG. The purpose of the model is capturing well the generic thickness dependence arising from quantum confinement and resonant enhancement, while the listed effects can explain its deviation from the experimental data.

We made the following changes to the manuscript:

> **Line 251:**
>
> However, the variation in calculated ~~SHG conversion efficiencies~~ second-order nonlinear susceptibility with incident light wavelength differs from the experimental results. This discrepancy originates from the assumptions underlying the theoretical model. In particular, the model employs parabolic quantum-well subbands, neglects the presence of the substrate, and assumes a single, energy-independent linewidth. While the effective masses used in the model are extracted from density-functional-theory (DFT) calculations by fitting the low-energy dispersion of the quantum-well states to parabolas, the full DFT band structures reveal deviations from a harmonic behavior. Moreover, the interaction with the Si substrate modifies the band dispersions and effective masses, thereby altering the carrier dynamics. ARPES data in Fig. 2 further demonstrate that the electronic linewidths are subband- and energy-dependent rather than uniform. Although surface-enabled Landau damping is implicitly captured in the model through an effective phenomenological linewidth, its detailed energy-, subband-, and thickness-dependent character is not explicitly resolved. Since the SHG response is highly sensitive to subband energies, effective masses, and electronic lifetimes, quantitative deviations in both the magnitude and spectral position of the SHG features are therefore expected. In addition, thickness-dependent ultra-fast thermal and electronic broadening effects in the silver film [8] and the environment, which are not included in the present model, may further contribute to the observed differences between theory and experiment. We emphasize that the purpose of the present model is to capture the dominant physical trends governing the thickness dependence of the SHG response

rather than to provide a fully quantitative description. A more complete treatment would necessitate substantially more complex theoretical and computational methods and lies beyond the scope of the present work.

1.3

In Fig.4b-d, apparently the oscillations predicted by the model calculations are coming from the QWS, but this is not explicitly stated in the text. Additionally, the trend of the theoretical analysis does not agree with some experimental data points. The experimental data points are not sufficient to confirm the occurrence of the predicted oscillations. We suggest increasing the number of measured data points for different thickness and adding error bars to the experimental results.

We thank the Reviewer for raising these points. In response, we have revised Figure 4 as follows. Panels b–d have been consolidated into panel c, and a new panel has been added as panel b. Experimental and theoretical data for the second-order susceptibility are now shown together in panel d, which previously corresponded to panel e. We have also added error bars to the experimental data in Fig. 4.

The advantage of our film fabrication method is that it yields large-area crystalline films with a well-defined thickness and exceptionally low surface roughness, with an RMS value of 1.52 ML, as shown in Fig. 2c of the manuscript. A limitation of this approach, however, is that it does not allow for continuous film growth and simultaneous SHG characterization. Instead, each film thickness has to be grown separately under fully reproducible fabrication conditions. Because of this constraint, acquiring a large number of additional experimental data points becomes technically demanding and is beyond the scope of the present study.

In addition to the above changes to the figure, we have made the following changes to the manuscript:

Line 224:

While previous work has reported oscillations in SHG conversion efficiency η with film thicknesses [3,7], here we instead ~~fabricate and measure an integer number of layers, achieving~~ observe an increase without pronounced oscillations. This indicates that, under our film growth and measurement conditions, the SHG response is primarily governed by a non-oscillatory contribution that increases with decreasing thickness and dominates over oscillatory contributions related to QWS resonances or thickness-dependent phase interferences.

Also, we note that we did not claim SHG oscillations with thickness in the initial manuscript:

Line 75:

In particular, second-harmonic generation (SHG) has been predicted to oscillate as a function of the number of atomic layers [2, 3, 7].

1.4

> Please include error bars for the experimental results in Figure 4.

We included error bars in the revised Fig. 4.

1.5

> From the data shown in Figure 4b-d and in the SI, an increase in SHG power by ~ 35 can be deduced (for some y scales the ticks are missing). It is not clear how an increase of almost two orders of magnitude was obtained.

We apologize for the ambiguity. Nevertheless, we stand by our two-orders of magnitude claim. We have revised Fig. 4 and ensured consistent and appropriate y-axis scales and tick marks to make this clearer.

In particular, Figure 4b shows the SHG conversion efficiency η' on the y axis, normalized to the average powers as defined in the main text. Figure 4c shows the SHG conversion efficiency η, normalized to the peak intensities, which makes it directly comparable to values reported in the literature. This conversion efficiency η can further be normalized by dividing by the film thickness t and compared relative to the thickness-normalized conversion efficiency of the 30 ML thick film for each excitation frequency. This procedure yields a relative enhancement factor of 76 for an excitation wavelength of 1.8 μm in the 11 ML-thick film with respect to the 30 ML-thick film.

We have clarified this point more explicitly in the manuscript through the following changes:

> **Line 78:**
>
> In this work, we explore the strong thickness-dependent nonlinear response of atomically thin crystalline silver films to enhance the ~~SHG efficiency~~ thickness-normalized SHG conversion efficiency by almost two orders of magnitude.

> **Line 111:**
>
> Here, we demonstrate that QWS in long-term stable (compare SI Fig. S6) wafer-scale ultra-thin crystalline silver can enhance ~~SHG~~ thickness-normalized SHG conversion efficiencies by nearly two orders of magnitude while simultaneously reducing the interaction nonlinear volume.

> **Line 226:**
>
> At an excitation wavelength of $\lambda_0 = 1.8\,\mu$m, we achieve an ~~enhanced~~ enhancement of the thickness-normalized SHG conversion efficiency η/t by almost two orders of magnitude when ~~by~~ decreasing the film thickness ~~by a factor of ~ 3~~ from 30 ML to 11 ML.

1.6

> In Fig. 4a, it is not clear why the measurements were taken in the saturation regime of the detector, since it is possible to use density filters to reduce the detected power and compensate for this in post-processing analysis, which would benefit the interpretation of the results and the clarity of the presentation.

We fully agree with the Reviewer that data acquisition in the saturation regime of a detector is not necessary in the infrared and mid-infrared range due to the availability of suitable neutral density filters. For practical reasons, all data were acquired automatically at different sample positions, excitation powers, and wavelengths. Although a neutral density filter could in principle be used for higher excitation powers, the damage threshold of the sample lies close to the excitation levels required to reach detector saturation. Implementing an automated workflow for multiple high-power measurements under these constraints was therefore not pursued.

Nevertheless, we believe that the detector-saturation model, together with the number of data points provided for each data series in Fig. 4a, allows for the extraction of the estimated conversion efficiencies with very high accuracy.

Despite these considerations, our initial intention with Fig. 4a was to present the raw, unnormalized, and uncorrected data, so that different data series can be compared directly. This makes it immediately apparent that thinner films yield a stronger absolute SHG signal.

Nevertheless, to make it clear that we can work consistently in this regime, we have corrected all data for detector saturation and dark counts and now present the corrected data in Fig. 4a of the revised manuscript without these effects. This revised figure now shows the expected scaling. For transparency, we have moved the original version of Fig. 4a to the SI.

> **Line 196:**
>
> ~~At low excitation powers, the detected signal is primarily limited by dark counts, while at high powers, detector saturation becomes significant.~~ ~~In the intermediate regime, a~~ A quadratic dependence with a slope of 2 on a log–log scale is observed in Fig. 4a, confirming the second-order power scaling characteristic of the SHG process, while we corrected for saturation and background counts. As detailed in the Methods section, the regions affected by the detector nonlinearity can be well-modeled (see Methods and SI Fig. S5), enabling the extraction of the actual average SHG power P_{SHG} from the measured average detector power P_{det}.

1.7

> The manuscript would benefit from a broader discussion of the origin of the SHG signal, which in this case will arise primarily from surface effects (T. F. Heinz et al., Phys. Rev. Lett. 63, 644 (1989) [9]). In principle, SHG can be expected to arise from both the silicon-Ag and the Ag-air interface. Therefore, it would not be entirely counterintuitive to expect that reducing the film thickness could lead to an increase of the transmitted incident light and of the generated SHG light for the respective interfaces. Notably, the skin depth of light at 1-3 μm is about 25 nm, around only 3 times the studied film thickness and could thus be expected to impact the nonlinear signal generation and detection.

We thank the Reviewer for the opportunity to clarify the role of skin-depth effects and the physical origin of the observed SHG enhancement.

Finite optical penetration affects the absolute detected SHG power, in particular as the film thickness changes. However, within our theoretical formalism, its influence on the extraction of the nonlinear

susceptibility is negligible. In the determination of the effective $\chi^{(2)}$, absorption of the fundamental field at ω and absorption of the generated second-harmonic field at 2ω constitute competing effects that partially compensate each other. This balance is captured by the depth-averaged phase factors entering the nonlinear susceptibility derivation through

$$\left|\frac{1}{t}\int_0^t \mathrm{e}^{\mathrm{i}k_z z}\,dz\right|^2 \Big/ \left|\frac{1}{t}\int_0^t \mathrm{e}^{\mathrm{i}\tilde{k}_z z}\,dz\right| \approx 1, \tag{2}$$

where t is the thickness of the film, $k_z(\omega)$ the complex out-of-plane wave vector at the fundamental frequency, and $\tilde{k}_z(2\omega)$ the corresponding one at the SHG frequency. We show that, for the thicknesses and wavelengths considered here, absorption-induced attenuation does not significantly modify the effective nonlinear response obtained from the model. While absorption directly impacts the measured SHG intensity, the competing absorption of the pump and the generated fields cancel in the extraction of $\chi^{(2)}$, thereby justifying the use of an effective two-dimensional description. This point is now explicitly discussed in the revised Supplementary Information.

In ultra-thin films, a strict separation between surface and bulk contributions is not meaningful. Instead, the nonlinear response must be understood to arise from the entire film, whose electronic structure – and thus SHG efficiency – is strongly modified by thickness, dielectric environment, and interfacial symmetry breaking. As elaborated below, we now turn to the physical origin of the SHG signal and the respective roles of the Ag–Si and Ag–Air interfaces.

Building on this perspective, the SHG signal in our system originates from both intrinsic and extrinsic nonlinear sources. Intrinsically, SHG is generated at interfaces where inversion symmetry is broken, namely the air-Ag and Ag–Si interfaces. Because the optical field at the considered wavelengths penetrates through the entire film thickness, both interfaces contribute, as correctly pointed out by the Referee. In ultra-thin crystalline Ag, quantum confinement further enhances the intrinsic response: the parity, shape, and spacing of the quantized electronic subbands depend sensitively on the number of layers and their environment, giving rise to stronger anharmonic electron motion and enhanced surface susceptibilities. On the extrinsic side, thinner films exhibit reduced reabsorption of both the pump and the generated SHG, increasing the effective nonlinear interaction at both interfaces. Additionally, the linear optical response improves for thinner crystalline films—specifically, the plasmonic resonances acquire a higher quality factor, which leads to stronger local-field enhancement and thus further boosts the SHG efficiency. Combined, these intrinsic (interface symmetry breaking and quantum-well–induced enhancement) and extrinsic (improved transmission and increased field enhancement) mechanisms consistently reinforce each other, explaining the observed increase in SHG with decreasing film thickness.

To illustrate possible mechanisms that affect the electronic structure and, consequently, the nonlinear optical response of thin metal films, we investigate in Fig. R1 the effect of a silicon substrate on the electronic structure of the considered systems. To this end, using bulk atomic positions for both Si and Ag, we construct a heterostructure consisting of a Ag(111) film supported on a Si(111) substrate. No atomic relaxations are incorporated, allowing us to isolate symmetry- and hybridization-induced electronic effects without introducing additional structural degrees of freedom. To eliminate in-plane lattice-mismatch effects (the in-plane lattice constants of Ag(111) and Si(111) surfaces are 2.879 Å and 3.84 Å, respectively), six layers of a 4×4 Ag(111) supercell are placed on six layers of a 3×3 Si(111) surface (see Fig. R1(a)). This commensurate supercell construction minimizes artificial strain while preserving the essential symmetry-breaking effects induced by the substrate. In Fig. R1(b), we present the band structure of six monolayers of free-standing Ag(111), and compare it with the band structure of six monolayers of Ag supported on a six-layer Si substrate in Fig. R1(c). As observed in the figures, placing silver on silicon leads to several notable modifications in the electronic structure:

- **Substrate-induced symmetry breaking:** Degeneracies present in the free-standing Ag bands are lifted or modified, reflecting the reduced symmetry and the breaking of translational invariance induced by the substrate. This effect is clearly visible, for example, in the first occupied state near the Γ point, commonly referred to as the Shockley–Tamm surface state.
- **Interfacial hybridization effects:** Signatures of hybridization between Ag and Si electronic states emerge, manifested as band splittings and avoided crossings. These features indicate a non-negligible electronic coupling at the Ag/Si interface, particularly evident in the vicinity of the K point.

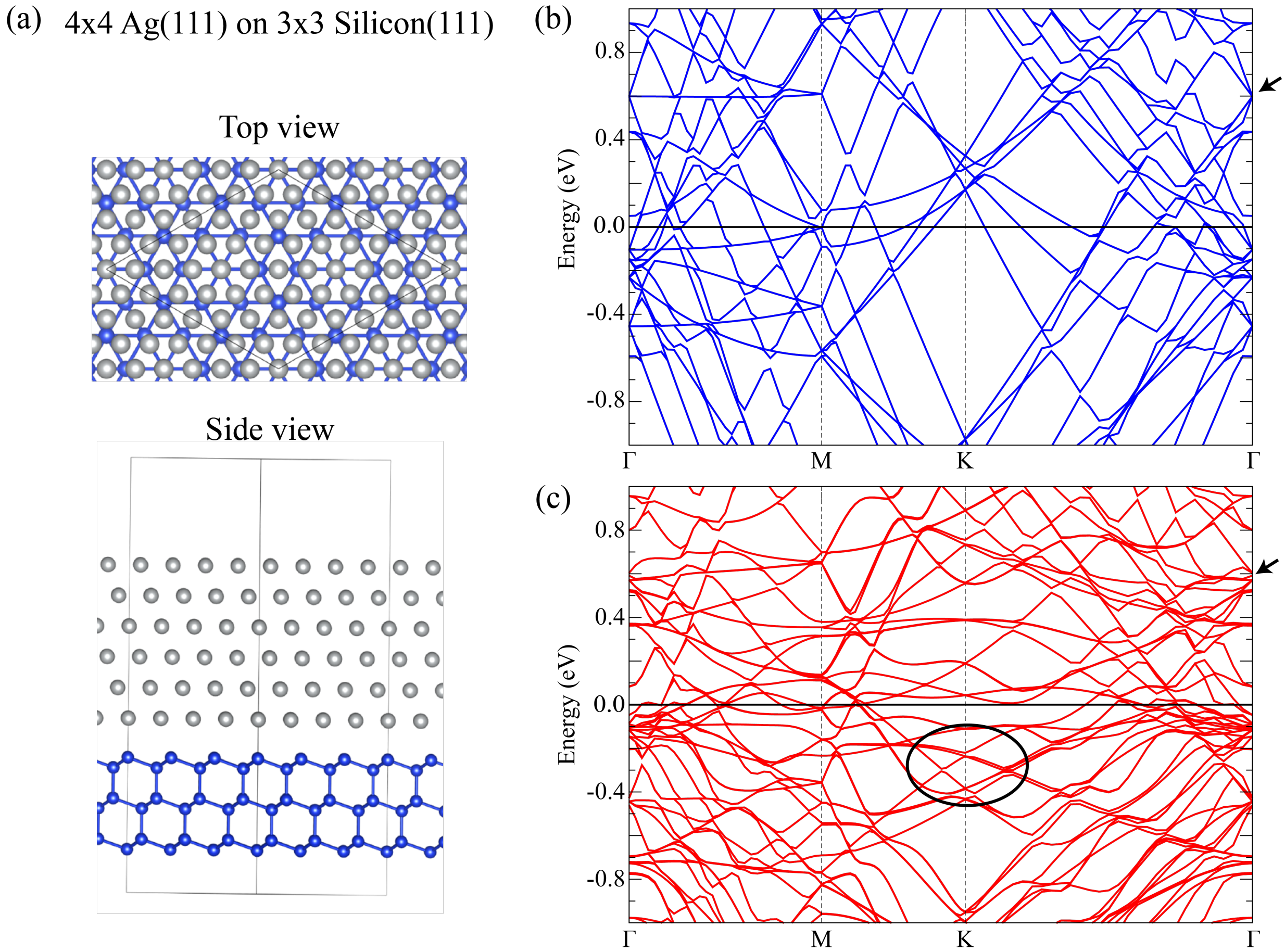

Figure R1: (a) Top and side views of the atomic structure of a six-layer 4×4 Ag(111) film on top of a six-layer 3×3 Si(111) substrate. (b,c) Electronic structure of the considered system (b) without and (c) with the silicon substrate. The circle highlights the electronic bands at the K-point below the Fermi level originating from the Ag-Si hybrid states. The black arrows on the right mark the degeneracy-breaking around the Γ-point due to the presence of the silicon substrate.

- **Charge redistribution and density-of-states enhancement:** The Ag/Si system exhibits an increased density of electronic states below the Fermi energy compared to free-standing Ag, suggesting charge redistribution and enhanced electronic occupation in this energy range due to interaction with the substrate, most prominently near the Γ point.

These modifications of the electronic structure arise directly from the presence of the Si(111) substrate, which breaks the symmetry of the otherwise free-standing Ag film and introduces interfacial electronic coupling. Such substrate-induced symmetry breaking and hybridization effects are expected to play a crucial role in determining symmetry-sensitive nonlinear optical responses, including second- and higher-order processes. Additionally, from the band-structure analysis, several thickness-dependent related effects can be identified:

- **Quantum confinement effects:** The discretization of electronic states due to finite thickness leads to discretization of conduction electrons in the out-of-plane direction (i.e., QWS), resulting in lifted degeneracies and modified band dispersions.
- **Reduced structural symmetry:** As the number of atomic layers decreases, it becomes increasingly difficult to preserve the symmetry of the atomic structure, especially in the presence of a substrate.
- **Enhanced surface sensitivity:** Thinner films exhibit a larger surface-to-volume ratio, making surface defects, imperfections, and interfacial effects more influential on the electronic properties.

- **Deviation from bulk-like behavior:** With decreasing thickness, the electronic bands become less parabolic (that is, more anharmonic), reflecting the breakdown of the bulk limit and the increasing importance of finite-size effects.

Under realistic experimental conditions, additional environmental factors, such as the presence of capping layers, unavoidable silicon surface reconstructions, or slight misorientation and imperfections in the ultra-thin Ag layers, can further modify the electronic structure. A comprehensive treatment of all these effects is not feasible for the mesoscopic systems considered here. Therefore, in our first-principles calculations, we deliberately focus on idealized, symmetric Ag(111) films to identify thickness-induced electronic effects on the nonlinear response of Ag thin layers. These additional calculations support the conclusion that extra symmetry-breaking mechanisms present under the actual experimental conditions contribute to the discrepancies between theoretical predictions and experimental observations. We clarified this point as follows:

> **Line 228:**
>
> ~~This provides~~This decrease in thickness produces a drastic boost in conversion efficiency, which is observed for the first time in this system, well beyond skin-depths effects, showing the potential of band structure engineering in this novel nanophotonic platform.

> **Line 251:**
>
> In ultra-thin metallic films, the electromagnetic field probes the entire Ag film thickness due to the finite optical penetration depth at the excitation wavelengths considered in this work. Therefore, the nonlinear response must be understood as arising from the full film, whose electronic structure is strongly modified by thickness, dielectric environment, and interfacial symmetry breaking. Intrinsically, SHG originates from regions where inversion symmetry is broken, namely at the Ag–air and Ag–Si interfaces, both of which contribute simultaneously to the measured signal. In the ultra-thin crystalline limit, quantum confinement further enhances the intrinsic nonlinear response by modifying the parity, spacing, and anharmonicity of the quantized electronic subbands, leading to enhanced effective surface nonlinear susceptibilities.
>
> At the same time, extrinsic mechanisms reinforce this enhancement: reducing the film thickness produces a decrease in reabsorption of both the fundamental and SHG fields and improves the linear optical response, including stronger local-field enhancement. While intrinsic mechanisms primarily modify the nonlinear susceptibility through symmetry breaking and quantum-well–induced electronic restructuring, extrinsic mechanisms act by enhancing local optical fields and the effective interaction length, thereby increasing the observable SHG yield without altering the microscopic origin of $\chi^{(2)}$. These intrinsic and extrinsic effects act cooperatively, providing a consistent physical picture for the observed increase of SHG efficiency with decreasing Ag film thickness.

2 Reviewer 2

We thank Reviewer 2 for the detailed review, which have helped us further strengthens the manuscript. Some of the comments overlap with points already raised by Reviewer 1, and therefore we refer to the corresponding sections above. In the following, we address the points raised by Reviewer 1 regarding the novelty of the work, the comparison with prior studies, the broader impact of this work, the polarization analysis, and the comparison between theory and experiment in the respective sections below.

2.1

> 1. Regarding Novelty and Positioning: The idea that QWS in Ag(111) affect SHG is not entirely new.
>
> The idea that quantum-well states (QWS) in ultra-thin Ag(111) films can influence second-harmonic generation (SHG) is well established, as demonstrated by Pedersen et al. (PRB 60, R13997 (1999)) [3] and Hirayama et al. (PRB 64, 195415 (2001)) [7], who reported oscillatory SHG variations with Ag thickness due to quantum confinement and parity alternation of electronic states. The present work differs mainly in its reported magnitude and monotonic trend, an enhancement of up to two orders of magnitude as thickness decreases from $\sim$ 30 ML to 10 ML. However, to convincingly establish novelty beyond prior QWS-based studies, the authors must clearly define what this enhancement is measured against and quantify the improvement relative to earlier results.

We thank the Reviewer for pointing out several aspects where our manuscript could be strengthened and clarified. We elaborate on the oscillations in Sec. 1.3, discuss the novelty of our study in Sec. 1.1, and highlight the corresponding changes made to the manuscript therein. The definition and discussion of the enhancement are clarified in Sec. 1.5. We also discuss the comparison to prior work in detail in Sec. 2.1.3.

2.1.1

> Is the two-order-of-magnitude increase calculated relative to bulk single-crystal Ag, to the thickest (30 ML) sample in the current series, or to previously reported Ag/Si(111) data from the literature?

It refers to the 30 ML thick film. We have clarified this point and made the wording more precise. A more detailed discussion can be found in Sec. 1.5.

2.1.2

> Are the conversion efficiencies normalized to interaction volume, illuminated area, or total film thickness?

The SHG conversion efficiencies are defined as $\eta = I_{\mathrm{SHG,peak}}/I^2_{0,\mathrm{peak}}$ analogous to the convention used in Ref. [3–6], where I_{peak} denotes the peak intensity within the silver film layer stack. Peak intensities take the illumination area into account (not the film thickness t) and can be directly compared to other works as discussed in Sec. 2.1.3. We clarified this point in the main text by making the following changes:

> **Line 205:**
>
> The SHG conversion efficiency normalized to the average power, $\eta' = P_{\mathrm{SHG}}/P_0^2$, is the single free parameter used to fit each power-dependence dataset in Fig. 4a and is presented in Fig. 4b for all excitation wavelengths. The SHG conversion efficiency η' for the full layer stack can be normalized to the peak intensity I_{peak} (see Methods for the conversion). It is defined as $\eta = I_{\mathrm{SHG,peak}}/I^2_{0,\mathrm{peak}}$ and is shown in Fig. 4c. ~~$\eta = I_{\mathrm{SHG,peak}}/I^2_{0,\mathrm{peak}}$, is the single free parameter used to fit each power dependence dataset in Fig. 4a. Here, I_{peak} denotes the peak intensity within the silver film, corrected for transmission losses in the setup. The results reveal that thinner silver films produce stronger absolute SHG signals than thicker ones. Applying the analogue analysis used in Fig. 4a to other excitation wavelengths, we obtain the SHG conversion efficiencies shown in Fig. 4b–d.~~ For all considered excitation wavelengths, we observe ~~a clear~~an increase in ~~absolute~~ SHG conversion efficiency η' with decreasing film thickness, even without any thickness or volume normalization.

Line 332:

$$P_{\text{SHG}} = \eta' P_0(\omega)^2, \tag{3}$$

Line 338:

The SHG conversion efficiency is ~~definded~~defined by $\eta = I_{\text{SHG,peak}}/I^2_{0,\text{peak}}$ and the peak intensity is given by $I_{\text{peak}} = \frac{f_{\text{shape}}}{f\tau A_{2\omega}} P_{\text{ave}}$, where ~~$A = \pi\sigma_0^2 \sec\theta_1$~~ $A_{2\omega} = \pi\left(\sigma_0/\sqrt{2}\right)^2 \sec\theta_0$ is the focal area~~ and~~, $f_{\text{shape}} = 94\%$ accounts for the Gaussian pulse shape, and we assumed a reduction of the beam waist by a factor of $\sqrt{2}$ for the SHG.

2.1.3

> How does the extracted $\chi^{(2)}$ (in pmV^{-1} or m^2V^{-1} units) compare to previously reported surface and interface nonlinear coefficients for Ag, Au, or Cu films of comparable thickness?

Optical damping in gold and copper is significantly stronger than in silver. A comparison of their nonlinear optical properties without taking their linear optical response into account would therefore be misleading, since gold and copper, due to their intrinsically higher imaginary part of the dielectric function, are less suitable for nanophotonic applications, and do not provide long plasmon lifetimes for further enhancement via plasmonic excitations.

Nevertheless, we have added a comparison of reported second-order nonlinear susceptibilities $\chi^{(2)}$ in ultra-thin silver films, as well as a comparison of second-harmonic conversion efficiencies η for silver films to the SI. The added tables can be found in Tables S3 and S4 of the SI.

2.1.4

> Furthermore, earlier studies reported oscillatory SHG behavior with thickness due to discrete subband transitions, whereas the present work claims a monotonic enhancement. Yet, Fig. 4 shows only five experimental points and theoretical curves that still exhibit oscillatory features, making the monotonic trend inconclusive. If this difference arises from improved crystallinity, reduced scattering, or better-defined QWS spacing, the authors should emphasize these aspects as the genuine source of novelty. It would also strengthen the case if the authors demonstrate that their approach allows:

Our experimental data consistently show that the SHG signal increases as the film becomes thinner. Possible oscillatory features in the experimental data remain speculative, since the sampling density in Fig. 4 is not sufficient to determine whether fine oscillations are present or not. Importantly, resolving such oscillations is not the goal of our work. Earlier studies focused on the study of the oscillations of the SHG signal as a function of the number of atomic layers [2,4] whereas here our objective is to demonstrate that the overall SHG efficiency is substantially enhanced as the thickness is reduced, as theoretically predicted [10]. We elaborate in more detail about this in the Sec. 1.3.

2.1.5

> Quantitative extraction of absolute $\chi^{(2)}$ values under well-calibrated excitation conditions;

Works such as Refs. [3,5,6] characterized the second-harmonic signal in reflection and used quartz crystals as reference materials to calibrate their measurement apparatus.

We would like to emphasize that our measurements are performed in transmission, and that a calibration using a different, well-established nonlinear crystal is technically not feasible in our setup. Due to the distinct linear optical properties and the substantially different thicknesses of such crystals, significant walk-off effects would occur. We observed that even a comparatively thin quartz crystal of $525\,\mu$m thickness introduces substantial realignment steps in our setup, rendering a reliable calibration based on this approach impractical.

Instead, we carefully characterized the excitation and detection beams as well as all optical components used in the experiment, and explicitly took the full sample layer stack, including its Fresnel coefficients, into account in order to estimate the absolute conversion efficiency of the silver film.

Based on this procedure, we estimated the experimental second-order nonlinear susceptibility $\chi^{(2)}$ values from the measured conversion efficiencies η and added them to Fig. 4d of the manuscript and compared them with the theory.

2.1.6

> Enhanced film stability and reproducibility due to their Si-capping strategy and epitaxial control; and

We thank the Reviewer for pointing out that our description of the capping layer was not sufficiently precise. The capping layer consists of a ~ 1 nm thick silicon protection layer, which oxidizes under ambient conditions to form silicon dioxide. We have implemented the following changes:

> **Line 153:**
>
> Finally, before removal from UHV, our silver films are coated with a thin (≈ 1 nm) silicon passivation layer [11,12], which oxidizes under ambient conditions to form silicon dioxide to ensure their stability under ambient conditions.

An experimental study of the influence of the silicon-dioxide capping layer on the nonlinear signal is challenging, because an uncapped silver film would oxidize immediately when exposed to air. The most practical experimental comparison is therefore to use a different capping material. We have investigated gold as an alternative capping layer. SHG is also observed from this layer stack, but the signal is reduced compared to the silicon-dioxide-capped samples, which we attribute to the higher optical damping in gold. A systematic investigation of different capping layers is beyond the scope of the present work.
We show in Fig. S6 of the Supplementary Information the long-term stability of the SHG signal, which also demonstrates the preserved film quality over more than a month.

We would like to highlight that we have studied the influence of the silicon capping layer on the silver film extensively in a previous study in Ref. [11], in particular in Fig. S1 of the SI of that reference.

2.1.7

> Direct correlation between ARPES-resolved QWS and nonlinear response, something rarely achieved in prior work.

We thank the Reviewer for acknowledging that a combination of ARPES-resolved QWS and a characterization of their nonlinear response has been rarely achieved. We have strengthened this point in the manuscript as follows:

> **Line 120:**
>
> We provide both extensive material characterizations and quantitative nonlinear-optical characterizations. This combination, together with a level of theory that goes beyond the phenomenological QWS model discussed in previous work [1, 3, 4, 7], is rarely achieved and enabling experimental access to second-order nonlinear susceptibilities in the mesoscopic regime of noble metals.

2.1.8

> SHG conversion efficiencies is plotted without error rate which is better to include so that the potential reader can also observe accuracy (and/or precision) of the reported data.

We have revised Fig. 4 and added error bars to all experimental data.

2.1.9

> How the structure planar orientation upon the measured quantities would get affect by this: polarization resolved/anisotropic SHG? It would be better show this as a systematic study. (I will lay this out further below.)

Our work focuses on the enhancement of the SHG signal achieved by decreasing the film thickness. A polarization-resolved or anisotropic SHG study on these films is certainly an interesting and valuable follow-up direction.
However, such a study is beyond the scope of the present work due to a combination of factors: the anisotropy of the host material, the incident angle being tilted with respect to the surface normal, and the fact that our measurements are performed in transmission in order to access the wavelength range relevant for plasmonic applications. We address these points in more detail in the corresponding Sec. 2.4.

2.1.10

> [1] Hirayama H, Komizo T, Kawata T, Takayanagi K. Optical second harmonic generation spectrum of Ag/Si (111) reconstructed surfaces. Physical Review B. 2001 Mar 29;63(15):155413. [7]
> [2] Chang, C. S., and J. T. Lue. "Optical second harmonic generation from thin silver films." Surface science 393.1-3 (1997): 231-239. [6]

We considered all references cited in the initial manuscript and have now included them in Table S3 of the SI, which presents a comparison of the SHG conversion efficiencies η with previously reported works.

2.2

> 2. Quantitative Comparison with Literature
>
> One of the central weakness of the current manuscript is the lack of quantitative benchmarking against prior experimental and theoretical studies of nonlinear optical response in ultra-thin metallic films. While the authors report a "two-order-of-magnitude enhancement" in SHG efficiency, no clear numerical comparison is provided with previous Ag(111) QWS sytems or other atomically thin plasmonic films. Without such contextualization, the reader cannot assess whether the observed enhancement is genuinely unprecedented or primarily a relative improvement within this specific dataset.
>
> Previous works—such as Chang & Lue, Surface Science 393, 231 (1997) [6]; Wong & Richmond, J. Chem. Phys. 99, 5500 (1993) [5]; and Pedersen et al., Phys. Rev. B 60, R13997 (1999) [3], reported $\chi^{(2)}$ coefficients for Ag thin films in the range of 10^{-21} to $10^{-20}\,\mathrm{m^2V^{-1}}$, depending on surface orientation and growth conditions. Even for nanostructured or resonantly enhanced Ag configurations, values rarely exceeded $\sim 10^{-19}\,\mathrm{m^2V^{-1}}$. The present manuscript, by contrast, implies $\chi^{(2)}$ values approaching or exceeding this upper bound, yet these numbers are not explicitly stated nor normalized for experimental parameters such as beam area, incidence angle, or local field corrections.
>
> To enable meaningful evaluation and reproducibility, let me kindly suggest authorsl

We have compared now the conversion efficiency η and the second-order nonlinear susceptibility $\chi^{(2)}$ to previously reported works in the tables S3 and S4 of the SI, respectively.

We want to emphasize that the works cited by the Reviewer, Refs. [3, 6], do not report second-order nonlinear susceptibilities. Both studies provide only reflection SHG conversion efficiencies η in units of $\mathrm{m^2/W}$, which were calibrated by quartz crystals with a well known second-order nonlinear susceptibility. In the comment, however, these values were mistakenly treated as if they were second-order nonlinear susceptibilities in units of $\mathrm{m^2/V}$. The work in Ref. [5] indeed reports both conversion efficiencies and second-order nonlinear susceptibilities. However, the units and numerical ranges provided by the Reviewer do not match the actual values of the publication, which reports conversion efficiencies on the order of $\eta \sim 10^{-23}\,\mathrm{m^2/W}$ when converting to SI units. We also want to stress that all three works mentioned by the Reviewer [3, 5, 6] were already included in our initial manuscript.

The last value provided by the Reviewer appears without reference or context. We therefore cannot address this comment, since we cannot verify whether the correct units were used. In addition, resonantly enhanced nanostructured silver configurations are beyond the scope of this study. We believe a misunderstanding occurred: conversion efficiencies are inverse intensities, as defined in our manuscript and in Refs. [3,5,6], and are by definition normalized to the beam area while also accounting for the incident angle.

We would like to emphasize that we have neither claimed nor implied that we exceed any upper bound of the second-order nonlinear susceptibility. All incident angles, normalizations, and field corrections are stated explicitly and treated transparently throughout the manuscript. We have further clarified these normalizations throughout the text.

In addition, we now compare our values to previously reported work, as discussed in the main text, as follows:

> **Line 216:**
>
> We observe conversion efficiencies η that are of the same order of magnitude as those reported in a previous work [6], which does not report an increase for thinner films due to the use of evaporated, non-crystalline films. In contrast, smaller conversion efficiencies are observed in a separate work [3], which focuses on crystalline films. We summarize this comparison in SI Tab. S3.

2.2.1

> Tabulating experimental $\chi^{(2)}$ values (in m^2V^{-1} or pmV^{-1}) extracted for each thickness and excitation wavelength, accompanied by estimated uncertainties and calibration methodology.

We show all experimental $\chi^{(2)}$ values together with their uncertainties in Fig. 4d as a function of film thickness and excitation wavelength; tabulating them again in the main text would therefore be redundant. We elaborate on the calibration method used in Sec. 2.1.5 and have implemented the corresponding changes in Sec. 2.1.2 and Sec. 1.1, which allow the experimental $\chi^{(2)}$ values shown in Fig. 4d to be derived. In addition, we have made the following further modifications to make our analysis more transparent:

> **Line 330:**
>
> The average SHG power, P_{SHG}, was related to the average excitation power P_0 by assuming a second-order power law:
>
> $$P_{\text{SHG}} = \eta' P_0(\omega)^2, \quad (4)$$
>
> where η' is the fitted conversion efficiency extracted from Fig. 4a and shown in Fig. 4b.

2.2.2

> Including a comparative summary table listing literature-reported $\chi^{(2)}$ values for Ag(111), (relevant nanostructured films and even howabout Au(111), Cu(111)?), clearly identifying whether the values correspond to bulk, surface, or interface contributions.

We elaborated on the comparison to gold and copper films in Sec. 2.1.3, and added a discussion on literature-reported second-order nonlinear susceptibility values in Sec. 2.1.3. A distinction between surface, bulk, and interface contributions in the mesoscopic regime of ultra-thin films is, in our perspective, not applicable in this context, as mentioned in Sec. 1.7. Nanostructured films or nanoparticles are certainly promising platforms for nonlinear nanophotonic applications. However, due to the presence of plasmonic resonances, we believe that a direct comparison between bare films and such structured metals is not meaningful.

2.2.3

> Differentiating between absolute and relative enhancements: For instance, whether the “two orders of magnitude” refers to integrated SHG power, peak intensity, or normalized susceptibility.

We clarified the statement “two orders of magnitude” in Sec. 1.5. We carefully reviewed the manuscript to ensure that it is clear to the reader whether we refer to an enhancement in general, a relative enhancement, or an absolute enhancement when reporting values. We are aware that any enhancement must always be defined with respect to a specific reference, which must be clear from the context or explicitly stated.

2.2.4

> Clarifying the normalization procedure used in Figs. 4b–d: are the data corrected for optical throughput, detector response, and Fresnel transmission across the Si substrate and capping layer?

We would like to emphasize that we have revised Fig. 4. We have modified both the main text and the Methods section to clarify the normalization procedure. The individual changes are discussed in more detail in Sec. 2.1.2, Sec. 2.1.5, and Sec. 1.1.

2.2.5

> Additionally, the theoretical predictions in Fig. 4e yield $\chi^{(2)}$ values of comparable magnitude but display different wavelength dependence. Quantitative comparison of these results with experimental data, possibly through a combined plot of experiment vs. theory vs. literature, would significantly strengthen the manuscript's credibility and reveal how the proposed model extends or refines earlier phenomenological treatments.

We now compare the experimental and theoretical values of the second-order nonlinear susceptibility in a single, common plot shown in Fig. 4d, and compare to literature in Sec. 2.1.3.

> **Line 244:**
>
> We compare the experimental and theoretical ~~SHG conversion efficiencies η in Fig. 4b-d~~ second-order nonlinear susceptibility $\left|\chi_b^{(2)}\right|$ in Fig. 4d for the considered excitation wavelengths of 1.8, 2.3, and 3.1 μm.

2.2.6

> Finally, because the work positions itself as a quantum-engineered platform rather than a purely plasmonic one, it is critical to emphasize the degree of enhancement relative to previously reported QWS oscillatory behavior. The authors might show that while earlier studies achieved modest modulation amplitudes ($\leq 10\times$), the present atomically controlled films exhibit a monotonic, $100\times - 200\times$ increase with superior long-term stability, thus establishing a genuine quantitative leap.

Indeed, we report a significant increase in the SHG signal upon decreasing the film thickness. Our films exhibit long-term stability and constitute a robust plasmonic platform. A direct comparison of the SHG modulation with other works does not appear appropriate, as the reported results depend strongly on the excitation wavelength, film quality, and the characterization and calibration methods employed. A simple comparison that does not account for these differences would therefore be potentially misleading.

2.2.7

> Figure 2 effectively demonstrates structural and electronic order, validating sample quality, but its analysis could be expanded with quantitative ARPES interpretation (e.g., QWS energy spacing vs. thickness) and correlation with nonlinear measurements for a stronger mechanistic connection.

We thank Reviewer 2 for pointing out that Fig. 2 demonstrates the structural order and validates the sample quality. Yes, energy distribution curve (EDC) plots can be extracted from the ARPES measurements, as shown in the SI Fig. S2. Modeling the EDCs would straightforwardly reveal that thinner films exhibit larger QWS spacings. However, a quantitative modeling of the EDCs depends on a variety of parameters and assumptions and would not provide additional insight beyond this global trend. From our perspective, such a modeling effort would therefore not add new meaningful information, as it would merely reproduce the known result that thinner films show increased QWS spacing, as reported also in an earlier work of us [11].

2.2.8

> In summary, the manuscript will benefit from a clearer articulation of how its findings extend beyond the foundational studies of QWS-mediated SHG on whether through improved experimental precision, expanded wavelength coverage, quantitative susceptibility extraction, or long-term stability of atomically thin metallic films. A concise comparison table summarizing literature $\chi^{(2)}$ values and enhancement factors would make this distinction unambiguous. The claim of "two orders of magnitude enhancement" needs explicit normalization (e.g., relative to bulk Ag or to previous QWS films). Please check the following works including but not limited to (as there are several others..)

All mentioned points here relate to aspects that are addressed in more detail elsewhere in this reply letter. We therefore refer the Reviewer to the respective dedicated sections. More precisely, how our study goes beyond existing QWS work is discussed in Sec. 1.1. The tabulated values can be found in Sec. 2.1.3, and enhancement factors found in different works, including our present study, are discussed in Sec. 1.5. We have emphasized the long-term crystalline stability more clearly:

> **Line 122:**
>
> In our thin-crystalline-metal platform, with long-term crystalline stability, light-matter interaction could be further increased by introducing nanostructures for local field enhancement [13–15], offering a new route for highly efficient nonlinear nanophotonics and plasmonics [10].

2.2.9

> [1] Pedersen, T.G., Pedersen, K. and Kristensen, T.B., 1999. Optical second-harmonic generation from Ag quantum wells on Si (111) 7× 7: experiment and theory. Physical Review B, 60(20), p.R13997. [3]
> [2] Chang, C.S. and Lue, J.T., 1997. Optical second harmonic generation from thin silver films. Surface science, 393(1-3), pp.231-239. [6]
> [3] Wong, E.K.L. and Richmond, G.L., 1993. Examination of the surface second harmonic response from noble metal surfaces at infrared wavelengths. The Journal of chemical physics, 99(7), pp.5500-5507. [5]

All references were recognized in the initial version of our manuscript. We carefully went through the entire manuscript again and added them wherever applicable. All relevant works are now acknowledged in the tables comparing conversion efficiencies and second-order nonlinearities with related studies, which can be found in Tables S3 and S4 of the SI.

2.3

> 3. Broader Impact and Missing Perspective
>
> While the manuscript establishes a clear observation of enhanced nonlinear response in atomically thin silver films, it does not sufficiently articulate the broader scientific and technological context where such an increase in $\chi^{(2)}$ can drive new physics or device concepts. From aspect of potential readers, the discussion should go beyond immediate SHG efficiency gains and convey why these materials are relevant to next-generation photonic systems.
>
> Specifically, the authors could enrich the Discussion by addressing how the demonstrated large nonlinearities may interface with ongoing advances in:

We thank Reviewer 2 for acknowledging that our study demonstrates a clearly enhanced nonlinear response in the mesoscopic regime as the film thickness decreases. We want to highlight that the most promising application we see lies in enhanced light-matter interactions on the nano scale. We stated this in the following changes throughout the manuscript:

> **Line 122:**
>
> In our thin-crystalline-metal platform, light-matter interaction could be further increased by introducing nanostructures for local field enhancement, [13–15] offering a new route for highly efficient nonlinear nanophotonics and plasmonics [10], including dynamically tunable and reconfigurable nonlinear plasmonic systems that enable active control over harmonic generation [16].

> **Line 283:**
>
> Plasmon resonances, nanostructures, and electric field enhancements have the potential to boost the conversion efficiency to reach nonlinear strengths comparable to those of 2D materials while maintaining electronic tunability [17–19].

2.3.1

> Tunable and reconfigurable nonlinear systems – e.g., electrical, optical, or strain-based modulation of QWS to dynamically control harmonic generation.

We have added a reference addressing tunable and reconfigurable nonlinear systems that allow for dynamic control of harmonic generation as a further perspective.

> **Line 122:**
>
> In our thin-crystalline-metal platform, light-matter interaction could be further increased by introducing nanostructures for local field enhancement, [13–15] offering a new route for highly efficient nonlinear nanophotonics and plasmonics [10], including dynamically tunable and reconfigurable nonlinear plasmonic systems that enable active control over harmonic generation [16].

2.3.2

> Chiral and nonreciprocal photonics: since the broken inversion symmetry at the Ag/Si interface and the anisotropic QWS dispersion could, in principle, enable circular dichroism or nonlinear optical isolation effects when combined with magnetic or spin–orbit perturbations.

We thank the Reviewer for pointing our attention to works related to this topic.

Chiral and nonreciprocal photonics are outside the scope of this study. Our aim is to establish a new platform for metal-based plasmonics and nanophotonics in the infrared and mid-infrared regimes, which can benefit from increased nonlinearities as the interacting volume decreases.

2.3.3

> Hyperbolic and plasmonic metamaterials: integration of these atomically thin metals into multilayer stacks or metasurfaces could yield broadband enhancement and phase-engineered nonlinear responses.

We thank the Reviewer for pointing our attention to this topic. We believe that this consideration of plasmonic metamaterials and metasurfaces applies to nonlinear noble metals in general and is not restricted to ultra-thin silver films. We therefore do not believe that highlighting this point would strengthen the present manuscript. Also, due to the stringent requirements on the silicon substrate, the growth of crystalline silver films in multilayer stacks is unfortunately not technically feasible at present.

2.3.4

> Quantum and single-photon nonlinearities: the strong field confinement and quantum-level anharmonicity might offer a route toward nonlinear photon–electron interactions in mesoscopic cavities or quantum emitter. . .

We would like to emphasize that our platform is proposed for nonlinear nanophotonics. Single-photon nonlinearities require extremely strong nonlinear materials or very strong electric-field confinement. We do not believe that our platform is suitable for reaching the single-photon nonlinear regime with mesoscopic band structure engineering. We are confident that other nonlinear platforms are better suited for single-photon nonlinearities. Covering these topics in a dedicated review paper could certainly add value, but we recognize that this is beyond the scope of the present work.

2.3.5

> [1] Bhowmik, G., An, Y.Q., Schujman, S., Diebold, A.C. and Huang, M., 2020. Optical second harmonic generation from silicon (100) crystals with process tailored surface and embedded silver nanostructures for silicon nonlinear nanophotonics. Journal of Applied Physics, 128(16). [20]
> [2] Ohnoutek, L., Olohan, B.J., Jones, R.R., Zheng, X., Jeong, H.H. and Valev, V.K., 2022. Second harmonic Rayleigh scattering optical activity of single Ag nanohelices in a liquid. Nanoscale, 14(10), pp.3888-3898. [21]
> [3] Olohan, B.J., Petronijevic, E., Kilic, U., Wimer, S., Hilfiker, M., Schubert, M., Argyropoulos, C., Schubert, E., Clowes, S.R., Pantoş, G.D. and Andrews, D.L., 2024. Chiroptical second-harmonic

> tyndall scattering from silicon nanohelices. ACS nano, 18(26), pp.16766-16775. [22]
> [4] Kilic, U., Hilfiker, M., Ruder, A., Feder, R., Schubert, E., Schubert, M. and Argyropoulos, C., 2021. Broadband enhanced chirality with tunable response in hybrid plasmonic helical metamaterials. Advanced Functional Materials, 31(20), p.2010329. [23]
> [5] Kolmychek, I.A., Malysheva, I.V., Novikov, V.B., Leontiev, A.P., Napolskii, K.S. and Murzina, T.V., 2020. Phase-matched optical second harmonic generation in a hyperbolic metamaterial based on silver nanorods. Physical Review B, 102(24), p.241405. [24]
> [6] Dickinson, T., Afxenti, I., Astrauskaite, G., Hirsch, L., Nerenberg, S., Jedrkiewicz, O., Faccio, D., Müllenbroich, C., Gatti, A., Clerici, M. and Caspani, L., 2025. Quantum-enhanced second harmonic generation beyond the photon pairs regime. Science Advances, 11(27), p.eadw4820. [25]

We have carefully revised all references provided by the Reviewer 2 and included those that, in our assessment, add value to and strengthen the manuscript.

2.4

> 4. Incomplete Polarization and Symmetry Analysis
>
> While the manuscript provides a clear observation of second-harmonic generation (SHG) as a function of Ag film thickness, the polarization-resolved analysis is notably limited. All reported measurements are performed in the p→p configuration, with the authors noting that excitation through the Si substrate preserves only p-polarization. However, for a material system whose nonlinear response arises primarily from surface and interface symmetry breaking, such a restriction precludes a full understanding of the tensorial structure of $\chi^{(2)}$ and the associated selection rules governing the observed enhancement. In centrosymmetric metals like silver, the bulk electric-dipole contribution to SHG is forbidden under the electric-dipole approximation, and the observed signal therefore originates from:
>
> - Surface dipole contributions (due to inversion symmetry breaking at the vacuum–metal interface),
> - Interface contributions (arising at the metal–substrate boundary, particularly at Ag/Si(111)), and
> - Field-induced multipole or magnetic-dipole terms that become significant in ultra-thin or confined geometries.
>
> Without polarization-resolved SHG or azimuthal angle-dependent measurements, it is difficult to distinguish among these possible contributions. The resulting interpretation that the enhancement stems from solely from quantum-well-state (QWS) confinement and anharmonic electron motion, remains plausible but not conclusively demonstrated. To strengthen this section, the authors should consider or at least discuss the following:
> Perform or simulate polarization-dependent SHG (e.g., p→p, p→s, s→p) to extract effective tensor components (χ_{zzz}, χ_{xxz}, χ_{xzx}, etc.) and confirm whether the response is dominated by surface, interface, or field-induced contributions.

We agree with the Reviewer that bulk electric dipole contributions are forbidden in centrosymmetric materials. We would like to emphasize, however, that in the mesoscopic regime it is not possible to clearly distinguish between surface, interface, and bulk contributions in a way that allows for a direct comparison to their bulk counterparts. Instead, the entire film has to be treated as a nonlinear optical medium whose properties arise from modifications of the electronic band structure due to spatial confinement. In this regime, unambiguously identifying individual contributions from surfaces or interfaces is therefore not

meaningful and, moreover, would change with film thickness. For centrosymmetric metals, determining individual tensor components requires either (i) a detailed microscopic model that includes all symmetry-breaking mechanisms at both interfaces, or (ii) extensive polarization-dependent experimental datasets to serve as input. Such modeling becomes highly nontrivial in ultra-thin films, where substrate-induced symmetry breaking, quantum-well states, and thickness-dependent band-structure modifications contribute simultaneously.

We hope that the Reviewer understands the tremendous effort that would be required to perform DFT calculations for multiple film thicknesses and to model the individual thickness dependent $\chi^{(2)}$ tensor elements in this quasi-two-dimensional system. Such an analysis is far beyond the scope of the present work and would require several dedicated follow-up studies. However, we follow the suggestion of Reviewer 2 and clarify the SHG contributions for the polarization channels p $\rightarrow$ p, p $\rightarrow$ s, and s $\rightarrow$ p. We have characterized the p $\rightarrow$ p and p $\rightarrow$ s contributions in Fig. 3d, using p-polarized excitation light.

We previously stated that the p-polarized excitation light is largely preserved upon transmission through the substrate. To support this, we have added a new figure to the SI demonstrating this preservation, revealing a Jones vector close to $J_\mathrm{p} = (1,\ 0)$, corresponding to p-polarized (horizontal) polarized light, as shown in Fig. S15a. In contrast, Fig. S15b shows that s-polarized (vertical) light is not preserved; only about 0.66 of the initial light intensity entering the substrate remains s-polarized. We can confirm that, when exciting the sample with *s*-polarized light, which is not fully preserved upon transmission through the substrate, the SHG signal decreases. A residual signal remains due to the incomplete preservation of the excitation polarization in the substrate.

We have added the following text to the main text of the manuscript:

> **Line 189:**
>
> A purely s-polarized excitation cannot be achieved because p-polarized components remain after transmitting through the substrate (characterized in SI Fig. S15), and the resulting SHG signal is reduced compared to the p-polarized excitation.

2.4.1

> Include an analysis of symmetry breaking at the Ag/Si(111) interface, particularly since the Si(111) substrate exhibits surface reconstructions that inherently lack inversion symmetry. This could contribute significantly to $\chi^{(2)}$ and might even dominate the signal for the thinnest films.

We agree with the Referee that surface reconstruction can, in principle, lead to SHG contributions. However, contributions such as those discussed in Ref. [26] from the silicon substrate or a reconstructed silicon surface without deposited silver can be excluded based on the reference measurements shown in Fig. 3b of the manuscript. We are aware that the deposition of silver on the silicon substrate may itself induce a reconstruction at the silicon–silver interface. For practical experimental reasons, this effect is unavoidable and cannot be straightforwardly isolated or accounted for experimentally.

To assess the potential impact of such effects, we have discussed in Sec. 1.7 the impact of symmetry breaking at the silver interfaces.

2.4.2

> Given that the Ag/Si(111) interface naturally breaks inversion symmetry and could induce spin–orbit coupling effects, have the authors considered whether the observed enhancement might partially stem from interfacial or topological-like electronic states that contribute to $\chi^{(2)}$? In particular, could hybridized QWS–interface modes enable nontrivial charge or mode coupling across the Ag/Si boundary? If so, a short discussion on this would actually strengthen the manuscript's broader impact, showing awareness of emerging quantum–topological photonics connections.

We thank the Reviewer for this constructive suggestion. We agree that the broken inversion symmetry at the metal and substrate interface is a prerequisite for a nonvanishing second-order response in centrosymmetric metals, for which $\chi^{(2)}$ mainly arises from surfaces and interfaces. In addition to the QWS picture emphasized here, interface-specific electronic states could in principle contribute by renormalizing dipole matrix elements or enabling additional coupling channels across this boundary. For the (111) orientation, such states are expected to mainly affect the SHG response at lower excitation energies [10]. While our data show a pronounced thickness dependence consistent with QWS-driven effects, a detailed microscopic treatment of interface-specific or topological-like contributions is beyond the scope of this work.

2.5

> 5. Comparison Between Experiment and Theory plus Dielectric Function Analysis
>
> The inclusion of a quantum-mechanical model is conceptually strong, but the comparison between experiment and theory remains qualitative and idealized. The model assumes symmetric confinement and a free-electron–like response, neglecting the actual dielectric function $\varepsilon(\omega)$ of the Ag/Si(111) heterostructure. Since SHG efficiency depends critically on local field distributions governed by $\varepsilon(\omega)$, this simplification limits the quantitative validity of the predicted $\chi^{(2)}$.
>
> In ultra-thin epitaxial films, the dielectric function can deviate significantly from bulk Ag due to quantum confinement, substrate coupling, and surface or capping-induced asymmetry. Incorporating realistic dielectric dispersion, either from spectroscopic ellipsometry measurements or from ab initio optical-response calculations would make the modeling far more physically grounded. Ellipsometry-based retrieval of $\varepsilon(\omega)$ as a function of thickness could also reveal anisotropic optical behavior, which would independently support or challenge the polarization dependence inferred from SHG measurements. Such dielectric-function measurements would also establish a link between optical anisotropy and the tensorial components of $\chi^{(2)}$, complementing polarization-resolved SHG data.

We thank the Referee for this valuable suggestion. We agree that incorporating dielectric-function measurements is, in general, an excellent idea, and we note that polarization-resolved SHG measurements—which directly probe symmetry and tensorial components—are already presented and discussed in Sec. 2.4, together with the new Fig. S15. Moreover, the extraction of $\chi^{(2)}$ from experiment relies on directly measured SHG efficiencies (see Eq. 1 of the revised manuscript). As a result, the absolute magnitude of $\varepsilon(\omega)$ does not affect the validity of the extracted nonlinear susceptibility, while polarization-dependent effects—most sensitive to symmetry and anisotropy—are already experimentally addressed.

For films only a few monolayers thick, $\varepsilon(\omega)$ becomes a macroscopic *effective* parameter that does not capture the discrete electronic structure of ultra-thin Ag. Ellipsometry would therefore retrieve an effective-medium response rather than the microscopic behavior of quantized quantum-well (QW) states. In contrast, our theoretical framework is based on a semi-classical RPA model using DFT-derived effective masses, which explicitly incorporates quantum confinement and thickness-dependent subband structure. This approach allows us to reproduce the salient features of the experimentally observed band dispersions and their evolution with thickness, providing a physically grounded comparison between theory and experiment that is not accessible in classical or electromagnetic simulations based solely on $\varepsilon(\omega)$.

In Section *Theoretical model and origin of SHG* of the revised manuscript, we have strengthened the

comparison between experiments and theory, and commented in the revised Supplementary Information Sec. S1 on the linear fields.

> **Line 232:**
>
> Theoretical model and origin of SHG

2.5.1

> Additionally, the mismatch in wavelength dependence between measured and simulated $\chi^{(2)}$, currently attributed to "thermal broadening," remains speculative. A brief parameter-sensitivity analysis exploring how $\chi^{(2)}$ varies with damping, dielectric anisotropy, or surface asymmetry would clarify whether the model can reproduce experimental trends. Acknowledging these limitations and discussing potential extensions toward dielectric-function-informed modeling would substantially strengthen the theoretical interpretation.

Heat distribution upon illumination in ultra-fast optics is a separate study that requires a different approach. As discussed in Ref. [8], an extra ingredient is added when the film thickness is of the order of the nanometer, so bulk modeling no longer applies (e.g., the diffusion equation in the two-temperature model) and quantum finite size effects start to play a role. However, in simple terms, we can follow the prescription of Ref. [8], where we model the temperature-dependent dielectric response of silver using a Drude form with a temperature-dependent damping rate γ^T,

$$\epsilon_{\mathrm{Ag}}(\omega, T, T_l) = \epsilon_b - \frac{\omega_p^2}{\omega\,(\omega + \mathrm{i}\gamma^T)}. \tag{17}$$

Here the background permittivity $\epsilon_b = 4.0$ accounts for screening from interband transitions at the excitation wavelength $\lambda_{\mathrm{in}} = 1.4 - 3.1$ nm. The bulk plasma frequency is taken as $\hbar\omega_p = 9.17$ eV. The phenomenological damping rate is decomposed as $\gamma^T = \gamma^{e\text{-}e}(T) + \gamma^{e\text{-}ph}(T_l)$, which includes contributions from electron–electron and electron–phonon scattering processes, given by

$$\gamma^{e\text{-}e}(T) = \frac{\pi^3\alpha\beta}{12\hbar E_F} k_B^2 T^2 \quad \text{and} \quad \gamma^{e\text{-}ph}(T_l) \approx \gamma_0 \frac{T_l}{\theta_D}. \tag{5}$$

For silver, we use $\alpha = 0.55$ and $\beta = 0.73$ [27], a Fermi energy $E_F = 5.53$ eV [28], and a Debye temperature $\theta_D = 215$ K [29]. The inelastic broadening parameter is $\hbar\gamma_0 = 15$ meV, resulting in a room-temperature damping $\hbar\gamma(T_0, T_0) \approx 21$ meV, consistent with experimentally reported optical linewidths of silver in the near-infrared regime. An important consideration is that absorption at the wavelength of interest is of the order of $\mathrm{Im}\{\epsilon_{\mathrm{Ag}}\} \sim 10$, obtained from measured optical data [30].

In addition, the peak pump intensity and fluence estimated from the average incident power, assuming Gaussian temporal and spatial profiles at focus, is given by $I_{\mathrm{peak}} = f_{\mathrm{shape}}/f\,\tau\,A\,P_{\mathrm{ave}}$, where P_{ave} is the average pump power, f the repetition rate, τ the pulse duration, and $f_{\mathrm{shape}} = 0.94$ accounts for the Gaussian pulse shape. The effective focal area is $A = \pi\sigma_0^2 \sec\theta_0$, with σ_0 the beam waist and θ_0 the incidence angle inside the substrate. The peak fluence follows as $F_{\mathrm{peak}} = I_{\mathrm{peak}}\tau$. For $P_{\mathrm{ave}} = 5$ mW, $\tau = 200$ fs, $f = 76$ MHz, $\sigma_0 = 17$ μm, and $\theta_1 = 45°$, we obtain $I_{\mathrm{peak}} \approx 24$ MW/cm^2 and $F_{\mathrm{peak}} \approx 4.8$ μJ/cm^2. A poor-man estimate of the increase of temperature of the electrons is $\Delta T \sim F_{\mathrm{peak}}(\omega/c)\mathrm{Im}\{\epsilon_{\mathrm{Ag}}\}/Ce$, where $Ce \sim 10^5$ Jm^{-3}K^{-1} is the heat capacity of silver [31], so that we obtain $\Delta T \sim 10^1 - 10^2$ K. While such a modest temperature increase leads to only minor corrections to $\gamma^T = \gamma^{e\text{-}e} + \gamma^{e\text{-}ph}$ in Eq. (5) and is therefore not expected to be a leading contribution, elevated electronic temperatures can still modify state occupancies near the Fermi level and subtly influence the nonlinear response. Besides, note that the relaxation time to thermalize (∼ps) is much shorter than the time between pulses (∼ 0.1 ns).

3 Reviewer 3

> The manuscript “Atomically thin silver films for enhanced nanoscale nonlinear optics” by Philipp K. Jenke, et al. (2025) investigates how crystalline ultra-thin silver films, passivated with silicon, address the challenge of the inherently weak nonlinear optical response in bulk materials. Specifically, the study demonstrates an enhancement of the second-harmonic generation (SHG) as the film thickness decreases, attributed to modifications in the electronic band structure compared to bulk silver. The authors prepared the films in an ultra-high vacuum chamber ($1.0 \cdot 10^{-10}$ mbar) using electron-beam evaporation and characterized them with techniques such as low-energy electron diffraction (LEED), angle-resolved photoemission spectroscopy (ARPES), scanning tunneling microscopy (STM), X-ray photoelectron spectroscopy (XPS), and scanning electron microscopy (SEM). SHG measurements were performed with a modified focus-scan optical transmission setup using femtosecond laser pulses (~ 200 fs), while density functional theory (DFT) calculations were conducted with VASP simulation package.
>
> The study reveals a strong thickness-dependent nonlinear optical response in atomically thin Ag films, showing that SHG efficiency increases by nearly two orders of magnitude as the thickness decreases. The authors attribute this enhancement to the formation of quantum-well states in the confined electronic structure. The paper provides a clear description of the optical setup, particularly regarding the sample orientation to minimize SHG contributions from the Si (111) substrate. However, the rationale for selecting a 45° incidence angle could have been discussed in greater detail. The comparison between experimental and theoretical SHG efficiency is well structured, highlighting both the agreements and discrepancies between the results. Nevertheless, it would have been useful to reference and contrast these findings with those from “Second-Harmonic Generation Spectroscopy on Thin Films on Si (111) by Kjeld Pedersen” [4], especially the section concerning Ag/Si(111) $- 7 \times 7$. Finally, a brief discussion of how the silica capping layer influences (or does not influence) the SHG response would strengthen the analysis, considering that even though bulk SiO_2 is non-centrosymmetric, surface symmetry breaking can induce SHG [26] [32].
>
> Overall, this paper provides valuable insight into how quantum confinement in ultra-thin metal films alters their electronic structure and enhances nonlinear optical responses, contributing to the development of advanced nanophotonic and nonlinear optical materials.

We are delighted that Reviewer 3 agrees with our perspective that our work “provides valuable insight into how quantum confinement in ultra-thin metal films alters their electronic structure and enhances nonlinear optical response.”

Nevertheless, we would like to address the three points raised by the Reviewer.

3.1

> However, the rationale for selecting a 45° incidence angle could have been discussed in greater detail.

A sharper incident angle at the air–silicon interface would translate into a sharper incident angle at the silicon–silver interface, thus leading to stronger symmetry breaking. Such steep incident angles, however, were technically not achievable due to the limited space between the focusing and collection objectives. Increasing the angle further would have introduced significant shifts of the sample along the optical axis when keeping it in focus while scanning different lateral positions on the film. The maximum angle that could be achieved with our setup was therefore 45°.
Accordingly, we have added the following changes to the manuscript:

Line 166:

The sample was tilted to break the symmetry of the crystal structure and enable SHG, while the excitation angle was technically limited.

Line 317:

Most of the nonlinear emission was generated at the ~~focus~~focal plane, where fluence was maximum, refracting the technical-limited incident angle from $\theta_0 = 45\,^\circ$ in free space to $\theta_1 \approx 12\,^\circ$ inside the silicon substrate.

3.2

Finally, a brief discussion of how the silica capping layer influences (or does not influence) the SHG response would strengthen the analysis, considering that even though bulk SiO_2 is non-centrosymmetric, surface symmetry breaking can induce SHG [26] [32].

An experimental study of the influence of the silica capping layer on the silver film is done in our previous study in Ref. [11].

A contribution such as that discussed in Ref. [26] from the silicon substrate or reconstructed silicon surface to the SHG signal can be excluded based on the reference measurements shown in Fig. 3b of the manuscript.

Further information about the symmetry-breaking mechanisms at the silicon–silver and silver–silica interfaces can be found in Sec. 1.7.

A contribution from the SiO_2 interface is expected to be insignificant. The incident angle at the silicon–silver interface is 12°, as stated in line 320 of the manuscript. Because the silver film is ultra thin, we can assume a similar internal angle at the silver–silicon-dioxide interface. Ref. [32] reports conversion efficiencies of less than $0.2 \times 10^{-27}\,\mathrm{m^2/W}$ for a single interface at this angle, while our experimentally observed values range from $20 \times 10^{-27}\,\mathrm{m^2/W}$ to more than $1000 \times 10^{-27}\,\mathrm{m^2/W}$. Thus, any SHG contribution from the SiO_2 interface is negligible compared to the response of the silver film, even considering the different wavelength used in [32].

We have clarified this point in the manuscript as follows:

Line 178:

This allows us to conclude that neither SHG from the silicon surface nor quadrupole contributions play a significant role [26], while the contribution from the capping layer is expected to be significantly lower [32].

Line 362:

The influence of the capping layer on the film was investigated in our previous work. [11]

3.3

> Nevertheless, it would have been useful to reference and contrast these findings with those from "Second-Harmonic Generation Spectroscopy on Thin Films on Si (111) by Kjeld Pedersen" [4], especially the section concerning Ag/Si(111) $-$ 7×7.

We have included Ref. [4] and provided an extensive comparison with existing literature values in the SI, Tables S3 and S4, including Ref. [4] as well as additional relevant works. The highlighted changes are shown below.

> **Line 92:**
>
> Second-order nonlinearities at metallic interfaces were reported shortly after their observation in bulk crystals [33–35], and have since been extensively studied [3, 5–7, 36–39] [1, 4].

References

[1] K. Pedersen, T. G. Pedersen, and P. Morgen. Surface and interface resonances in second harmonic generation from metallic quantum wells on Si(111). *Physical Review B*, 73(12), March 2006.

[2] A. Kirilyuk, Th. Rasing, R. Mégy, and P. Beauvillain. Nonlinear magneto-optical response from quantum well states in noble metals: Double period and interface localization. *Physical Review Letters*, 77(22):4608–4611, November 1996.

[3] Thomas Garm Pedersen, Kjeld Pedersen, and Thomas Brun Kristensen. Optical second-harmonic generation from Ag quantum wells on Si(111) 7×7: Experiment and theory. *Physical Review B*, 60(20):R13997–R14000, November 1999.

[4] Kjeld Pedersen. Second-Harmonic Generation Spectroscopy on Thin Metal Films on Si(111). *Physica Scripta*, T109:96, 2004.

[5] E. K. L. Wong and G. L. Richmond. Examination of the surface second harmonic response from noble metal surfaces at infrared wavelengths. *The Journal of Chemical Physics*, 99(7):5500–5507, October 1993.

[6] C.S. Chang and J.T. Lue. Optical second harmonic generation from thin silver films. *Surface Science*, 393(1–3):231–239, December 1997.

[7] H. Hirayama, T. Kawata, and K. Takayanagi. Oscillation of the optical second-harmonic generation intensity during ag thin film growth on a Si (111) 7×7 surface. *Physical Review B*, 64(19), October 2001.

[8] Álvaro Rodríguez Echarri, Fadil Iyikanat, Sergejs Boroviks, N. Asger Mortensen, Joel D. Cox, and F. Javier García de Abajo. Nonlinear photoluminescence in gold thin films. *ACS Photonics*, 10(8):2918–2929, June 2023.

[9] T. F. Heinz, F. J. Himpsel, E. Palange, and E. Burstein. Electronic transitions at the CaF_2/Si(111) interface probed by resonant three-wave mixing spectroscopy. *Physical Review Letters*, 63(6):644–647, August 1989.

[10] Álvaro Rodríguez Echarri, Joel D. Cox, Fadil Iyikanat, and F. Javier García de Abajo. Nonlinear plasmonic response in atomically thin metal films. *Nanophotonics*, 10(16):4149–4159, 2021.

[11] Zakaria M. Abd El-Fattah, Vahagn Mkhitaryan, Jens Brede, Laura Fernández, Cheng Li, Qiushi Guo, Arnab Ghosh, Álvaro Rodríguez Echarri, Doron Naveh, Fengnian Xia, J. Enrique Ortega, and F. Javier García de Abajo. Plasmonics in atomically thin crystalline silver films. *ACS Nano*, 13(7):7771–7779, June 2019.

[12] Vahagn Mkhitaryan, Andrew P. Weber, Saad Abdullah, Laura Fernández, Zakaria M. Abd El-Fattah, Ignacio Piquero-Zulaica, Hitesh Agarwal, Kevin García Díez, Frederik Schiller, J. Enrique Ortega, and F. Javier García de Abajo. Ultraconfined plasmons in atomically thin crystalline silver nanostructures. *Advanced Materials*, December 2023.

[13] H. J. Simon, D. E. Mitchell, and J. G. Watson. Optical second-harmonic generation with surface plasmons in silver films. *Physical Review Letters*, 33(26):1531–1534, December 1974.

[14] A. Bouhelier, M. Beversluis, A. Hartschuh, and L. Novotny. Near-field second-harmonic generation induced by local field enhancement. *Physical Review Letters*, 90(1), January 2003.

[15] A. Rodríguez Echarri, Joel D. Cox, Renwen Yu, and F. Javier García de Abajo. Enhancement of nonlinear optical phenomena by localized resonances. *ACS Photonics*, 5(4):1521–1527, January 2018.

[16] Rinu Abraham Maniyara, Daniel Rodrigo, Renwen Yu, Josep Canet-Ferrer, Dhriti Sundar Ghosh, Ruchirej Yongsunthon, David E Baker, Aram Rezikyan, F Javier García de Abajo, and Valerio Pruneri. Tunable plasmons in ultrathin metal films. *Nature Photonics*, 13(5):328, 2019.

[17] Cong-Cong Zhang, Jia-Yi Zhang, Jing-Ru Feng, Si-Ting Liu, Si-Jing Ding, Liang Ma, and Qu-Quan Wang. Plasmon-enhanced second harmonic generation of metal nanostructures. *Nanoscale*, 2024.

[18] Ajay Nahata, Richard A. Linke, T. Ishi, and K. Ohashi. Enhanced nonlinear optical conversion from a periodically nanostructured metal film. *Optics Letters*, 28(6):423, March 2003.

[19] Bernd Metzger, Lili Gui, Jaco Fuchs, Dominik Floess, Mario Hentschel, and Harald Giessen. Strong enhancement of second harmonic emission by plasmonic resonances at the second harmonic wavelength. *Nano Letters*, 15(6):3917–3922, May 2015.

[20] Gourav Bhowmik, Yong Q. An, Sandra Schujman, Alain C. Diebold, and Mengbing Huang. Optical second harmonic generation from silicon (100) crystals with process tailored surface and embedded silver nanostructures for silicon nonlinear nanophotonics. *Journal of Applied Physics*, 128(16), October 2020.

[21] Lukas Ohnoutek, Ben J. Olohan, Robin R. Jones, Xuezhi Zheng, Hyeon-Ho Jeong, and Ventsislav K. Valev. Second harmonic rayleigh scattering optical activity of single ag nanohelices in a liquid. *Nanoscale*, 14(10):3888–3898, 2022.

[22] Ben J. Olohan, Emilija Petronijevic, Ufuk Kilic, Shawn Wimer, Matthew Hilfiker, Mathias Schubert, Christos Argyropoulos, Eva Schubert, Samuel R. Clowes, G. Dan Pantoş, David L. Andrews, and Ventsislav K. Valev. Chiroptical second-harmonic tyndall scattering from silicon nanohelices. *ACS Nano*, 18(26):16766–16775, June 2024.

[23] Ufuk Kilic, Matthew Hilfiker, Alexander Ruder, Rene Feder, Eva Schubert, Mathias Schubert, and Christos Argyropoulos. Broadband enhanced chirality with tunable response in hybrid plasmonic helical metamaterials. *Advanced Functional Materials*, 31(20), February 2021.

[24] I. A. Kolmychek, I. V. Malysheva, V. B. Novikov, A. P. Leontiev, K. S. Napolskii, and T. V. Murzina. Phase-matched optical second harmonic generation in a hyperbolic metamaterial based on silver nanorods. *Physical Review B*, 102(24), December 2020.

[25] Thomas Dickinson, Ivi Afxenti, Giedre Astrauskaite, Lennart Hirsch, Samuel Nerenberg, Ottavia Jedrkiewicz, Daniele Faccio, Caroline Müllenbroich, Alessandra Gatti, Matteo Clerici, and Lucia Caspani. Quantum-enhanced second harmonic generation beyond the photon pairs regime. *Science Advances*, 11(27), July 2025.

[26] Massimo Cazzanelli and Joerg Schilling. Second order optical nonlinearity in silicon by symmetry breaking. *Applied Physics Reviews*, 3(1):011104, March 2016.

[27] W. E. Lawrence. Electron-electron scattering in the low-temperature resistivity of the noble metals. 13:5316–5319, 1976.

[28] N. W. Ashcroft and N. D. Mermin. *Solid State Physics*. Harcourt College Publishers, Philadelphia, 1976.

[29] E. P. O'Reilly. *Quantum theory of solids*. Taylor & Francis, New York, 2017.

[30] P. B. Johnson and R. W. Christy. Optical constants of the noble metals. 6:4370–4379, 1972.

[31] Zhibin Lin, Leonid V Zhigilei, and Vittorio Celli. Electron-phonon coupling and electron heat capacity of metals under conditions of strong electron-phonon nonequilibrium. *Physical Review B—Condensed Matter and Materials Physics*, 77(7):075133, 2008.

[32] Fatemeh Abtahi, Pallabi Paul, Sebastian Beer, Athira Kuppadakkath, Anton Pakhomov, Adriana Szeghalmi, Stefan Nolte, Frank Setzpfandt, and Falk Eilenberger. Enhanced surface second harmonic generation in nanolaminates. *Optics Express*, 31(7):11354, March 2023.

[33] Fielding Brown, Robert E. Parks, and Arthur M. Sleeper. Nonlinear optical reflection from a metallic boundary. *Physical Review Letters*, 14(25):1029–1031, June 1965.

[34] N. Bloembergen, R. K. Chang, S. S. Jha, and C. H. Lee. Optical second-harmonic generation in reflection from media with inversion symmetry. *Physical Review*, 174(3):813–822, October 1968.

[35] P. A. Franken, A. E. Hill, C. W. Peters, and G. Weinreich. Generation of optical harmonics. *Physical Review Letters*, 7(4):118–119, August 1961.

[36] Joseph Rudnick and E. A. Stern. Second-harmonic radiation from metal surfaces. *Physical Review B*, 4(12):4274–4290, December 1971.

[37] C. K. Chen, A. R. B. de Castro, and Y. R. Shen. Surface-enhanced second-harmonic generation. *Physical Review Letters*, 46(2):145–148, January 1981.

[38] J. L. Coutaz, D. Maystre, M. Nevière, and R. Reinisch. Optical second-harmonic generation from silver at 1.064-μm pump wavelength. *Journal of Applied Physics*, 62(4):1529–1531, August 1987.

[39] J. Sipe, D. Moss, and H. van Driel. Phenomenological theory of optical second- and third-harmonic generation from cubic centrosymmetric crystals. *Physical Review B*, 35(3):1129–1141, January 1987.

Response to the Referees' reports

In the following, the Referee's comments are presented in grey boxes. For transparency, we have included in the Reference section at the end of the document all references cited by the Referees. Our responses to the Referee's comments are given in plain text. Changes made to the manuscript are highlighted in light blue boxes. Line numbers in these boxes refer to the initially submitted manuscript. Within the light blue boxes, text newly added in the first revision is marked in green, while removed text is shown in red with strikethrough. For consistency and transparency, we retain this style in the second revision: newly added text is marked in dark green, and removed text is indicated in brown with strikethrough.

1 Reviewer 1

> In the revised manuscript, the authors have partially addressed the previously raised concerns. The main text now includes references to earlier work by Pedersen and others regarding the observed correlation between the SHG intensity of crystalline silver films and their thickness. Based on the authors' response, the primary novelty of the present study lies in the fabrication and investigation of epitaxial films, as well as in the development of an extended theoretical model presented in the supplementary information of the revised version. At this stage, the manuscript may be considered for publication, provided that the following points are addressed.

We thank Reviewer 1 for revisiting the revised manuscript and our initial response to the Reviewers, and for recognizing the novelty of our work, particularly in the fabrication and investigation of epitaxial films, as well as in the development of the extended theoretical model. We are pleased that Reviewer 1 is considering our manuscript for publication, subject to addressing the following points. In the following, we carefully address the remaining comments raised by Reviewer 1.

1.1

> The main manuscript does not clearly convey that the correlation between SHG intensity in crystalline silver films and their thickness has already been experimentally studied over a much broader range of layer numbers than what is presented here. Reference 41 [1] is cited only in the context of a phenomenological model and appears just in the supplementary information. A more explicit discussion of prior literature and its findings in the main text would be necessary to enable readers to properly evaluate the novelty and significance of the present work.

We thank Reviewer 1 for raising this point. We have included the following text in the main manuscript:

> **Line 120:**
>
> We provide both extensive material characterizations and quantitative nonlinear-optical characterizations. ~~This combination, together with a level of theory that goes beyond the phenomenological QWS model discussed in previous work~~ [1–4]~~, is rarely achieved and enabling experimental access to second-order nonlinear susceptibilities in the mesoscopic regime of noble metals.~~ In contrast to previous studies that rely on phenomenological QWS models [1–4] and do not provide comprehensive structural characterization of the films [1, 3–5], we combine high-quality epitaxial films, verified by detailed material characterization, with a quantitative analytical description of quantum-well–induced nonlinearities in noble-metal films. Moreover, several earlier works do not quantify SHG conversion efficiencies [1, 3, 4], while others report SHG conversion efficiencies without extracting second-order

nonlinear susceptibilities [1–4]. Our approach enables a consistent extraction of second-order nonlinear susceptibilities, establishing a direct and quantitative link between experiment and theory for SHG engineering in the mesoscopic regime of noble metals.

1.2

Although the title refers to the investigation of atomically thin silver films, the experimental and theoretical analyses presented in the main text are limited to thicknesses above 10 monolayers (10–30 ML). Referring to these samples as few-atom-thick silver films, as done in the manuscript, is therefore more appropriate. In light of prior work summarized in Table S3 of the supplementary information, which explores significantly thinner regimes, it would be advisable to revise the manuscript title to accurately reflect the studied thickness range, for example, "Few-atom-thick silver films ...".

We have changed the title as suggested to:

Line 1:

~~Atomically thin silver films for enhanced nanoscale nonlinear optics~~ Few-atom-thick silver films for enhanced nanoscale nonlinear optics

1.3

We understand that the fabrication of samples with other layer numbers to differentiate an oscillatory behavior of $\chi^{(2)}$ as observed earlier would be technically demanding. Perhaps this question could be addressed by including additional theoretical data points in Figure 4d.

We thank Reviewer 1 for this suggestion. The experimental data in Fig. 4d correspond to five film thicknesses, while the theoretical curve already contains additional thickness values in the same experimentally relevant range: 10, 11, 12, 15, 17, 20, 22, 25, and 30 ML while our model only captures integer numbers of monolayers. Previously reported oscillatory behavior is observed to be pronounced for thicknesses below 10 ML [2, 3], with reduced modulation up to approximately 20 ML thicknesses [1].

We agree that the logarithmic scale in Fig. 4d makes the thickness-dependent variations less apparent. For this reason, the corresponding theoretical results are also shown on a linear scale in Fig. S12, where the thickness dependence is more visible. While our calculations on a linear scale support an oscillatory behavior, our phenomenological quantum-well model is not intended to reproduce the detailed layer-by-layer SHG oscillations reported in earlier experiments [1–3]. Such oscillatory behavior is known to depend on several coupled effects, including thickness-dependent quantum-well states, symmetry-dependent optical selection rules, and interference between surface and interface nonlinear contributions.

The present model captures vertical quantum confinement and distinguishes the parity of the number of layers, but it does not explicitly include the full atomistic symmetry and electronic structure of the Ag unit cell. A more complete treatment would require explicit evaluation of dipole transition matrix elements and the thickness-dependent electronic density of states, which together determine symmetry-allowed optical transitions and contribute to the oscillatory SHG response observed experimentally. However, incorporating such atomistic details is computationally demanding because of the very large number of bands and k-points required for accurate Brillouin-zone integration.

In the present model, crystallographic information enters only effectively, for example through the quantum-well effective masses extracted from DFT. We have therefore added a clarification that the theory is used to capture the dominant increasing trend of the nonlinear response with decreasing thickness, rather than the detailed layer-by-layer oscillatory pattern. To clarify this, we have included the following text in the main manuscript:

> **Line 249:**
>
> Our calculations with additional thicknesses partially capture the well-known oscillatory behavior on the linear scale shown in SI Fig. S12. It should be noted, however, that a more comprehensive theoretical treatment would be required to properly reproduce the detailed layer-by-layer oscillatory behavior. The present calculations are intended to capture the dominant increasing trend of the nonlinear response with decreasing thickness.

1.4

> Line 308 should probably read Fig. 4d instead of Fig. 4e.

We thank Reviewer 1 for carefully reviewing our manuscript and for identifying this typo. This issue indeed required correction and originated from the earlier modification of Fig. 4 during the first revision. We have corrected it accordingly as follows:

> **Line 246:**
>
> The calculated second-order susceptibilities exhibit a marked increase as the number of layers decreases ~~(Fig. 4e)~~(Fig. 4d), in agreement with the trend observed experimentally.

2 Reviewer 2

> I would like to thank the authors for their detailed and thoughtful responses, as well as for the substantial revisions made to the manuscript. The clarity and overall quality of the work have improved significantly. In particular, the revisions addressing normalization of SHG efficiencies, inclusion of error bars, and expanded discussion of the experimental results and theoretical framework have strengthened the manuscript.
> The combined experimental and theoretical effort presents an interesting platform for investigating nonlinear optical responses in ultra-thin metallic systems. The results are now more clearly presented, and the main conclusions are better supported than in the initial submission.
>
> As a follow up, I have a few minor/major comments that, if addressed, would further improve the clarity and rigor of the manuscript:

We thank Reviewer 2 for the detailed assessment and valuable feedback, which has provided us with the opportunity to further strengthen the manuscript. We are pleased that Reviewer 2 recognizes that the combined experimental and theoretical efforts presented here constitute an interesting platform for investigating nonlinear optical responses in ultra-thin metallic systems, thereby highlighting the significance of our work. In the following, we address the remaining points raised by Reviewer 2.

2.1

2.1.1

> QWS–SHG connection: The ARPES data, including the energy distribution curves provided in the Supplementary Information, convincingly establish the presence and thickness dependence of quantum-well states (QWS) in the Ag films. However, the connection between these states and the observed SHG enhancement remains largely qualitative. Clarifying that the ARPES results establish the electronic-structure basis for the proposed mechanism, while the link to SHG enhancement is inferred rather than quantitatively demonstrated, would improve the interpretation.

We understand and share the view of Reviewer 2 that a quantitative analysis of the ARPES data and the corresponding energy distribution curves would be valuable for establishing a direct connection to the theoretical model presented. This is indeed a promising direction for future work. However, we note several challenges when it comes to the comparison to experimental work. While ARPES and EDC data can be analyzed qualitatively, a fully quantitative analysis of the present dataset would involve substantial uncertainties arising from underlying ARPES measurements. Such an approach would require subsequent DFT simulations based on these measurements, which would then serve as input for modeling the nonlinear conversion efficiencies in our optical framework. This multi-step procedure entails considerable computational and methodological effort that goes beyond the scope of the present study. Moreover, the propagation of uncertainties through these steps would likely weaken the comparison with experimental data. We nevertheless appreciate this suggestion and consider it a valuable motivation for future investigations.

2.1.2

> QWS-to-continuum crossover: Moreover, Fig. 2 clearly demonstrates the presence of quantum-well states and their thickness dependence. However, the transition from discrete levels in thin films to a quasi-continuous electronic structure in thicker films, as illustrated conceptually in Fig. 1, is not directly evident in the experimental data, where the subbands remain well resolved even at higher thicknesses. Am I missing something that has already existed in the text if not I kindly recommend, a brief clarification distinguishing the conceptual illustration from the experimentally accessible regime would improve clarity.

We thank Reviewer 2 for the opportunity to further elaborate on the QWS shown in Fig. 1a and Fig. 2a/b of the main text. In Fig. 1a, we present the simulated electronic density of states along the out-of-plane k_z direction, which differs from the dispersion shown in Fig. 2a/b. The choice to show the out-of-plane k_z direction is intentional, as the dominant contribution to the nonlinear optical response arises from the out-of-plane component, as discussed in the main text.

Moreover, Fig. 1a represents the Ag s, p band discretized along the Γ–L direction (i.e., along the z-axis), yielding N quantum well levels for an N-layer film. These QWS, depicted as horizontal lines in Fig. 1a, correspond to the energies at the center ($k_\parallel = 0$) of the two-dimensional bands shown in Fig. 2a/b.

In Fig. 2a/b, we do not display the full s, p band, as it extends to binding energies of approximately $-10\,\mathrm{eV}$. Moreover, below $-4\,\mathrm{eV}$, the band structure is strongly influenced by d-band hybridization, characterized by weak dispersion and high photoemission intensity, which obscures the features of interest. Considering the $\sim 3\,\mathrm{eV}$ energy window shown, this corresponds to roughly 3/10 of the Γ–L Brillouin zone. One would therefore expect on the order of nine quantized levels, which is in very good agreement with the eight bands observed in Fig. 2b. This consistency supports the agreement of the representation in Fig. 1a.

We have clarified this point and improved the accessibility of the discussion for the reader through the following changes to the manuscript. In addition, we also corrected a typo that may have contributed to the missing clarity.

Line 84:

Electrons are then confined to the QWS, resulting in discrete bands [6] with less out-of-plane harmonic motion than in thicker crystalline structures, where electrons exhibit a parabolic out-of-plane dispersion relation. The subpanels of Fig. 1a show the simulated electronic density of states, where in ultra-thin silver films the s, p band is discretized along the Γ–L direction, yielding N quantum well levels for an N-layer film. Discretization is expected for thicknesses up to approximately 100 ML before reaching the continuous dispersion relation [7] established when the intrinsic broadening of QWS exceeds their energy separation.

Line 150:

Here, ARPES characterizes the in-plane wave vector $k_\parallel$, whereas Fig. 1a displays the simulated electronic density of states along the out-of-plane direction k_z. QWS, depicted as horizontal lines in Fig. 1a, correspond to the energies at the center ($k_\parallel = 0$) of the two-dimensional bands shown in Fig. 2a/b.

Following the notation of Ref. [8], we evaluate the density of states as

$$\mathrm{DOS}(\omega, k_z) = \sum_{\hbar\varepsilon_j^\perp \leq E_\mathrm{F}} |\varphi_j(k_z)|^2 L(\omega - \varepsilon_j^\perp), \tag{1}$$

where $\varphi_j(k_z)$ is the Fourier transform of the out-of-plane wave function along the confinement direction. Each occupied quantum-well state is weighted by a Lorentzian distribution $L(\omega)$ with linewidth γ, representing the inelastic scattering rate of the conduction electrons. For silver, we use $\hbar\gamma = 21\,\mathrm{meV}$.

We have included the reference in the caption of Fig. 1.

Line 1 in caption of Fig. 1:

The simulated electronic density of states [8], represented here as a function of ~~in-plane~~out-of-plane wave vector k_z (bottom, horizontal scale) and energy (vertical state), shows an evolution from discrete quantum-well states (QWS) in thin films ($N = 10$ atomic monolayers (ML)) to a coalescing band at larger thicknesses ($t = 30\,\mathrm{ML}$).

2.2

Polarization and symmetry considerations: The authors explain that excitation through the silicon substrate preserves predominantly p polarization, which justifies the focus on p polarized excitation and the absence of a full polarization dependent SHG study.

We thank Reviewer 2 for understanding the limitations of our polarization analysis capabilities due to these constraints.

2.2.1

> However, it would be helpful to more explicitly state the implications of this limitation for the interpretation of the results. In particular, the lack of polarization resolved SHG measurements prevents the separation of different contributions to the nonlinear response, for example interface versus field induced effects, and precludes access to individual tensor components of the second order susceptibility. A brief clarification alongthese lines would help guide the reader on the scope of the conclusions.

We agree with Reviewer 3 that this point can be stated more explicitly and its implications clarified for the reader. We have therefore added the following line to the manuscript:

> **Line 189:**
>
> The limitations in polarization tomography restrict access to the extraction of individual tensor components of the second-order nonlinear susceptibility tensor.

> **Line 251:**
>
> Access to the different mechanisms could be achieved in the future through polarization-resolved and thickness-dependent SHG characterization of the films.

2.2.2

> In addition, while Fig. S15 provides useful information on the polarization state after transmission through the silicon substrate, presented on a Poincare sphere based on reported Jones vectors, it is not immediately clear whether these results are obtained experimentally or derived from a model, and the methodology used to determine the Jones vectors is not described. A short clarification of how the polarization state was determined would improve transparency.

We agree with Reviewer 2 that greater transparency in this regard is warranted. We have therefore added a section named "Determination of polarization state" to the Supporting Information in which we elaborate on the methods used. We have included the following:

> **SI:**
>
> Due to the substrate tilt and its non–polarization-preserving behavior for non-p-polarized light, only p-polarized excitation was used. The polarization transformation induced by the bare silicon substrate was characterized by polarization tomography. Consequently, the excitation polarization was fixed to p-polarization, and a quarter-wave plate placed in front of a linear polarizer was rotated after the sample and before the polarization independent detector. Using the characterized quarter-wave plate and polarizer, the intensity modulation of the detected fundamental excitation beam could be modeled while correcting for Fresnel coefficients.
> Taking into account the modulation of the Fresnel reflections at the interfaces, the polarization state can be described using the following expression [9, 10]:
>
> $$I = \frac{I_0}{2}\left[S_0 - S_1\cos^2(2\theta) + S_2\cos(2\theta)\sin(2\theta) + S_3\sin(2\theta)\right]. \quad (2)$$
>
> Equation 2 directly relates the detected intensity I to the maximum intensity I_0 and the rotation angle of the quarter-wave plate θ to the Stokes parameters $S = (S_0, S_1, S_2, S_3)$ of the excitation beam

after transmission through the bare silicon sample. These parameters can subsequently be converted into the corresponding Jones vector representation, as presented in Fig. S15. The Fresnel reflections were characterized by measuring the transmitted excitation power through the sample as a function of the linear excitation polarization. The refractive index of silicon at a wavelength of $2.3\,\mu$m was taken as $\tilde{n}_{\mathrm{Si}} = 3.47 + i6.57 \cdot 10^{-8}$ [11], while for air $\tilde{n}_{\mathrm{Air}} = 1$ [12] was assumed.

2.3

Uncertainty at larger thicknesses: In Fig. 4 (particularly panel d), the experimental uncertainty appears to increase noticeably for larger film thicknesses (e.g., $t \approx 30\,\mathrm{ML}$). While this is likely related to reduced SHG signal strength and associated signal-to-noise limitations, this behavior is not explicitly discussed. A brief clarification regarding the origin of the increased uncertainty and its implications for interpreting the thickness dependence would improve the presentation. In addition, in Fig. 4d and Figs. S12 and S13, the theoretical results show instances where the responses for different excitation wavelengths appear to overlap at certain thicknesses, while at neighboring thickness values they diverge more noticeably. It would be helpful to briefly clarify the physical origin of this behavior, in particular whether it arises from thickness dependent shifts in quantum well state energies, variations in resonance conditions, or model assumptions such as fixed damping.

The experimental uncertainty in Fig. 4 arises from two primary sources. The first is associated with the signal-to-noise ratio relative to the detector dark count rate, as well as power fluctuations and small center-wavelength drifts of the excitation beam during the measurement procedure. The second contribution is of statistical origin and reflects spatial inhomogeneities of the films, quantified by the standard deviation of measurements taken at different regions for a given thickness. In general, fluctuations related to the excitation beam constitute the dominant contribution, while fabrication-induced spatial variations are smaller. For thicker films, the reduced SHG signal strength leads to a lower signal-to-noise ratio, making detector noise increasingly significant and resulting in larger uncertainties compared to thinner films.

The overlap of the theoretical curves at selected thicknesses arises from quantum-well-state resonances shifting with film thickness and crossing the fixed excitation wavelengths. The leading quantitative parameter is the conduction-electron density, while the precise resonance positions are also sensitive to the effective-mass description of the quantum-well subbands, as discussed previously in our electron energy-loss spectroscopy study of atomically thin metallic films [13]. The homogeneous damping used in the model broadens these resonances and can smooth nearby features. The role of the different model approximations is discussed in more detail in our response in Sec. 2.4, where we have added the corresponding clarifications.

We have made the following changes to address these points in the manuscript:

Line 206:

The experimental uncertainty in Fig. 4 arises from spatial inhomogeneities of the films (statistical variations across probed regions) and fluctuations of the excitation beam, except at low SHG signal levels for thicker films, where detector noise becomes significant.

2.4

Dielectric function and Model scope limitations: and quantitative deviations: The manuscript appropriately emphasizes that the theoretical model is intended to capture the dominant physical trends rather than provide a fully quantitative description. Given the observed discrepancies between experiment and theory, it would be helpful to briefly clarify which physical parameters (e.g., damping, effective masses, or interface contributions) are expected to have the largest impact on the quantitative agreement. Such a discussion would help guide the reader on how the present phenomenological framework could be extended in future work without requiring additional fitting in the current manuscript.

We thank the Reviewer for this useful suggestion. We agree that the scope and main quantitative limitations of the phenomenological model should be made more explicit. We have therefore expanded the discussion in the main text to clarify which model assumptions are expected to have the largest effect on the quantitative comparison with experiment.

Line 251:

However, the variation in calculated ~~SHG conversion efficiencies~~ second-order nonlinear susceptibility with incident light wavelength differs from the experimental results. This discrepancy originates from the assumptions underlying the theoretical model. In particular, the model employs parabolic quantum-well subbands, neglects the presence of the substrate, and assumes a single, energy-independent linewidth. While the effective masses used in the model are extracted from density-functional-theory (DFT) calculations by fitting the low-energy dispersion of the quantum-well states to parabolas, the full DFT band structures reveal deviations from a harmonic behavior. The effective-mass approximation is therefore another important source of quantitative uncertainty: the SHG response is resonantly sensitive to the energy spacing and curvature of the quantum-well states, so small deviations from parabolicity can shift or reshape the calculated spectral features [13]. Moreover, the interaction with the Si substrate modifies the band dispersions and effective masses, thereby altering the carrier dynamics. A further limitation of the model is that the response of the deeper d bands is included only through an effective dielectric background rather than through a fully atomistic description. This approximation captures the dominant screening contribution of core electrons, but it cannot reproduce possible facet-, thickness-, or interface-dependent modifications of the interband response. ARPES data in Fig. 2 further demonstrate that the electronic linewidths are subband- and energy-dependent rather than uniform. Although surface-enabled Landau damping is implicitly captured in the model through an effective phenomenological linewidth, its detailed energy-, subband-, and thickness-dependent character is not explicitly resolved. The use of a homogeneous damping rate can therefore affect both the amplitude and the spectral width of the calculated SHG resonances. Since the SHG response is highly sensitive to carrier density, subband energies, effective masses, core-electron screening, and electronic lifetimes, quantitative deviations in both the magnitude and spectral position of the SHG features are therefore expected. In addition, thickness-dependent ~~ultra-fast~~ultrafast thermal and electronic broadening effects in the silver film [14] and the environment, which are not included in the present model, may further contribute to the observed differences between theory and experiment (see Sec. III in the SI). We emphasize that the purpose of the present model is to capture the dominant physical trends governing the thickness dependence of the SHG response rather than to provide a fully quantitative description. A more complete treatment would necessitate substantially more complex theoretical and computational methods including realistic nonparabolic band structures, an atomistic treatment of d-band screening, and state-resolved damping, which lies beyond the scope of the present work.

2.5

2.5.1

> Interpretation of theoretical spectral features and Extension beyond experimentally accessible thickness range: The theoretical results in Figs. S11 and S12 explore thickness regimes that extend to or approach values below those experimentally realized in the present study. It would be helpful if the authors could briefly comment on how the predicted nonlinear response is expected to evolve in such ultra thin regimes, and how these idealized predictions should be interpreted in light of experimental constraints such as film continuity, increased disorder, and structural inhomogeneity. In particular, a short discussion clarifying whether the strong spectral features predicted by the model at very low thicknesses would remain observable or become broadened or suppressed under realistic conditions would help guide the reader in assessing the physical relevance of the theoretical extrapolation.

We agree that the idealized calculations should be interpreted with care near the lowest thicknesses. The model assumes laterally homogeneous, crystalline Ag(111) films with well-defined quantum-well states, a thickness-independent phenomenological damping rate, and no explicit disorder, island formation, roughness, or substrate-induced inhomogeneity in their band structure. Under realistic experimental conditions, any loss of film homogeneity, increased disorder, thickness variations, or spatially inhomogeneous coupling to the substrate would broaden the calculated spectral features and could suppress the sharp resonances predicted by the idealized model. Thus, the strong narrow spectral structures in Figs. S11 and S12 should be understood as signatures of the underlying quantum-well-state physics in an idealized crystalline film, rather than as quantitatively resolved spectral predictions for structurally imperfect ultra-thin samples. Our simulations predict that *thinner films yield a larger SHG response*, which is a message already discussed in the Section ”Measured SHG signal”, and in the Section ”Discussion” of the main text.

We have added a short clarification to the Supplementary Information to reinforce that message when presenting the results in Figs. S11 and S12.

> **Line Caption Fig. S12:**
>
> We emphasize that the theoretical calculations in Figs. S11 and S12 are intended to describe the experimentally relevant thickness regime of continuous crystalline Ag(111) films. They should not be interpreted as quantitative predictions for structurally discontinuous films or for thicknesses below the stability limit of the growth procedure. The simulations assume laterally homogeneous films with well-defined quantum-well states and a phenomenological damping rate. The narrow resonances predicted by the model therefore represent the intrinsic quantum-well-state contribution expected in idealized crystalline films.

2.5.2

> Clarification of Fig. 1: It would be helpful to explicitly clarify whether Fig. 1 represents a conceptual or theoretical illustration rather than experimental data. While it effectively conveys the expected trnsition from discrete quantum well states to a quasi-continuous electronic structure with increasing thickness, this crossover is not directly evident in the experimental ARPES results shown in Fig. 2. A brief clarification in the caption or main text would improve readability and avoid potential confusion.

We thank the Reviewer 2 for pointing out that the comparison between Fig. 1 and Fig. 2 can be improved. We have clarified this in Sec. 2.1.2 and would therefore like to refer the Reviewer 1 to this section.

2.6

> Consistency of mechanistic interpretation: In the paragraph discussing the thickness dependence of the SHG response (Results section, around Fig. 4), the manuscript states: "This indicates that the rise in second-order susceptibility is due to thickness-dependent changes in the electronic band structure of the material." As the authors acknowledge that both intrinsic (quantum-well–induced band-structure modifications) and extrinsic factors (e.g., reduced reabsorption, local field enhancement, and interface contributions) contribute to the observed SHG enhancement, noting that these effects act cooperatively. For consistency, it would be beneficial to clarify this and to reflect the combined role of these mechanisms already described in the manuscript.

We thank Reviewer 2 to making this inconsistency obvious to us. We have made the following changes to the manuscript following this suggestion:

> **Line 249:**
>
> ~~This indicates that the rise in second-order susceptibility is due to thickness-dependent changes in the electronic band structure of the material.~~ Therefore, these results indicate that the increase in second-order susceptibility arises from thickness-dependent changes across the entire film. The observed changes arise from intrinsic factors such as modifications of the QWS band structure, regions with symmetry breaking, and quantum confinement, as well as extrinsic mechanisms such as field enhancement and reduced fundamental reabsorption.

3 Reviewer 3

> The revised manuscript has satisfied my original concerns. I support publication.

We thank Reviewer 3 for reviewing our manuscript and are pleased that the revised version satisfactorily addresses the original concerns raised by in their review.

References

[1] K. Pedersen, T. G. Pedersen, and P. Morgen. Surface and interface resonances in second harmonic generation from metallic quantum wells on Si(111). *Physical Review B*, 73(12), March 2006.

[2] Thomas Garm Pedersen, Kjeld Pedersen, and Thomas Brun Kristensen. Optical second-harmonic generation from Ag quantum wells on Si(111) 7×7: Experiment and theory. *Physical Review B*, 60(20):R13997–R14000, November 1999.

[3] H. Hirayama, T. Kawata, and K. Takayanagi. Oscillation of the optical second-harmonic generation intensity during ag thin film growth on a Si (111) 7×7 surface. *Physical Review B*, 64(19), October 2001.

[4] Kjeld Pedersen. Second-Harmonic Generation Spectroscopy on Thin Metal Films on Si(111). *Physica Scripta*, T109:96, 2004.

[5] C.S. Chang and J.T. Lue. Optical second harmonic generation from thin silver films. *Surface Science*, 393(1–3):231–239, December 1997.

[6] A. L. Wachs, A. P. Shapiro, T. C. Hsieh, and T.-C. Chiang. Observation of film states and surface-state precursors for ag films on si(111). *Physical Review B*, 33(2):1460–1463, January 1986.

[7] T.-C Chiang. Photoemission studies of quantum well states in thin films. *Surface Science Reports*, 39(7-8):181–235, 2000.

[8] Álvaro Rodríguez Echarri, Joel D. Cox, Fadil Iyikanat, and F. Javier García de Abajo. Nonlinear plasmonic response in atomically thin metal films. *Nanophotonics*, 10(16):4149–4159, 2021.

[9] Beth Schaefer, Edward Collett, Robert Smyth, Daniel Barrett, and Beth Fraher. Measuring the stokes polarization parameters. *American Journal of Physics*, 75(2):163–168, February 2007.

[10] M. Chaturvedi, R. Bhandare, S. Kumar, Y. Verma, and S. Raja. A compact full stokes polarimeter. *Optik*, 267:169645, October 2022.

[11] E. Shkondin, O. Takayama, M. E. Aryaee Panah, P. Liu, P. V. Larsen, M. D. Mar, F. Jensen, and A. V. Lavrinenko. Large-scale high aspect ratio al-doped zno nanopillars arrays as anisotropic metamaterials. *Optical Materials Express*, 7(5):1606, April 2017.

[12] Richard J Mathar. Refractive index of humid air in the infrared: model fits. *Journal of Optics A: Pure and Applied Optics*, 9(5):470–476, April 2007.

[13] A Rodríguez Echarri, Enok Johannes Haahr Skjølstrup, Thomas G Pedersen, and F Javier García de Abajo. Theory of electron energy-loss spectroscopy in atomically thin metallic films. *Physical Review Research*, 2(2):023096, 2020.

[14] Álvaro Rodríguez Echarri, Fadil Iyikanat, Sergejs Boroviks, N. Asger Mortensen, Joel D. Cox, and F. Javier García de Abajo. Nonlinear photoluminescence in gold thin films. *ACS Photonics*, 10(8):2918–2929, June 2023.
